# The Structure, Evolution, and Comparative Genomics of the *Dendrobium officinale* Mitochondrial Genome

**DOI:** 10.3390/biology15191763

**Published:** 2026-10-03

**Authors:** Zemei Zhu, Jiamei Zheng, Yue Zhang, Chunfan Xiang, Shiqing Xie, Feiya Zhao, Aien Tao

**Affiliations:** College of Medicine, Lijiang Culture and Tourism College, Lijiang 674100, China; zemei0910@126.com (Z.Z.); zjmeijm@126.com (J.Z.); zwtg1328@126.com (Y.Z.); 17869407534@163.com (C.X.); notexie@163.com (S.X.)

**Keywords:** *Dendrobium officinale*, mitochondrial genome, comparative analysis, MTPTs, RNA editing

## Abstract

*Dendrobium officinale* is an important medicinal Orchidaceae species with considerable ecological and economic value. While its chloroplast genome has been well characterized, the structure features of its mitochondrial genome remain largely unexplored, which further hinders our understanding of its evolutionary dynamics and adaptive mechanisms. This study presents a comprehensive characterization of the mitochondrial genome of *D. officinale*, revealing a complex multibranched structure of 22 chromosomes totaling 639,931 bp, encoding 35 PCGs, 3 rRNA genes, and 24 tRNA genes. Codon usage analysis revealed a distinct codon bias, while the genome harbors abundant repetitive elements and 522 RNA editing sites. Comparative genomic analysis identified 125 MTPT fragments totaling 108,012 bp (16.88% of the mitogenome), indicating frequent inter-organellar DNA transfer. *Dendrobium nobile* exhibited the highest genomic homology with *D. officinale*. Ka/Ks analysis indicated that most mitochondrial genes are under strong purifying selection. In addition, we conducted a comparative analysis between the newly assembled mitochondrial genome of *D. officinale* and seven Orchidaceae species. Phylogenetic analysis based on the maximum likelihood, maximum parsimony and bayesian inference approaches resolved the phylogenetic position of *D. officinale* within Orchidaceae. Collectively, these findings provide a valuable reference for future studies on Orchidaceae phylogenomics and mitochondrial evolution.

## Simple Summary

Orchidaceae are one of the largest and most diverse plant families on Earth, and many of these plants, such as *Dendrobium officinale*, are valued as medicines and ornamental plants. While the chloroplast DNA of Orchidaceae species has been studied extensively, their mitochondrial DNA remains poorly understood, even though it plays essential roles in energy production and stress responses. Mitochondrial genomes in plants are unusually large and complex, which makes them difficult to study but also rich in evolutionary information. In this study, we used high-throughput sequencing to assemble and describe the complete mitochondrial genome of *D. officinale*. We examined its structure, gene content, repeated sequences, and RNA editing sites and compared it with related Orchidaceae species. We also used this genome to explore the evolutionary relationships of Orchidaceae species. Our results provide new genomic resources for this important medicinal species and improve our understanding of how plant mitochondrial genomes evolve. These findings may support the future breeding, species identification, and conservation of Orchidaceae germplasm.

## 1. Introduction

Orchidaceae is one of the largest and most widely distributed families of angiosperms, comprising approximately 750 genera and 28,000 species, which account for about 10% of all flowering plant species [1,2]. This family is found worldwide, occupying diverse ecological niches that range from tropical rainforests to temperate alpine regions [3]. In recent years, Orchidaceae has also become a key model system for molecular phylogenetic studies, owing to its rapid diversification, complex evolutionary patterns, and the increasing availability of genome-scale data [4]. Within this extensive family, *Dendrobium* stands out as one of the largest and most economically significant genera, encompassing over 1500 species that are prevalent in Asia and Oceania [5]. Notably, *Dendrobium officinale* is regarded as a particularly valuable medicinal plant [6]. This species, listed in the *Chinese Pharmacopoeia* and widely cultivated in East Asia, is highly esteemed for its ornamental and therapeutic properties [7].

Advances in next-generation sequencing have significantly increased throughput and reduced costs, facilitating a wide array of large-scale genomic studies. In the family Orchidaceae, most genome-level investigations have concentrated on chloroplast genomes, leading to the publication of numerous plastid genomes [8]. These datasets have provided essential insights into Orchidaceae taxonomy, systematics, and phylogenetic relationships. Despite the value of plastid data, plastid genomes alone cannot fully resolve the evolutionary and functional complexity of plant organelles. Plastid genomes are highly conserved in both size and gene arrangement, typically maintaining a quadripartite structure of approximately 120–160 kb, in which two inverted repeat regions separate the large and small single-copy regions [9,10]. In contrast, the size variation in mitochondrial genomes can span several orders of magnitude, and their structures may exhibit linear, fragmented, circular, branched, or even polychromatic configurations [10]. Moreover, whereas plastids are primarily responsible for photosynthesis, mitochondria serve as the energy factories of the cell, carrying out respiration and energy production. The mitochondrial genome encodes components of respiratory chain complexes (e.g., *sdh*, *cox*, and *nad* genes) as well as genes associated with cytoplasmic male sterility, none of which are represented in the plastid genome, and these genes are directly linked to energy metabolism and fertility regulation [11]. Owing to extensive horizontal gene transfer and frequent genome rearrangements, plant mitochondrial genomes exhibit remarkable diversity in both sequence composition and structural organization [12]. Consequently, mitochondrial genomes have emerged as important and complementary resources for species identification, phylogenetic reconstruction, and the investigation of inheritance patterns [13]. In higher plants, mitochondrial genomes are characterized by low gene density, with protein-coding regions typically accounting for only approximately 10% of the total genome, alongside abundant repetitive sequences and numerous RNA editing sites [14]. Unlike the relatively conserved structure of chloroplast genomes, plant mitochondrial genomes are notoriously complex, characterized by large sizes, extensive recombination, multipartite structures, and the frequent incorporation of foreign sequences [15,16]. They play essential roles in energy production, cytoplasmic male sterility, programmed cell death, and stress responses [17].

Despite these advantages, mitochondrial genome research in Orchidaceae remains relatively limited compared with plastid genomics, thereby constraining integrative phylogenetic and evolutionary analyses. Therefore, the assembly and comprehensive characterization of mitochondrial genomes across a broader taxonomic sampling of Orchidaceae are necessary, combined with comparative analyses of genome structure, gene content, repeat sequence, RNA editing sites, and organellar DNA transfer. Such efforts will help elucidate the structural principle and evolutionary forces shaping Orchidaceae mitogenomes, including gene rearrangement, homologous recombination, and selective constraints. Importantly, the resulting genomic resources, such as informative molecular markers, repeat-based markers, and functionally characterized genes, can be applied to marker-assisted selection, phylogenetic resolution, and germplasm improvement in Orchidaceae. Therefore, the assembly and comprehensive characterization of orchid mitochondrial genomes are essential for advancing our understanding of their genetic architecture, evolutionary dynamics, and applications in breeding and germplasm improvement.

The present study employed high-throughput sequencing to assemble and annotate the complete mitochondrial genome of *D. officinale*. We conducted a comprehensive analysis of its genome architecture, gene content, codon usage, repeat elements, RNA editing sites, and phylogenetic placement. By incorporating mitochondrial genomic data into a molecular phylogenetic framework, this study aims to provide additional context and comparison to plastid-based phylogenetic inferences in Orchidaceae. Comparative genomic approaches were further applied to evaluate its evolutionary features relative to other Orchidaceae and representative angiosperms. By integrating these analyses, we provide a detailed characterization of the *D. officinale* mitogenome and highlight its significance in advancing organelle genomics and evolutionary biology. The results of this work not only generate fundamental genomic data for *D. officinale* but also expand our broader understanding of mitochondrial genome evolution in the family Orchidaceae. Furthermore, the findings have practical implications, offering theoretical foundations and novel strategies for genetic improvement, breeding programs, germplasm resource management, and the sustainable utilization of this important medicinal species.

## 2. Materials and Methods

### 2.1. Plant Material Sampling, DNA Extraction, and Sequencing

The plant materials were collected from private farming fields in Dali, Yunnan, China (25°59′ N, 100°24′ E). A single healthy, disease-free *D. officinale* plant was selected, and three young leaves were harvested from this individual. The leaves were immediately frozen in liquid nitrogen, then pooled and ground together for total DNA extraction using a modified CTAB method [18]. Plant identification was conducted by Professor Aien Tao, with the specimens deposited in the herbarium of Lijiang Culture and Tourism College (voucher ID: DO20241219). The collection of *D. officinale* was performed with the necessary permissions. All experimental procedures, including the collection of plant materials, adhered to institutional, national, and international guidelines. The quality of DNA was evaluated using agarose gel electrophoresis, via Nanodrop spectrophotometer 2000 (Thermo Fisher Scientific, Waltham, MA, USA). The qualified DNA samples were randomly fragmented into segments using a Covaris ultrasonic disruptor and then underwent end repair, A-tailing, sequencing linker addition, purification, PCR amplification, and other steps to complete the entire library preparation. The constructed libraries were subjected to sequencing on the Illumina Novaseq and PacBio sequencer platforms by Novogene Biotech Co., Ltd. (Beijing, China). The raw sequencing data contained adapter sequences and low-quality reads, which could substantially interfere with downstream bioinformatic analyses. To ensure analytical reliability, raw reads were rigorously filtered to generate clean reads, and all subsequent analyses were based on these high-quality data. Firstly, fastp 1.3.6 was used for the quality control of Illumina double-ended sequencing data. Fastp automatically identifies and removes the splice sequence and sets the minimum base quality value as Q20 (-q 20), and each sequence is allowed to contain at most two undetermined bases (-n_base_limit 2). The trimmed sequences with a length less than 50 BP are removed (-length_required 50). At the same time, the repeat sequence evaluation function is turned off (-dont_eval_duplication), 8 threads are used for processing, and the compression level is set to 6. In addition to the above parameters, other fastp parameters adopt the software default values. The filtered clean reads were used for chloroplast genome assembly.

### 2.2. Genome Assembly and Annotation

The mitochondrial genome of *D. officinale* was assembled de novo using PMAT (version 2.1.0) with the autoMito -t hifi parameter [19]. HiFi reads were subsequently mapped to the mitochondrial genome using Minimap2 (version 2.24) to identify repetitive regions, resulting in a circular contig. The chloroplast genome was assembled from second-generation clean reads utilizing GetOrganelle 1.7.7.1 with the parameters: get_organelle, -R 30, -k 21,45,65,85,105, and -F embplant_pt. Among them, -r 30 represents 30 rounds of read recruitment and extension at most, -k 21,45,65,85,105 represents the use of multiple k-mer values for assembly, and -F embplant_pt represents the use of terrestrial chloroplast genome assembly mode. Getorganelle uses the default seed database of this version of software to recruit target reads. The quality filtering, coverage control and graph simplification parameters not specially specified are set to the software default.

All assembled mitochondrial and chloroplast genomes were initially annotated with the online tool GeSeq (https://chlorobox.mpimp-golm.mpg.de/geseq.html, accessed on 13 July 2026). Geseq uses its default MPI-MP organelle reference database and default organelle tRNA reference resources for annotation. Circular genome maps were generated using OGDRAW (https://chlorobox.mpimp-golm.mpg.de/OGDraw.html, accessed on 13 July 2026). To ensure annotation accuracy, all results were manually reviewed and corrected using Apollo (version 1.11.8). Manual correction mainly includes the gene boundary, gene direction, CDS boundary, reading frame, start codon and stop codon, exon–intron boundary, RNA editing-related annotation and trans-splicing gene structure. This manual correction is judged according to the sequence alignment of the reference gene, homologous gene structure, conservative reading frame and sequence alignment results. Finally, the complete mitochondrial genome of *D. officinale* was deposited in the GenBank database of NCBI (https://www.ncbi.nlm.nih.gov/, accessed on 25 August 2026), with accession numbers PZ861981-PZ862002.

### 2.3. Repeat Sequence Analysis and RNA Editing Analysis

SSRs in the mitochondrial genome of *D. officinale* were identified using MISA (v2.1) with default parameters (definitions: 1–10, 2–6, 3–5, 4–5, 5–5, 6–5; interruptions: 100) [20]. Tandem repeats were detected using Tandem Repeats Finder (trf409.linux64) with the parameters 2 7 7 80 10 50 500 -f -d -m [21]. Additionally, dispersed repeats were identified using Vmatch (v2.3.1) with the settings -f -p -h 3 -l 30 -allmax. RNA editing sites in the mitochondrial genome of *D. officinale* were predicted using deepredmt (v0.1.0), applying a probability threshold of 0.9 to ensure the reliability of the results. In addition to characterizing the mitochondrial genome structure of the newly sequenced *D. officinale*, this study also conducted a comparative analysis with seven other Orchidaceae species (*Apostasia fujianica*, *Dendrobium hancockii*, *Dendrobium henanense*, *Dendrobium huoshanense*, *Dendrobium loddigesii*, *Dendrobium nobile*, *Dendrobium wilsonii* (accession numbers are listed in Table S1)). This comparative analysis was performed to assess the conservation and divergence of repetitive elements (SSRs, tandem repeats, and dispersed repeats) and RNA editing sites across these species, thereby providing a broader evolutionary context for understanding the mitochondrial genome of *D. officinale*.

### 2.4. Analysis of Codon Usage

Protein-coding sequences (PCGs) were extracted using PhyloSuite with the default settings. Relative synonymous codon usage (RSCU) was calculated with CodonW based on all PCGs to evaluate codon preference [22]. Subsequently, the results were visualized using ggplot.

### 2.5. Identification of Homologous Fragment of Chloroplast and Mitochondria

Homologous sequences between the mitochondrial and chloroplast genomes were identified using BLASTN (v2.16.0), and homologous fragments were visualized using the Circos package [23]. Additionally, gene transfer events from the chloroplast to the mitochondrion were illustrated with TBtools (v2.056).

### 2.6. Phylogenetic and Collinearity Analysis

To construct phylogenetic relationships, we downloaded 22 complete mitochondrial genome sequences in the NCBI database (accession numbers are listed in Table S1) and analyzed them alongside the newly assembled mitochondrial genome of *D. officinale*. The amino acid sequences of the core genes were aligned using MAFFT (v7.505, --auto mode), with poorly aligned regions trimmed using TrimAl (v1.2rev57, automated1 setting) [24]. Phylogenetic trees were constructed with IQ-TREE (v2.2.0), employing the maximum likelihood method [25]. The optimal substitution model was automatically determined using ModelFinder (-m MFP), and branch support values were estimated through 1000 ultrafast bootstrap replicates. Beyond the maximum likelihood phylogeny, we generated additional trees using the maximum parsimony and bayesian inference approaches. These complementary analyses were performed to rigorously evaluate the consistency of the inferred phylogenetic relationships and to test the stability of the resulting topologies across different analytical frameworks. Collinearity analysis was conducted with NGenomeSyn (v1.42) [26].

### 2.7. Analysis of Non-Synonymous (Ka) and Synonymous (Ks) Mutation Rates

The Ka/Ks ratio is instrumental in determining whether PCGs are experiencing selective pressure during evolution. To evaluate the selective pressures acting on mitochondrial genes, we calculated the Ka/Ks ratios of 31 shared genes from the mitochondrial genomes of *D. officinale* and seven other Orchidaceae species (*Apostasia fujianica*, *Dendrobium hancockii*, *Dendrobium henanense*, *Dendrobium huoshanense*, *Dendrobium loddigesii*, *Dendrobium nobile*, *Dendrobium wilsonii* (accession numbers are listed in Table S1)) using the MA model implemented in KaKs_Calculator [27]. The results were visualized as box plots generated with Origin 2021. Under neutral selection, the condition Ka = Ks results in a Ka/Ks ratio of 1. A Ka/Ks ratio greater than 1 (Ka > Ks) signifies positive selection, while a ratio less than 1 (Ka < Ks) indicates negative (purifying) selection. 

## 3. Results

### 3.1. Genomic Structure of D. officinale Mitogenome

**Figure 1:**
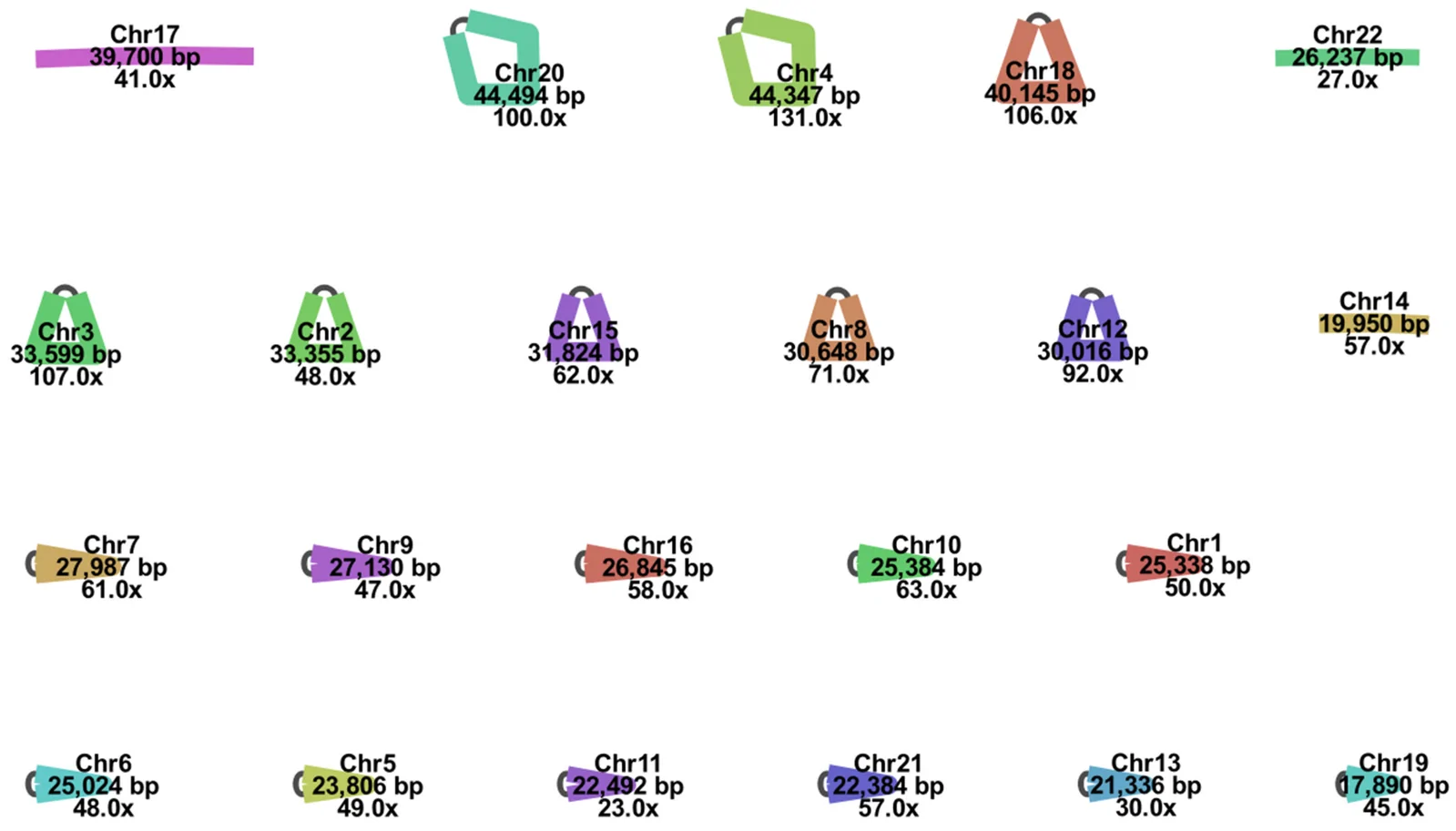
The branched conformation of the *D. officinale* mitochondrial genome. This figure shows the assembly result visualized in Bandage, displaying 22 chromosomes (19 circular and 3 linear).

**Figure 2:**
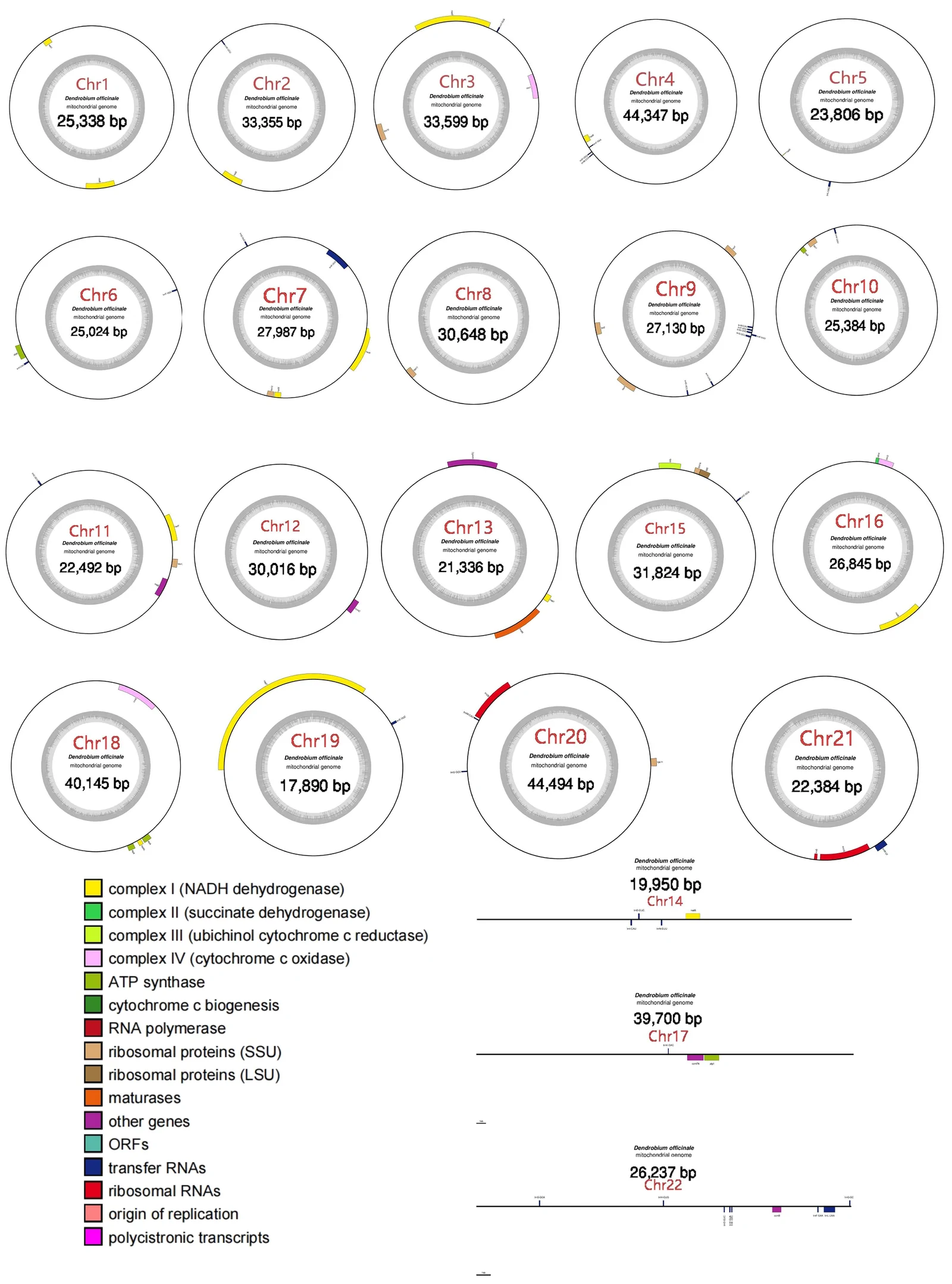
The *D. officinale* mitogenome map. Genes are classified into different categories based on different colors, with the gray area within the circles representing different GC contents.

The complete mitochondrial genome of *D. officinale* exhibits a multibranched structure, spanning a total length of 639,931 bp with a GC content of 43.41%. It consists of 22 mitochondrial chromosomes, including 19 circular and 3 linear (Figure 1 and Figure 2). The features of each assembled chromosome in the *D. officinale* mitogenome are shown in Table S2. Genome annotation identified a total of 62 genes, comprising 35 protein-coding genes (PCGs), 3 ribosomal RNA (rRNA) genes, and 24 transfer RNA (tRNA) genes (Table 1). These PCGs can be classified into several categories based on their functions, which primarily include: ATP synthase (*atp1*, *atp4*, *atp6*, *atp8*, and *atp9*); NADH dehydrogenase (*nad1*, *nad2*, *nad3*, *nad4*, *nad4L*, *nad5*, *nad6*, *nad7*, and *nad9*); RNA maturase (*matR*); ribosomal proteins (*rpl5*, *rps1*, *rps10*, *rps11*, *rps12*, *rps13*, *rps14*, *rps2*, *rps4*, *rps7*); succinate dehydrogenase (*sdh4*); Cytochrome bc1 complex (*cob*); Cytochrome coxidase (*cox1*, *cox2*, *cox3*); Cytochrome c biogenesis (*ccmB*, *ccmC*, *ccmFC*, *ccmFN*); and Transport membrane protein (*mttB*).

### 3.2. Codon Usage Analysis

**Figure 3:**
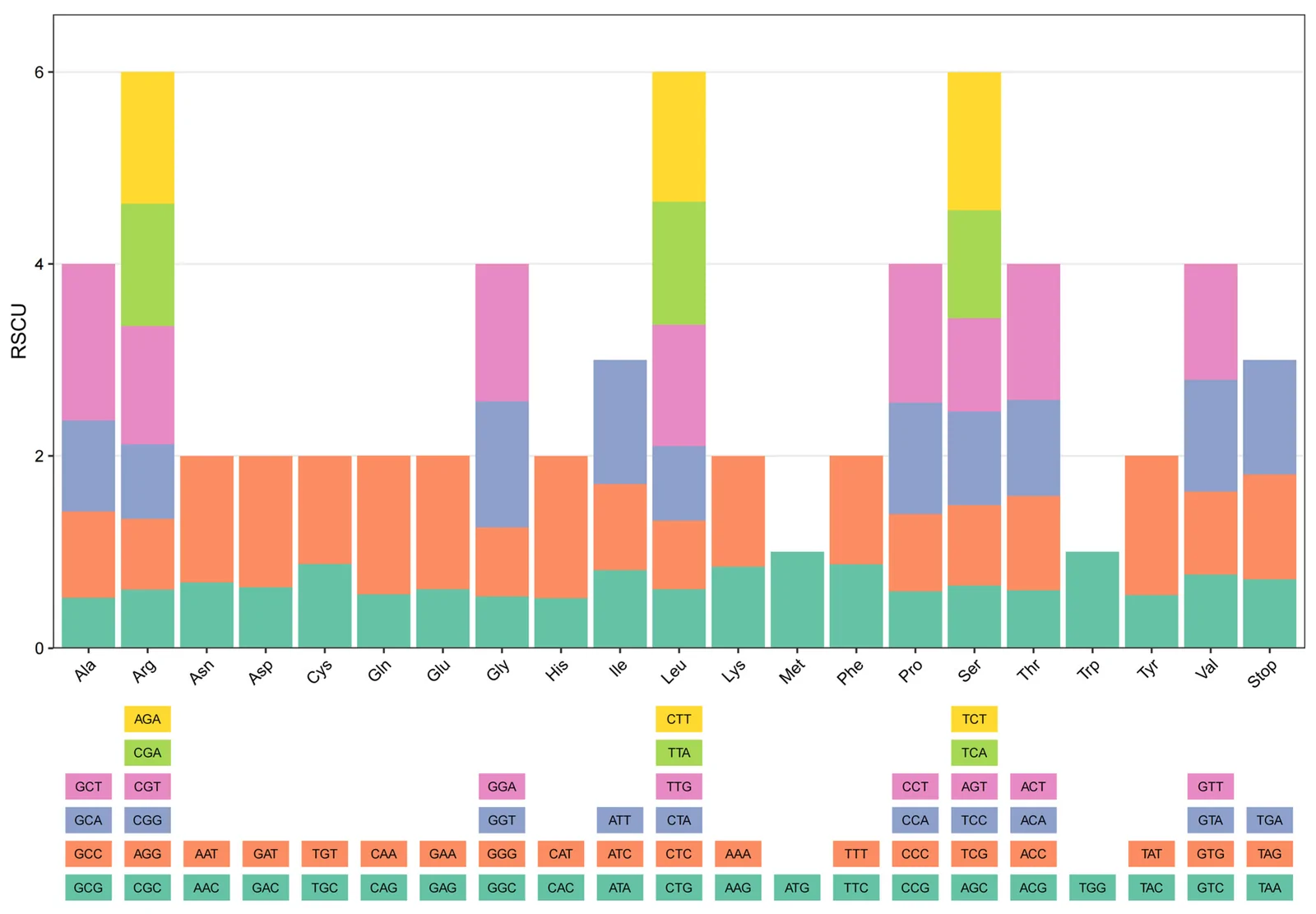
Relative synonymous codon usage (RSCU) analysis in the *D. officinale* mitochondrial genome.

Among the 35 PCGs identified in the complete mitochondrial genome of *D. officinale*, a total of 9600 codons were detected (Table 2). The mitochondrial DNA encodes 20 amino acids, represented by 61 different codons. The most frequent codon was TTT, which occurred 348 times. Each amino acid was encoded by at least one codon, with up to six codons per amino acid (Figure 3). In terms of frequency, leucine (Leu) was the most abundant amino acid, appearing 1009 times. In contrast, Cysteine (Cys) was the least frequent, with only 137 occurrences. Relative synonymous codon usage (RSCU) analysis revealed that 29 codons exhibited RSCU values greater than 1, of which 27 end with A/U. Meanwhile, the RSCU values of 33 codons were less than 1. It is worth noting that the codons ATG encoding methionine (Met) and TGG encoding tryptophan (Trp) usually do not exhibit usage bias (RSCU ≈ 1).

### 3.3. Repeat Sequence Analysis

**Figure 4:**
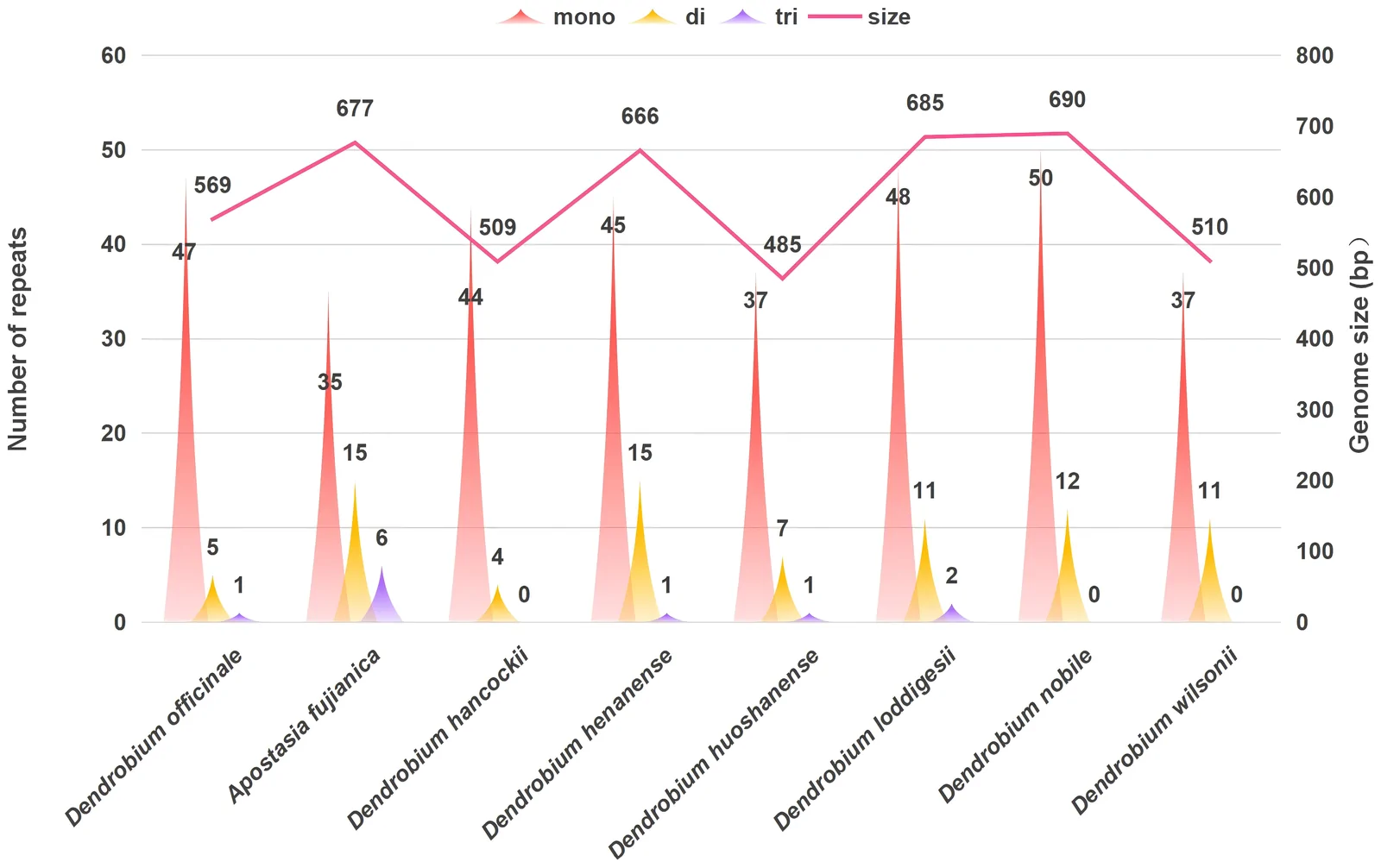
A comparison of the numbers of simple sequence repeats and the sizes of the mitochondrial genomes of eight Orchidaceae species. Note: The *X*-axis shows different species. Columns with different colors represent repeats with different types of repeat units. The number of a particular type of repeat is shown above the corresponding column. The sizes of the mitochondrial genomes are connected with a line.

Repetitive sequences play a critical role in the evolution of plant mitochondrial genomes. In the mitochondrial genome of *D. officinale*, 53 SSRs were identified, with a total length of 569 bp (Table S3). Three types of SSRs were detected, mononucleotide, dinucleotide, and trinucleotide repeats, which accounted for 88.68% (47), 9.43% (5), and 1.89% (1), respectively. Among all mononucleotide repeats, the A/T type is the most abundant. Furthermore, we compared the SSR distribution in *D. officinale* with that in seven other Orchidaceae species (*Apostasia fujianica*, *Dendrobium hancockii*, *Dendrobium henanense*, *Dendrobium huoshanense*, *Dendrobium loddigesii*, *Dendrobium nobile*, *Dendrobium wilsonii*) (Figure 4). The analysis revealed that *D. officinale* possesses fewer dinucleotide repeats compared with *D. nobile, D. loddigesii*, *D. henanense*, and *A. fujianica*. Trinucleotide repeats could be detected in *D. officinale*, whereas no trinucleotide SSRs were identified in *D. nobile*, *D. hancockii* and *D. wilsonii*. This feature serves as an important distinguishing marker for the SSR composition patterns among these species. Additionally, the mitochondrial genome SSR size of *D. officinale* is generally small, evidently smaller than those of *D. nobile*, *D. wilsonii*, *D. loddigesii*, *D. henanense* and *A. fujianica*.

**Figure 5:**
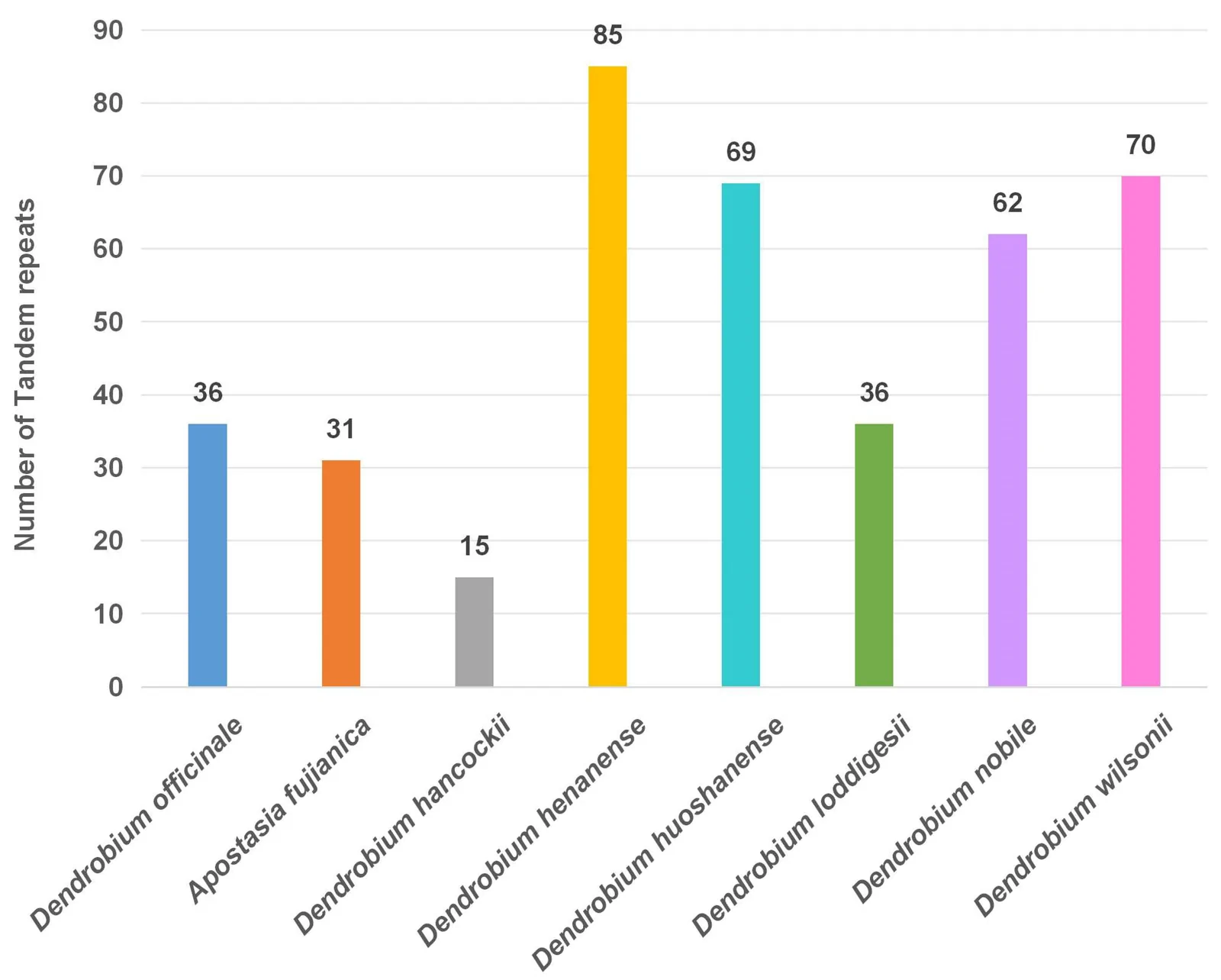
A comparison of the numbers of tandem repeats of the mitochondrial genomes of the eight Orchidaceae species.

A total of 36 tandem repeats were identified in the mitochondrial genome of *D. officinale*, with a combined total length of 1575 bp (Table S4). The abundance of tandem repeats varied greatly among eight Orchidaceae species. *D. henanense* had the highest number of tandem repeat sequences (85), followed by *D. wilsonii* (70) and *D. huoshanense* (69). *D. hancockii* had the lowest number of tandem repeat sequences, with only 15. The number of tandem repeats in *D. officinale* was equal to that in *D. loddigesii* but significantly lower than that in *D. henanense*, *D. huoshanense*, *D. nobile*, and *D. wilsonii* (Figure 5).

**Figure 6:**
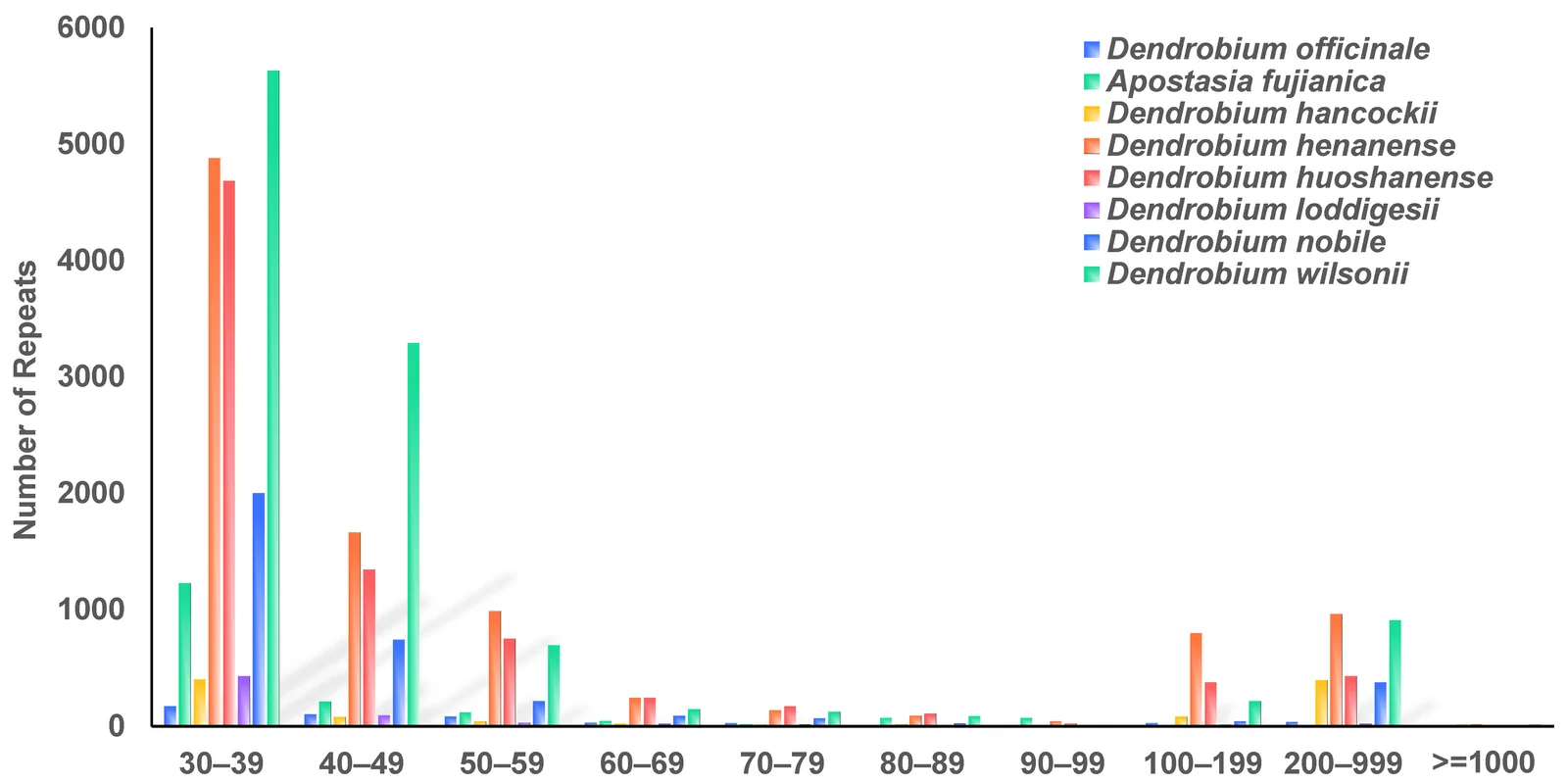
The length distribution of dispersed repeat sequences in the mitochondrial genomes of eight Orchidaceae species.

Furthermore, 511 dispersed repeats were identified, spanning 56,301 bp (Table S5). Among these, 462 were forward repeats, and 49 were palindromic repeats, with no reverse or complementary repeats observed. The lengths of these repetitive sequences primarily range from 30 to 2470 bp. The dispersed repeats in *D. officinale* are predominantly short fragments of 30–59 bp, with a smaller number of long dispersed repeats. Figure 6 illustrates the length distribution of dispersed repeat sequences in the mitochondrial genomes of eight Orchidaceae species. Overall, dispersed repeats in the 30–39 bp length range are the most abundant across all species, and the number of dispersed repeats in the 30–39 bp range in *D. wilsonii* is significantly higher than that in the other species.

### 3.4. Prediction of RNA Editing Sites

We examined the positions of RNA editing sites within different protein-coding genes of the *D. officinale* mitochondrial genome. A total of 522 RNA editing sites were identified in the mitochondrial genes, with the predominant editing type being C-to-U substitutions. These sites are unevenly distributed among different protein coding genes. Detailed information regarding specific sites, upstream and downstream sequences, and scores can be found in Table S6. Among all the genes analyzed, *nad4* possesses the highest number of editing sites (58), whereas *sdh4* has the fewest (2). By comparing the RNA editing sites of eight Orchidaceae species, we found that *rpl14*, *rpl16*, *rps14*, *rps19*, and *rps3* are absent in *D. officinale* (Table 3). It needs to be further clarified that *rpl14*, *rpl16*, *rps19*, and *rps3* were not annotated in the assembled *D. officinale* mitochondrial genome; *rps14* was excluded when compiling the RNA editing site results because its RNA editing prediction score was below 0.9. Interestingly, *D. loddigesii* also lacks these genes. In addition, compared to the other seven species, we found that *sdh4* only has editing sites in *D. officinale*. The number of predicted editing sites for the *atp1*, *cox3*, *nad4l*, and *rps12* genes is the same across all species. After RNA editing, 297 (56.90%) positions were changed from hydrophilic to hydrophobic, and 178 (34.10%) positions were changed from hydrophobic to hydrophilic. Furthermore, only eight (1.50%) positions of amino acids were changed from hydrophilic to stop (Table 4).

### 3.5. Homologous Sequence Analysis of Chloroplast and Mitochondria

**Figure 7:**
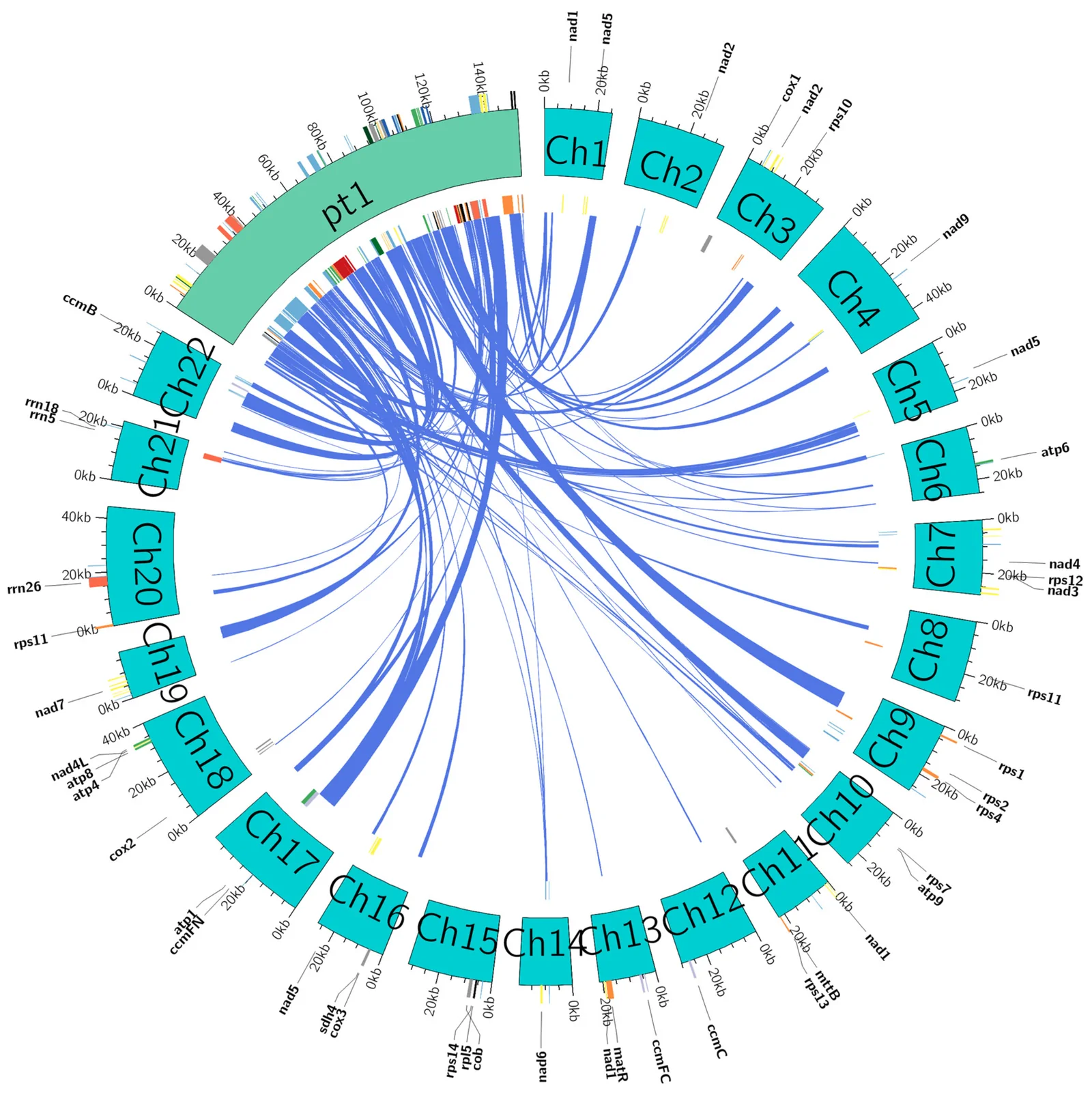
DNA and gene transfer between chloroplast and mitochondrial genomes in *D. officinale*. The blue arc represents mitochondrial genomes. The green arc represents the chloroplast genome. Homologous fragments are indicated using lines between the blue and green arcs. The genes marked on the image are located near the homologous region, which may have been transferred from chloroplasts. The genes annotated within the homologous region are shown in Table S7.

DNA transfer between organelles is a prominent feature of plant genome evolution. To investigate the chloroplast-derived DNA fragments (Mitochondrial Plastid DNAs, MTPTs) in the mitochondrial genome of *D. officinale*, we conducted a detailed analysis of the identified homologous fragments. A total of 125 MTPT fragments were identified (Figure 7; Table S7). The total length of these fragments is 108,012 bp, accounting for approximately 16.88% of the entire mitochondrial genome (639,931 bp). The lengths of these MTPT fragments range from 34 bp to 4735 bp, with sequence similarity ranging from 74.18% to 100.0%. By annotating the corresponding regions of these fragments on the chloroplast genome, we discovered that a significant number of chloroplast gene-related sequences had been transferred, either completely or partially, to the mitochondria. Statistical analysis demonstrated that these MTPT fragments collectively encompass 78 distinct annotated chloroplast genes. Of these, protein-coding genes represent the category with the largest number of transfer events, with a total of 57 transferred protein-coding genes. Specifically, 18 tRNA genes, 3 rRNA genes, and 57 protein-coding genes were identified as having been transferred. These results indicate that large-scale and frequent DNA transfer events from chloroplasts to mitochondria have occurred during the evolution of *D. officinale*.

### 3.6. Phylogenetic Analysis

**Figure 8:**
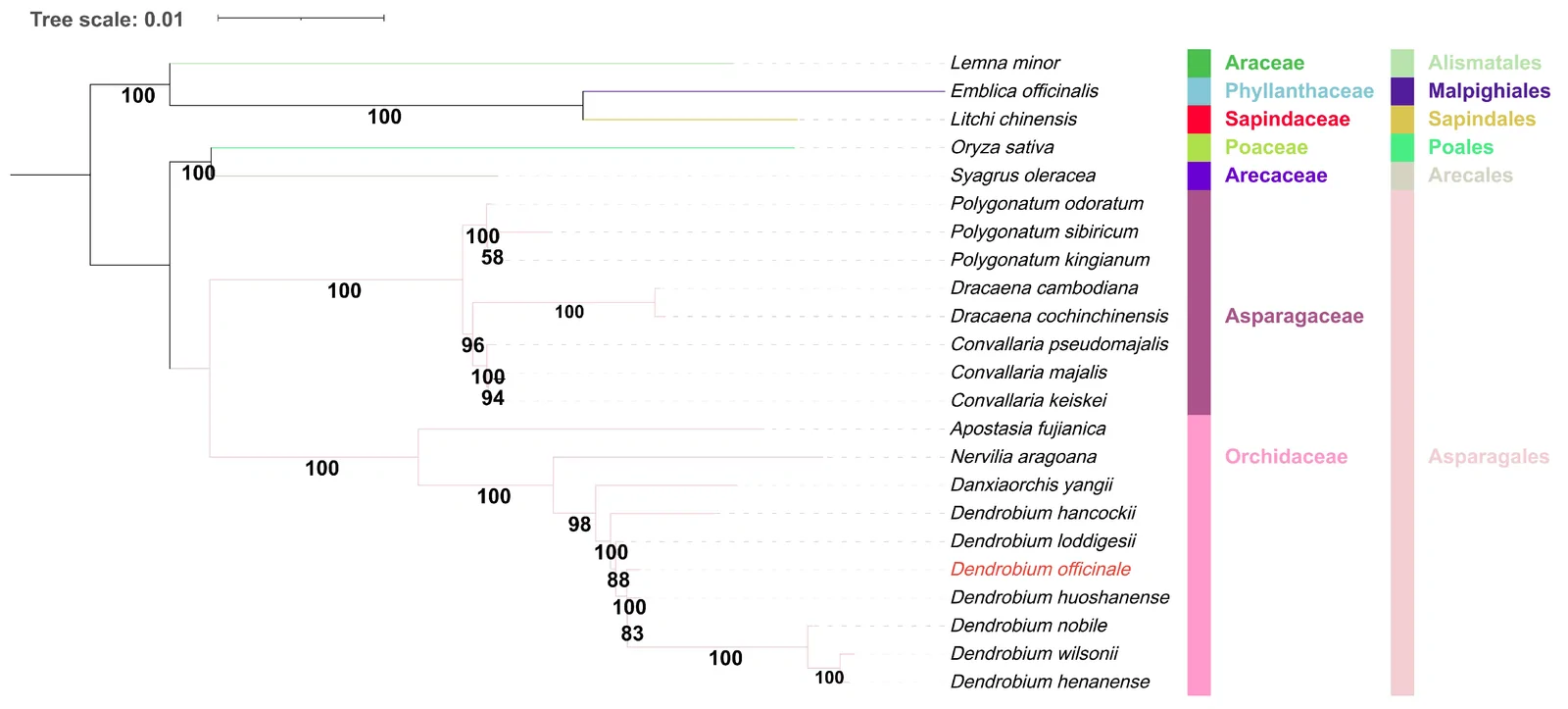
The phylogenetic relationships of *D. officinale* with other species based on the maximum likelihood tree.

To clarify the phylogenetic position of *D. officinale*, 38 core protein-coding genes (*atp1*, *atp4*, *atp6*, *atp8*, *atp9*, *ccmb*, *ccmc*, *ccmfc*, *ccmfn*, *cob*, *cox1*, *cox2*, *cox3*, *matr*, *mttb*, *nad1*, *nad2*, *nad3*, *nad4*, *nad4l*, *nad5*, *nad6*, *nad7*, *nad9*, *rpl2*, *rpl5*, *rpl16*, *rps1*, *rps2*, *rps3*, *rps4*, *rps7*, *rps10*, *rps11*, *rps12*, *rps13*, *rps14*, *rps19*) distributed across 23 species were retrieved for phylogenetic analysis. The topology of the maximum likelihood phylogenetic tree revealed that *D. officinale* is clustered with *D. henanense*, *D. wilsonii*, *D. nobile*, and *D. huoshanense* to form a single monophyletic clade (Figure 8). This clade obtained 100% bootstrap support, suggesting that the topological structure is highly robust based on the current dataset. This topological result is consistent with the phylogenetic trees constructed via maximum parsimony and Bayesian inference (Figures S1 and S2). The overall topological structure of the phylogenetic tree is highly consistent with the current taxonomic classification of the included species, and all examined species are clearly clustered into their corresponding taxonomic families.

### 3.7. Collinear Analysis

**Figure 9:**
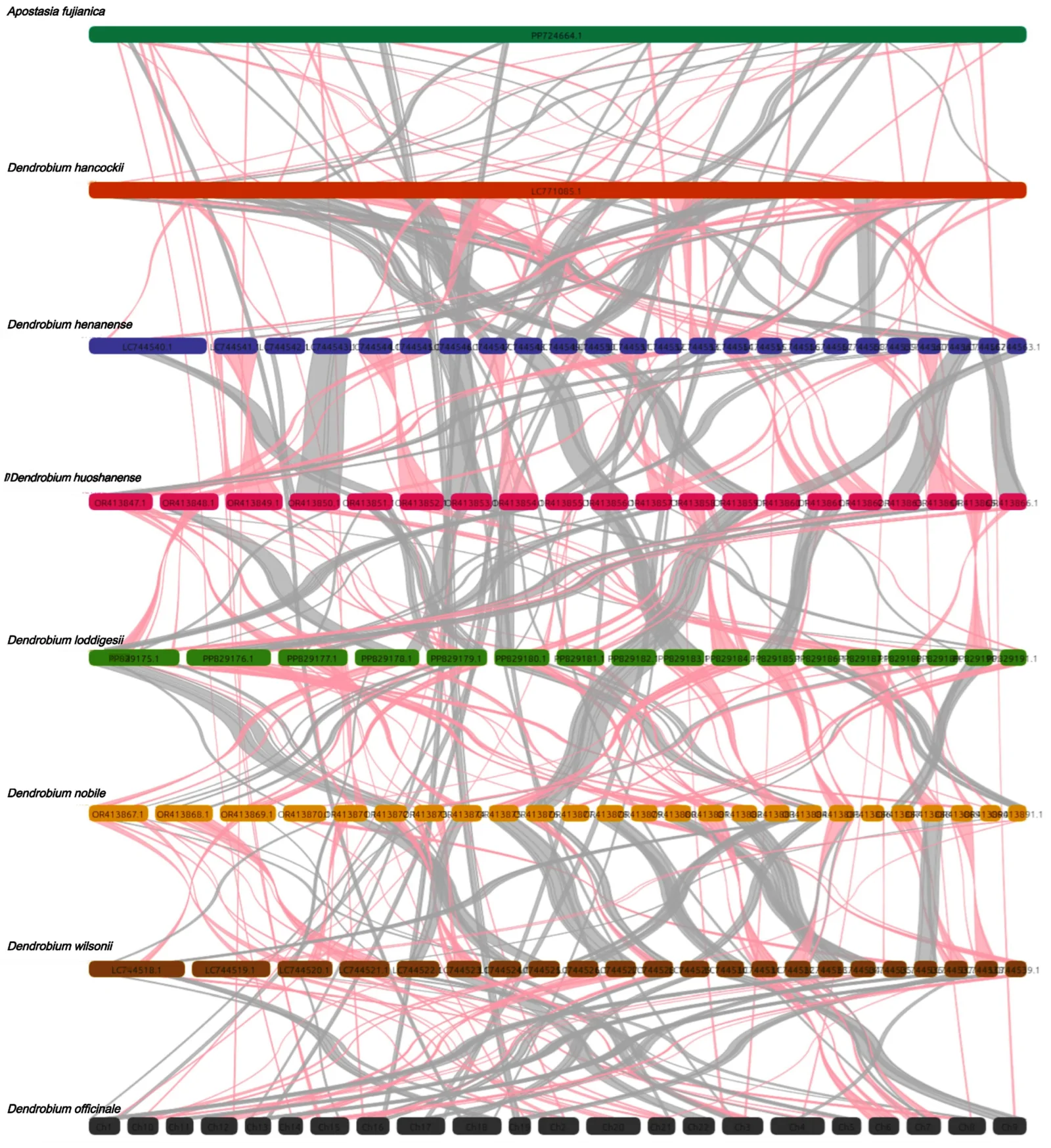
A collinear analysis of eight Orchidaceae species. The red arcs indicate inverted regions, while the gray arcs indicate better homologous regions. The red arcs indicated inverted regions. The gray arcs indicated better homologous regions.

We conducted a collinearity comparative analysis between *D. officinale* and seven closely related species (*A. fujianica*, *D. hancockii*, *D. henanense*, *D. huoshanense*, *D. loddigesii*, *D. nobile*, and *D. wilsonii*). Among all the compared species, *D. officinale* and *D. nobile* exhibited the highest homology. The shared homologous region between the two covered 80.87% of the mt genome (Table S8). This high coverage rate indicates that the mitochondrial genome we assembled has a high degree of completeness and is closely related to *D. nobile*. Despite the close phylogenetic relationships, the differences in the order of block arrangement observed in the collinearity map (Figure 9) collectively confirm the structural rearrangement events in plant mitochondrial genomes during evolution, exhibiting a high degree of structural non-conservation.

### 3.8. Ka/Ks Analysis

**Figure 10:**
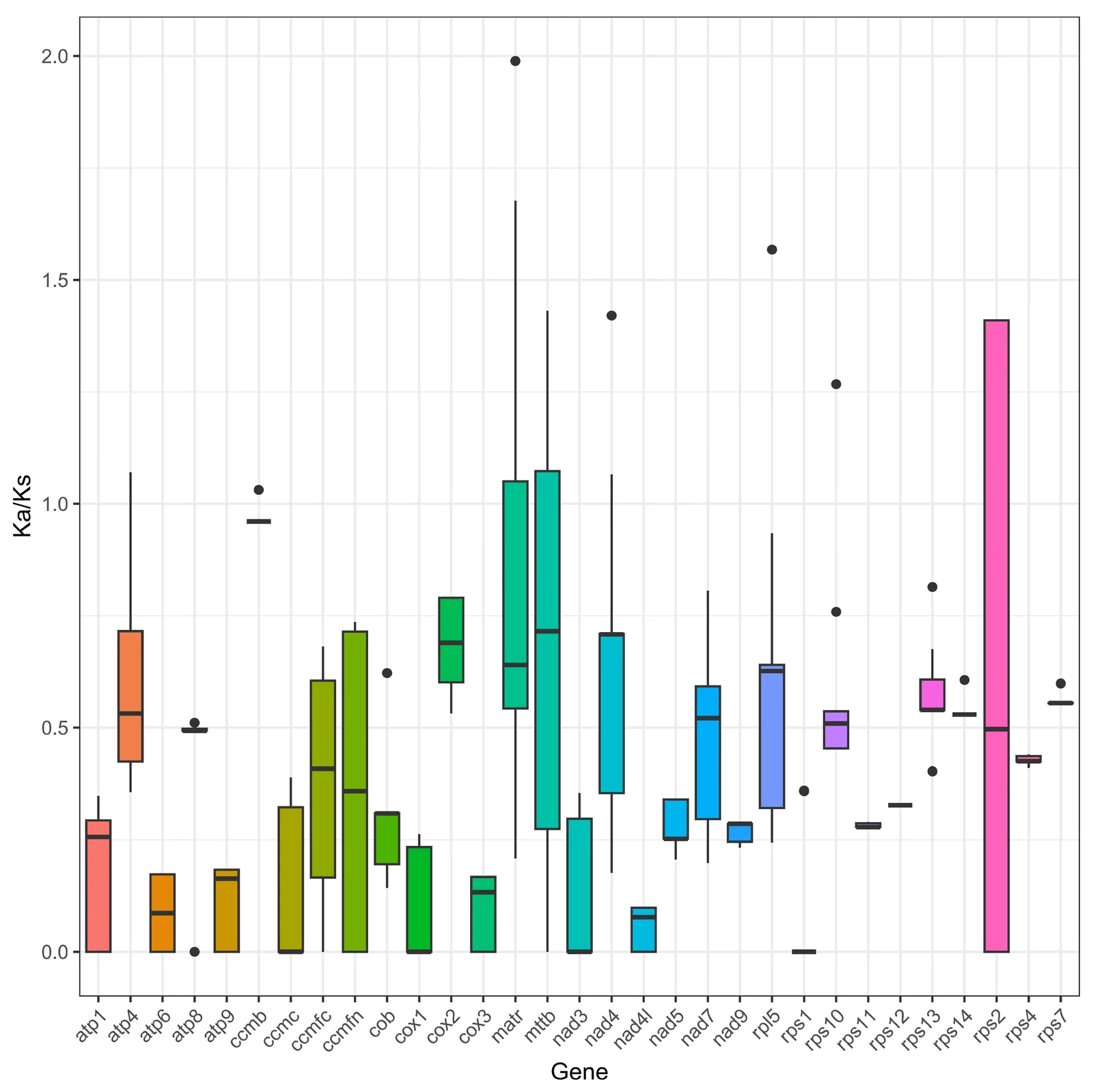
The Ka/Ks ratio calculated for the 31 shared genes from the mitochondrial genome.

In this study, we analyzed the Ka/Ks values of 31 shared mitochondrial genes across eight plant species to elucidate their evolutionary selection patterns (Figure 10). Overall, most genes exhibit strong evolutionary constraints. Based on the distribution of Ka/Ks values, a total of 19 genes have a median Ka/Ks ratio significantly less than 1, indicating that they are subject to strong purifying selection and exhibit highly conserved functions. These genes (such as *ccmc*, *rps1*, *nad3*, *cox1*) primarily encode key subunits of core energy metabolism pathways such as respiratory chain complexes and ATP synthase. The stability of their sequences is crucial for maintaining the basic functions of mitochondria. The Ka/Ks values of another 12 genes (such as *atp4*, *ccmb*, *cox2*, *matr*) are close to the neutral threshold (0.5 ≤ ω < 1) (Table S9). This suggests that they may be in a dynamic process transitioning from purifying selection to adaptive evolution or may be under weaker selective pressure. This pattern of selective pressure differentiation provides crucial clues for understanding the functional evolution of different mitochondrial genes during species adaptation.

## 4. Discussion

### 4.1. The Structural Diversity of the D. officinale Mitochondrial Genome

Mitochondria are essential organelles in eukaryotic cells, providing the energy necessary for various physiological processes [28]. Plant mitogenomes have become important tools for species identification, phylogenetic analysis, and inheritance patterns. Traditionally, plant mitochondrial genomes are described as circular, with many species mapped to a single “master circle” chromosome [29]. However, recent studies have revealed that plant mitogenomes exhibit remarkable structural diversity, including linear, circular, branched, and network conformations [30]. The mitochondrial genome of *D. officinale* adopts a complex multibranched configuration composed of both circular and linear chromosomes, a conformation that differs from that reported for four Dendrobium species [31]. This discrepancy is unlikely to be purely technical, because the two studies examined the same species; rather, it points to the possibility that multipartite mitochondrial architectures are not fixed within a species and may vary among populations or be resolved differently depending on assembly strategy. In angiosperms, mitochondrial genomes are highly dynamic, shaped by recombination, repetitive sequences, and gene transfer, which together contribute to variations in structure and size [32,33]. The range of sizes documented across angiosperms, from the extremely compact mitogenome of *Viscum scurruloideum* (66 kb) to the exceptionally large one of *Silene conica* (11.3 Mb), underscores the extraordinary evolutionary flexibility of plant mitochondrial genomes [34].

Previous studies have reported that the mitochondrial genome of *D. officinale* contains 64 genes (containing five repetitive gene fragments); however, comparative analysis revealed that our annotation lacks *rpl16*, *rps3*, and *rps19.* In contrast, the genes added to the annotation are *sdh4*, *trnG-GCC*, *trnG-UCC*, *trnL-UAA*, *trnS-GGA*, and *trnV-UAC*. This apparent discrepancy in gene content not only likely reflects differences in sequencing depth, assembly strategies, and annotation criteria but also suggests the presence of genuine mitochondrial structural variation among different *D. officinale* populations [35]. In particular, genes of the *rps* family have been repeatedly reported to undergo loss, intracellular transfer, or functional degeneration in Orchidaceae and other angiosperms [36], indicating that these genes are evolutionarily labile and more prone to lineage-specific or population-level elimination during mitochondrial genome evolution. Compared with the previous research results, in this study, the absence of *rps3* and *rps19*, together with the retention of other *rps* genes, is consistent with this pattern of genomic plasticity [31]. Such variability may be attributed to intracellular gene transfer to the nucleus, pseudogenization or sequence divergence that escapes annotation, substoichiometric isoforms generated by recombination, or genuine intraspecific variation among populations. Distinguishing among these possibilities will require population-level sampling and experimental validation, which our current dataset cannot provide.

### 4.2. Patterns of Codon Use Bias and Repetitive Sequences

Codons are fundamental carriers of genetic information in nature [37]. During protein translation, among the synonymous codons encoding the same amino acid, certain codons are used at significantly higher frequencies than others. This evolutionary-conserved feature is defined as codon usage bias [38]. This bias plays a significant role in genetic processes and variation [39]. We identified 29 codons with a higher-than-expected usage (RSCU > 1) and 33 codons with a lower-than-expected usage (RSCU < 1), suggesting that codon bias may be influenced by natural selection. These preferences are likely associated with translation efficiency and the accuracy of protein synthesis [40].

Repetitive sequences play a critical role in the evolution of plant mitochondrial genomes [41]. SSRs are widely utilized as molecular markers for species identification and genetic diversity studies [42]. In *D. officinale*, SSRs were dominated by mononucleotide repeats, underscoring their potential as markers for genetic diversity and species differentiation within the genus. Additionally, tandem and dispersed repeats were also identified and may facilitate genome rearrangement and structural variation. These findings are broadly consistent with those of Wang et al. (2019), who reported that repetitive sequences significantly contribute to the evolution of plant mitochondrial genomes [43]. In comparison, Wang et al. (2024) identified significantly higher numbers of SSRs, tandem repeats and dispersed repeats in four *Dendrobium* mitogenomes, suggesting stronger recombination activity and repeat-driven expansion in certain species [31]. The relatively moderate repeat abundance observed in our assembly may reflect species-specific genomic stability or differences in sequencing resolution. At the same time, repeat identification is highly dependent on the detection tools and thresholds employed, and differences in the minimum repeat length, mismatch tolerance, and the repeat library used for annotation can directly affect the number of repeats recovered. Accordingly, cross-study comparisons of repeat abundance should be interpreted with caution unless the same pipeline is applied.

### 4.3. RNA Editing Events and Selection Pressure

In plants, RNA editing primarily occurs in organelles, including mitochondria and chloroplasts, and plays a crucial role in their specific functions. RNA editing represents a crucial post-transcriptional mechanism in plant organelles, essential for the production of functional proteins through the alteration in RNA nucleotide sequences. In the mitogenome of *D. officinale*, RNA editing sites were identified predominantly as cytosine-to-uridine transitions, which are characteristic of plant mitochondrial genomes [44]. These modifications likely guarantee the precise translation of mitochondrial genes and the proper functioning of mitochondria. Previous studies reported that higher RNA editing frequencies, 510 sites in *D. officinale*, highlighting possible methodological differences or biological variation across samples. These RNA editing events in specific genes might be functionally significant for their corresponding protein products or essential for their proper assembly into functional complexes involved in mitochondrial electron transport and energy production [31]. We found that *sdh4* only has editing sites in *D. officinale*, whereas homologous genes in other closely related plant species exhibit a lack, implying that this lineage-specific RNA editing event may contribute to the functional regulation of the succinate dehydrogenase complex in *D. officinale* mitochondria. While the present findings provide a valuable framework for understanding RNA editing in *D. officinale*, the editing sites reported here were derived from bioinformatic prediction, and their in vivo editing rates remain to be experimentally confirmed. Future validation using RT-PCR, qPCR, or RNA-seq, together with the functional characterization of the *sdh4* editing event, will help clarify its adaptive significance in the growth and development of *D. officinale*.

By incorporating a broad taxonomic sampling in the Ka/Ks analysis, this study captures a wider spectrum of evolutionary contexts. Most genes, such as *ccmC*, *rps1*, *nad3*, and *cox1*, exhibited strong purifying selection, reflecting their essential roles in maintaining mitochondrial energy metabolism [45]. However, several genes, including *atp4*, *ccmb*, *cox2*, and *matR*, have Ka/Ks values close to the neutral threshold (0.5 ≤ ω < 1). This indicates that they are relatively less subject to purifying selection pressure, or there are significant rate differences between different comparative combinations. Notably, the inclusion of diverse species allows these lineage-specific selective pressures to be detected more effectively, highlighting evolutionary heterogeneity among mitochondrial genes [46]. Overall, the extensive species selection employed in this analysis strengthens the robustness of the evolutionary inference by revealing both conserved constraints and divergent adaptive trends, underscoring the utility of Ka/Ks analysis as a robust tool for studying gene evolutionary dynamics [47].

### 4.4. Phylogenetic and Evolutionary Analyses

Frequent sequence transfer occurs between organellar genomes, including plastid-to-mitochondria and nucleus-to-mitochondria events [48]. In this study, sequence similarity analysis revealed 125 homologous fragments, totaling 108,012 bp, shared between the mitochondrial and chloroplast genomes of *D. officinale.* This finding suggests potential gene transfer or functional co-evolution between the two organelles. Phylogenetic analysis based on protein-coding genes demonstrated that *D. officinale* is clustered with *D. henanense*, *D. wilsonii*, *D. nobile*, and *D. huoshanense* to form a single monophyletic clade. This result reinforces the established relationships within Orchidaceae [49,50]. Furthermore, collinearity analysis indicated that closely related species tend to retain longer collinear blocks, providing valuable genomic evidence for evolutionary studies in Orchidaceae [51].

The mitogenome assembled here expands the genomic resources available for *D. officinale*, a species of considerable medicinal and economic value, and provides a basis for developing mitochondrial markers for species authentication and genetic diversity assessment. Several limitations should nevertheless be acknowledged. The mitochondrial genome was assembled from a single individual, so intraspecific structural variation cannot be assessed, and the gene content differences relative to previous reports should be interpreted with caution. All RNA editing sites were predicted computationally, and their in vivo editing rates remain unverified. Finally, the phylogenetic inferences are based on a limited number of mitochondrial genes and should be interpreted alongside plastid and nuclear evidence. Addressing these limitations through multi-individual sampling, the experimental validation of editing sites, and integrative organellar analyses will be important directions for future work.

## 5. Conclusions

This study characterizes the mitochondrial genome of *D. officinale*, revealing a complex multibranched architecture, abundant repetitive sequences, numerous RNA editing sites, and substantial MTPTs. Ka/Ks analysis showed that most mitochondrial genes are under strong purifying selection, whereas the repetitive elements, editing sites, and MTPTs reflect considerable plasticity that may underlie population-level adaptation. However, gene content differences relative to previous reports cannot be confidently attributed to population-level structural variation; the predicted RNA editing sites, including the lineage-specific *sdh4* event, remain experimentally unverified; and phylogenetic inferences rest on a limited set of mitochondrial genes. Future research should further extend sampling to multiple populations; validate editing sites using RT-PCR, qPCR, or RNA-seq; and integrate mitochondrial, plastid, and nuclear data. Such efforts will refine evolutionary inferences.

## Figures and Tables

**Table 1:** The annotation results of *D. officinale*.

Group of Genes	Name of Genes
ATP synthase	*atp1*, *atp4*, *atp6*, *atp8*, *atp9*
Cytochrome c biogenesis	*ccmB*, *ccmC*, *ccmFC*, *ccmFN*
Cytochrome b	*cob*
Cytochrome c oxidase	*cox1*, *cox2*, *cox3*
Transport membrane protein	*mttB*
NADH dehydrogenase	*nad1*, *nad2*, *nad3*, *nad4*, *nad4L*, *nad5*, *nad6*, *nad7*, *nad9*
RNA maturase	*matR*
Ribosomal proteins	*rpl5*, *rps1*, *rps10*, *rps11*, *rps12*, *rps13*, *rps14*, *rps2*, *rps4*, *rps7*
Succinate dehydrogenase	*sdh4*
Ribosome RNA	*rrn18*, *rrn26*, *rrn5*
Transfer RNA	*trnC-GCA*, *trnD-GUC*, *trnE-UUC*, *trnF-GAA*, *trnG-GCC*, *trnG-UCC*, *trnH-GUG*, *trnI-CAU*, *trnK-UUU*, *trnL-UAA*, *trnL-UAG*, *trnM-CAU*, *trnN-GUU*, *trnP-UGG*, *trnQ-UUG*, *trnR-UCU*, *trnS-GCU*, *trnS-GGA*, *trnT-UGU*, *trnV-GAC*, *trnV-UAC*, *trnW-CCA*, *trnY-GUA*, *trnfM-CAU*

**Table 2:** Relative synonymous codon usage for each amino acid in the mitochondrial genome of *D. officinale*.

Amino Acid	Codon	Count	RSCU	Amino Acid	Codon	Count	RSCU
Ala	GCG	75	0.523	Leu	CTA	131	0.779
Ala	GCC	129	0.899	Leu	TTG	212	1.261
Ala	GCA	136	0.948	Leu	TTA	216	1.284
Ala	GCT	234	1.631	Leu	CTT	227	1.35
Arg	CGC	69	0.607	Lys	AAG	158	0.847
Arg	AGG	84	0.739	Lys	AAA	215	1.153
Arg	CGG	88	0.774	Met	ATG	265	1
Arg	CGT	140	1.232	Phe	TTC	268	0.87
Arg	CGA	145	1.276	Phe	TTT	348	1.13
Arg	AGA	156	1.372	Pro	CCG	82	0.59
Asn	AAC	99	0.683	Pro	CCC	112	0.806
Asn	AAT	191	1.317	Pro	CCA	161	1.158
Asp	GAC	104	0.634	Pro	CCT	201	1.446
Asp	GAT	224	1.366	Ser	AGC	102	0.649
Cys	TGC	60	0.876	Ser	TCG	132	0.84
Cys	TGT	77	1.124	Ser	TCC	153	0.973
Gln	CAG	79	0.556	Ser	AGT	153	0.973
Gln	CAA	205	1.444	Ser	TCA	177	1.126
Glu	GAG	115	0.61	Ser	TCT	226	1.438
Glu	GAA	262	1.39	Thr	ACG	74	0.598
Gly	GGC	86	0.535	Thr	ACC	122	0.986
Gly	GGG	116	0.722	Thr	ACA	124	1.002
Gly	GGT	211	1.313	Thr	ACT	175	1.414
Gly	GGA	230	1.431	Trp	TGG	147	1
His	CAC	70	0.519	Tyr	TAC	77	0.55
His	CAT	200	1.481	Tyr	TAT	203	1.45
Ile	ATA	196	0.809	Val	GTC	115	0.762
Ile	ATC	218	0.9	Val	GTG	131	0.868
Ile	ATT	313	1.292	Val	GTA	176	1.166
Leu	CTG	103	0.612	Val	GTT	182	1.205
Leu	CTC	120	0.714				

**Table 3:** The prediction of RNA editing sites in the eight Orchidaceae species.

Gene	*Dendrobium officinale*	*Apostasia fujianica*	*Dendrobium hancockii*	*Dendrobium henanense*	*Dendrobium huoshanense*	*Dendrobium loddigesii*	*Dendrobium nobile*	*Dendrobium wilsonii*
*atp1*	11	11	11	11	11	11	11	11
*atp4*	10	11	10	10	10	10	10	10
*atp6*	23	0	24	4	23	24	4	4
*atp8*	3	4	3	3	3	3	3	4
*atp9*	8	1	8	8	9	8	9	8
*ccmb*	32	0	30	32	32	32	32	32
*ccmc*	31	33	33	31	31	32	31	31
*ccmfc*	7	17	13	13	13	14	14	14
*ccmfn*	31	32	42	31	31	31	31	31
*cob*	16	16	16	15	15	0	16	16
*cox1*	28	26	28	28	28	28	28	28
*cox2*	15	0	11	17	16	15	15	16
*cox3*	13	13	13	13	13	13	13	13
*matr*	12	13	12	12	12	0	12	12
*mttb*	29	0	28	28	28	8	28	0
*nad1*	29	0	5	0	0	29	29	0
*nad2*	12	0	19	0	31	32	32	0
*nad3*	20	18	20	20	20	20	20	20
*nad4*	58	0	56	37	57	58	57	38
*nad4l*	11	11	11	11	11	11	11	11
*nad5*	8	26	25	0	33	33	33	0
*nad6*	16	14	16	16	16	16	16	16
*nad7*	32	37	31	32	34	35	34	32
*nad9*	14	13	14	13	13	14	13	13
*rpl14*	0	0	1	0	0	0	0	0
*rpl16*	0	13	11	11	11	0	11	11
*rpl5*	9	9	9	9	9	8	9	9
*rps1*	7	0	7	7	7	0	7	6
*rps10*	2	1	2	2	2	2	2	2
*rps11*	2	3	0	1	2	0	2	0
*rps12*	6	6	6	6	6	6	6	6
*rps13*	5	4	5	5	5	5	5	5
*rps14*	0	1	0	0	0	0	0	0
*rps19*	0	6	6	6	6	0	6	6
*rps2*	4	0	4	4	4	0	4	4
*rps3*	0	12	15	15	15	0	14	15
*rps4*	14	15	14	14	14	0	14	14
*rps7*	2	1	2	2	2	2	2	2
*sdh4*	2	0	0	0	0	0	0	0

**Table 4:** The prediction of RNA editing sites in the *D. officinale* mitochondrial genome.

Type	RNA Editing	Number	Percentage
Hydrophilic–hydrophobic	ACA (T) => ATA (I)	3	56.90%
	ACC (T) => ATC (I)	2	
	ACG (T) => ATG (M)	3	
	ACT (T) => ATT (I)	1	
	CAC (H) => TAC (Y)	10	
	CAT (H) => TAT (Y)	17	
	CGC (R) => TGC (C)	10	
	CGG (R) => TGG (W)	35	
	CGT (R) => TGT (C)	23	
	TCA (S) => TTA (L)	69	
	TCC (S) => TTC (F)	39	
	TCG (S) => TTG (L)	40	
	TCT (S) => TTT (F)	45	
Hydrophobic–hydrophilic	CCA (P) => TCA (S)	54	34.10%
	CCC (P) => TCC (S)	30	
	CCG (P) => TCG (S)	41	
	CCT (P) => TCT (S)	53	
Hydrophobic	ATC (I) => ATT (I)	3	7.30%
	CTA (L) => TTA (L)	1	
	CTC (L) => TTC (F)	8	
	CTG (L) => TTG (L)	3	
	CTT (L) => TTT (F)	6	
	GCA (A) => GTA (V)	1	
	GCC (A) => GTC (V)	1	
	GCG (A) => GTG (V)	2	
	GTC (V) => GTT (V)	2	
	TAC (Y) => TAT (Y)	1	
	TTC (F) => TTT (F)	10	
Hydrophilic–stop	CAA (Q) => TAA (X)	3	1.50%
	CAG (Q) => TAG (X)	2	
	CGA (R) => TGA (X)	3	
Hydrophilic	AAC (N) => AAT (N)	1	0.20%

## Data Availability

The data that support the findings of this study are freely available in GenBank of NCBI (https://www.ncbi.nlm.nih.gov/), with accession numbers PZ861981-PZ862002.

## Supplementary Materials

The following supporting information can be downloaded at https://www.mdpi.com/article/10.3390/biology15191763/s1, Figure S1: The phylogenetic relationships of *D. officinale* with other species by the Maximum parsimony tree. Figure S2: The phylogenetic relationships of *D. officinale* with other species by the Bayesian inference tree. Table S1: Species and GenBank accessions used for phylogenetic analysis. Table S2: The features of each assembled chromosome in *D. officinale* mitogenome. Table S3: SSRs in the mitochondrial genome of *D. officinale.* Table S4: Tandem repeat sequences in the mitochondrial genome of *D. officinale.* Table S5: Dispersed repeat sequences in the mitochondrial genome of *D. officinale.* Table S6: RNA editing sites of protein-coding genes in mitochondrial genomes of *D. officinale.* Table S7: The identification MTPTs in *D. officinale* mitogenomes. Table S8: Results of collinearity analysis between *D. officinale* and seven other Orchidaceae species. Table S9: Median Ka/Ks values and selection category for 31 shared mitochondrial genes.

## Abbreviations

The following abbreviations are used in this manuscript:

PCGs	protein-coding genes
SSRs	simple sequence repeats
RSCU	relative synonymous codon usage
