# Supplementary material for: The Structure, Evolution, and Comparative Genomics of the Dendrobium officinale Mitochondrial Genome
Source: Biology (Basel). 2026 Oct 3;15(19):1763. doi: 10.3390/biology15191763 (PMC13664428; doi:10.3390/biology15191763)
Supplement: Supplementary file 1 [file biology-15-01763-s001.zip › biology-4551595-supplementary.pdf]

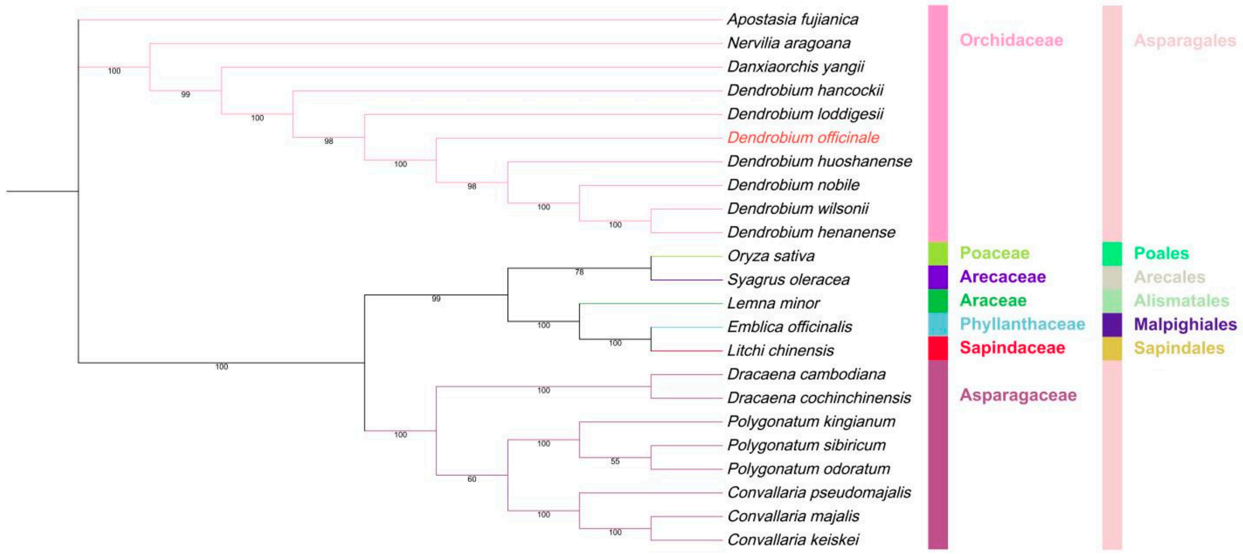

Figure S1. The phylogenetic relationships of *D. officinale* with other species by the Maximum parsimony tree.

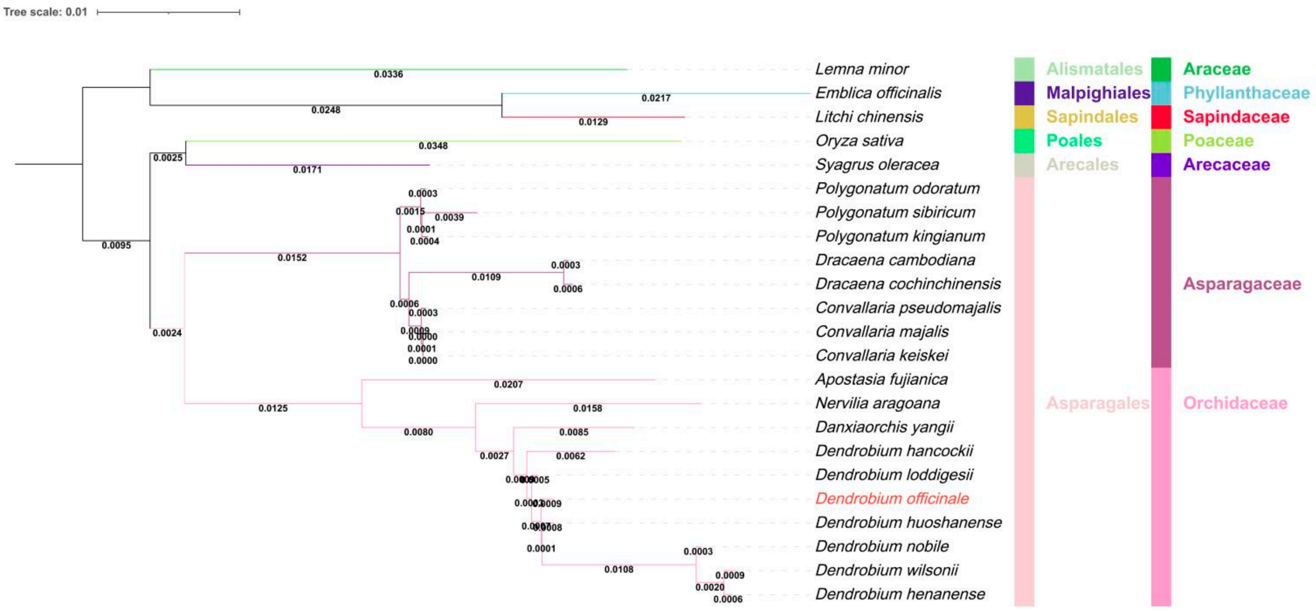

Figure S2. The phylogenetic relationships of *D. officinale* with other species by the Bayesian inference tree.

Table S1. Species and GenBank accessions used for phylogenetic anlaysis.

No.	Species	Accession numbers
1	*Dendrobium nobile*	OR413867-OR413891
2	*Dendrobium wilsonii*	LC744518-LC744539
3	*Dendrobium henanense*	LC744540-LC744563
4	*Dendrobium huoshanense*	OR413847-OR413866
5	*Dendrobium loddigesii*	PP829175-PP829191
6	*Dendrobium hancockii*	LC771085
7	*Apostasia fujianica*	PP724664
8	*Nervilia aragoana*	OP431986
9	*Dracaena cochinchinensis*	PP704687
10	*Convallaria keiskei*	PQ014258
11	*Convallaria majalis*	PQ014259
12	*Convallaria pseudomajalis*	PQ014260
13	*Polygonatum odoratum*	PQ154595
14	*Lemna minor*	PQ156039
15	*Dracaena cambodiana*	PQ279227
16	*Danxiaorchis yangii*	PQ306624
17	*Polygonatum kingianum*	PQ365714
18	*Polygonatum sibiricum*	PQ932596
19	*Syagrus oleracea*	PV763026
20	*Litchi chinensis*	PX659692
21	*Emblica officinalis*	PX733147
22	*Oryza sativa*	PX733954

Table S2. The features of each assembled chromosome in *D. officinale* mitogenome.

Genome	Feature	A %	C %	G %	T %	GC %	Size (bp)	Proportion in Genome (%)	Mean depth
Chr1	Whole genome	28.8	21.6	21.2	28.4	42.8	25338	100	50.0X
	Protein-coding genes	35.7	19.5	19.7	25.1	39.2	933	3.7	
	cis-spliced introns	-	-	-	-	-	-	-	
	tRNA genes	-	-	-	-	-	-	-	
	rRNA genes	-	-	-	-	-	-	-s	
	Non-coding regions	28.5	21.7	21.2	28.6	42.9	24405	96.3	
Chr2	Whole genome	29.2	21.8	21.5	27.5	43.3	33355	100	48.0X
	Protein-coding genes	38.6	17.1	21	23.4	38	539	1.6	
	cis-spliced introns	-	-	-	-	-	-	-	
	tRNA genes	30.6	23.6	19.4	26.4	43.1	72	0.2	
	rRNA genes	-	-	-	-	-	-	-	
	Non-coding regions	29	21.9	21.5	27.5	43.4	32744	98.2	
Chr3	Whole genome	28.2	22.2	21.9	27.6	44.1	33599	100	107.0X
	Protein-coding genes	29.2	22	21.1	27.8	43	2881	8.6	
	cis-spliced introns	-	-	-	-	-	-	-	
	tRNA genes	24.1	21.7	28.9	25.3	50.6	83	0.2	
	rRNA genes	-	-	-	-	-	-	-	
	Non-coding regions	28.2	22.2	22	27.6	44.2	30635	91.2	
Chr4	Whole genome	30.1	21.3	20.7	28	41.9	44347	100	131.0X
	Protein-coding genes	28.6	21.5	20.8	29.1	42.2	573	1.3	
	cis-spliced introns	-	-	-	-	-	-	-	
	tRNA genes	24.2	24.2	25.1	26.5	49.3	219	0.5	
	rRNA genes	-	-	-	-	-	-	-	
	Non-coding regions	30.1	21.3	20.6	28	41.9	43555	98.2	
Chr5	Whole genome	29.2	21.3	20.4	29	41.8	23806	100	49.0X
	Protein-coding genes	40.9	31.8	0	27.3	31.8	22	0.1	
	cis-spliced introns	-	-	-	-	-	-	-	
	tRNA genes	22.5	22.5	32.5	22.5	55	80	0.3	
	rRNA genes	-	-	-	-	-	-	-	
	Non-coding regions	29.3	21.3	20.4	29	41.7	23704	99.6	
Chr6	Whole genome	27.9	21.6	22.3	28.2	43.9	25024	100	48.0X

Genome	Feature	A %	C %	G %	T %	GC %	Size (bp)	Proportion in Genome (%)	Mean depth
	Protein-coding genes	24.1	21.1	18.8	36	39.9	672	2.7	
	cis-spliced introns	-	-	-	-	-	-	-	
	tRNA genes	27.2	25.2	24.5	23.1	49.7	147	0.6	
	rRNA genes	-	-	-	-	-	-	-	
	Non-coding regions	28	21.6	22.4	28	44	24205	96.7	
Chr7	Whole genome	26.9	22.5	23.5	27.1	46	27987	100	61.0X
	Protein-coding genes	25.7	23.2	20	31.1	43.2	2223	7.9	
	cis-spliced introns	-	-	-	-	-	-	-	
	tRNA genes	19.3	29.5	31.9	19.3	61.4	166	0.6	
	rRNA genes	-	-	-	-	-	-	-	
	Non-coding regions	27.1	22.4	23.7	26.8	46.1	25598	91.5	
Chr8	Whole genome	28.9	21.1	21.1	28.9	42.2	30648	100	71.0X
	Protein-coding genes	23.2	26.7	19.5	30.7	46.1	570	1.9	
	cis-spliced introns	-	-	-	-	-	-	-	
	tRNA genes	-	-	-	-	-	-	-	
	rRNA genes	-	-	-	-	-	-	-	
	Non-coding regions	29	21	21.1	28.8	42.1	30078	98.1	
Chr9	Whole genome	31.5	19.5	20.5	28.5	40	27130	100	47.0X
	Protein-coding genes	31.4	20.2	20.8	27.5	41.1	2283	8.4	
	cis-spliced introns	-	-	-	-	-	-	-	
	tRNA genes	30.7	22.8	19	27.4	41.8	521	1.9	
	rRNA genes	-	-	-	-	-	-	-	
	Non-coding regions	31.5	19.4	20.5	28.6	39.9	24326	89.7	
Chr10	Whole genome	30.1	20.1	21.6	28.2	41.7	25384	100	63.0X
	Protein-coding genes	28.1	25.3	19.5	27.1	44.8	672	2.6	
	cis-spliced introns	-	-	-	-	-	-	-	
	tRNA genes	30.1	27.4	20.5	21.9	47.9	73	0.3	
	rRNA genes	-	-	-	-	-	-	-	
	Non-coding regions	30.2	19.9	21.6	28.2	41.6	24639	97.1	
Chr11	Whole genome	28.3	21.9	23.4	26.5	45.2	22492	100	23.0X
	Protein-coding genes	32.3	19.7	22.4	25.5	42.2	1525	6.8	
	cis-spliced introns	-	-	-	-	-	-	-	

Genome	Feature	A %	C %	G %	T %	GC %	Size (bp)	Proportion in Genome (%)	Mean depth
	tRNA genes	27.4	24.7	24.7	23.3	49.3	73	0.3	
	rRNA genes	-	-	-	-	-	-	-	
	Non-coding regions	28	22	23.4	26.6	45.4	20894	92.9	
Chr12	Whole genome	27.8	21.7	22.4	28.1	44.1	30016	100	92.0X
	Protein-coding genes	23.9	24.1	18.9	33.1	43	723	2.4	
	cis-spliced introns	-	-	-	-	-	-	-	
	tRNA genes	-	-	-	-	-	-	-	
	rRNA genes	-	-	-	-	-	-	-	
	Non-coding regions	27.9	21.7	22.5	27.9	44.2	29293	97.6	
Chr13	Whole genome	27.1	22.7	23.4	26.8	46.1	21336	100	30.0X
	Protein-coding genes	26	25.2	24.1	24.8	49.3	2980	14	
	cis-spliced introns	-	-	-	-	-	-	-	
	tRNA genes	-	-	-	-	-	-	-	
	rRNA genes	-	-	-	-	-	-	-	
	Non-coding regions	27.3	22.3	23.3	27.2	45.6	18356	86	
Chr14	Whole genome	26.7	22.4	22.2	28.7	44.6	19950	100	57.0X
	Protein-coding genes	22.8	21.7	23.8	31.7	45.5	747	3.7	
	cis-spliced introns	-	-	-	-	-	-	-	
	tRNA genes	25.5	27.3	25.5	21.8	52.7	220	1.1	
	rRNA genes	-	-	-	-	-	-	-	
	Non-coding regions	26.9	22.3	22.1	28.6	44.5	18983	95.2	
Chr15	Whole genome	27.9	21.9	22.2	28	44.1	31824	100	62.0X
	Protein-coding genes	24.9	20.7	22.2	32.2	42.9	2037	6.4	
	cis-spliced introns	-	-	-	-	-	-	-	
	tRNA genes	25.4	23.9	26.8	23.9	50.7	71	0.2	
	rRNA genes	-	-	-	-	-	-	-	
	Non-coding regions	28.1	21.9	22.2	27.7	44.2	29716	93.4	
Chr16	Whole genome	28.8	22	21.4	27.8	43.4	26845	100	58.0X
	Protein-coding genes	30.5	21.1	22.5	25.9	43.6	2324	8.7	
	cis-spliced introns	-	-	-	-	-	-	-	
	tRNA genes	-	-	-	-	-	-	-	
	rRNA genes	-	-	-	-	-	-	-	

Genome	Feature	A %	C %	G %	T %	GC %	Size (bp)	Proportion in Genome (%)	Mean depth
	Non-coding regions	28.6	22.1	21.3	28	43.3	24521	91.3	
Chr17	Whole genome	26.9	22.8	21.2	29.1	44	39700	100	41.0X
	Protein-coding genes	28.3	23.4	22.9	25.4	46.3	3240	8.2	
	cis-spliced introns	-	-	-	-	-	-	-	
	tRNA genes	25	20.8	27.8	26.4	48.6	72	0.2	
	rRNA genes	-	-	-	-	-	-	-	
	Non-coding regions	26.8	22.7	21	29.4	43.7	36388	91.7	
Chr18	Whole genome	28.6	22.4	22.1	27	44.5	40145	100	106.0X
	Protein-coding genes	28.9	20.3	21.1	29.7	41.4	2175	5.4	
	cis-spliced introns	-	-	-	-	-	-	-	
	tRNA genes	-	-	-	-	-	-	-	
	rRNA genes	-	-	-	-	-	-	-	
	Non-coding regions	28.5	22.5	22.2	26.8	44.7	37970	94.6	
Chr19	Whole genome	27.4	21.7	22.7	28.2	44.4	17890	100	45.0X
	Protein-coding genes	28.4	20.8	23	27.7	43.9	1185	6.6	
	cis-spliced introns	-	-	-	-	-	-	-	
	tRNA genes	19.4	23.6	26.4	30.6	50	72	0.4	
	rRNA genes	-	-	-	-	-	-	-	
	Non-coding regions	27.3	21.8	22.6	28.3	44.4	16633	93	
Chr20	Whole genome	27.6	21.9	22.4	28.2	44.2	44494	100	100.0X
	Protein-coding genes	30.7	19.5	26.7	23.2	46.1	570	1.3	
	cis-spliced introns	-	-	-	-	-	-	-	
	tRNA genes	24.7	21.6	27.2	26.5	48.8	162	0.4	
	rRNA genes	25.8	23.1	29.3	21.8	52.3	3427	7.7	
	Non-coding regions	27.7	21.8	21.7	28.8	43.5	40335	90.7	
Chr21	Whole genome	29	22.4	21	27.6	43.4	22384	100	57.0X
	Protein-coding genes	-	-	-	-	-	-	-	
	cis-spliced introns	-	-	-	-	-	-	-	
	tRNA genes	22.6	19.4	24.7	33.3	44.1	93	0.4	
	rRNA genes	21	30.3	23.7	25	54	2100	9.4	
	Non-coding regions	29.9	21.6	20.7	27.8	42.3	20191	90.2	
Chr22	Whole genome	30.1	20.6	19.2	30	39.8	26237	100	27.0X

Genome	Feature	A %	C %	G %	T %	GC %	Size (bp)	Proportion in Genome (%)	Mean depth
	Protein-coding genes	35.3	16.9	25	22.9	41.9	621	2.4	
	cis-spliced introns	-	-	-	-	-	-	-	
	tRNA genes	22.6	27.3	26	24.1	53.3	619	2.4	
	rRNA genes	-	-	-	-	-	-	-	
	Non-coding regions	30.2	20.5	18.9	30.3	39.5	24997	95.3	

Table S3. SSRs in the mitochondrial genome of *D. officinale*.

Chromosomes	SSR nr.	SSR type	SSR	size	start	end
Chr1	1	p1	(A)10	10	1965	1974
Chr1	2	p1	(A)10	10	6836	6845
Chr1	3	p1	(T)10	10	18252	18261
Chr3	1	p1	(T)10	10	20118	20127
Chr3	2	p1	(T)10	10	20833	20842
Chr3	3	p2	(TA)6	12	29338	29349
Chr3	4	p1	(T)10	10	29924	29933
Chr4	1	p2	(TA)7	14	2919	2932
Chr4	2	p1	(A)10	10	3837	3846
Chr4	3	p1	(T)11	11	14302	14312
Chr4	4	p3	(GAA)5	15	16368	16382
Chr5	1	p1	(T)10	10	5730	5739
Chr6	1	p1	(A)12	12	8831	8842
Chr6	2	p1	(T)10	10	20654	20663
Chr7	1	p1	(T)10	10	11673	11682
Chr7	2	p2	(TA)7	14	12274	12287
Chr7	3	p1	(A)12	12	25928	25939
Chr7	4	p1	(A)10	10	27561	27570
Chr8	1	p1	(T)11	11	10995	11005
Chr8	2	p1	(A)10	10	14559	14568
Chr8	3	p1	(A)10	10	30582	30591
Chr9	1	p1	(A)10	10	1191	1200
Chr9	2	p1	(A)10	10	3871	3880
Chr10	1	p1	(A)10	10	12831	12840
Chr10	2	p1	(A)10	10	16197	16206
Chr10	3	p1	(T)10	10	21409	21418
Chr11	1	p1	(T)10	10	2358	2367
Chr11	2	p1	(T)13	13	8583	8595
Chr11	3	p1	(A)10	10	19548	19557
Chr11	4	p1	(A)10	10	21210	21219
Chr11	5	p1	(A)11	11	21367	21377
Chr12	1	p1	(T)10	10	29060	29069
Chr13	1	p2	(TA)6	12	2541	2552
Chr13	2	p1	(T)12	12	21286	21297
Chr14	1	p1	(T)10	10	3800	3809
Chr15	1	p1	(T)11	11	13918	13928
Chr16	1	p1	(T)11	11	8396	8406
Chr16	2	p1	(T)11	11	25424	25434
Chr17	1	p1	(T)11	11	19863	19873
Chr18	1	p1	(A)10	10	13481	13490
Chr19	1	p1	(A)10	10	2466	2475
Chr19	2	p1	(T)10	10	9930	9939

Chromosomes	SSR nr.	SSR type	SSR	size	start	end
Chr19	3	p1	(A)10	10	14976	14985
Chr20	1	p1	(T)10	10	4525	4534
Chr20	2	p1	(A)11	11	8094	8104
Chr20	3	p1	(G)10	10	17809	17818
Chr20	4	p1	(T)11	11	24407	24417
Chr20	5	p1	(T)10	10	32996	33005
Chr22	1	p1	(A)11	11	1853	1863
Chr22	2	p1	(A)10	10	12944	12953
Chr22	4	p2	(TA)6	12	18394	18405
Chr22	5	p1	(A)10	10	24257	24266
Chr22	6	p1	(A)11	11	25518	25528

Table S4. Tandem repeat sequences in the mitochondrial genome of *D. officinale.*

NO.	Chr	Size	Repeat sequence	Copy	Percent Matchres	Start	End
1	Chr2	22	GAAAGCATATGTGACATATGAG	1.9	95	1581	1622
2	Chr3	17	TTCTTTCTTAAGACTTCC	2.7	76	27692	27737
3	Chr3	18	TTCTTAAGATGAATAGAA	2.1	90	27730	27766
4	Chr3	13	TTCTAATGTAATA	2	100	28107	28132
5	Chr3	19	AATACATAATTCTTACATA	2.1	86	28701	28741
6	Chr4	13	CTATAATAGAAT	3.3	90	3652	3691
7	Chr4	18	CAACCGTACCCGCAACAA	2.1	95	9293	9330
8	Chr4	15	GCAACCGTACCCGACA	3.1	77	9292	9341
9	Chr4	21	TACGAAAAGATCGCTTGGATT	2.1	86	13410	13453
10	Chr4	15	GTTTGCTTATTCCTT	2	93	13460	13489
11	Chr4	20	AATTCACTTACAATGAATGAA	2.3	82	18746	18791
12	Chr4	17	TTACTAACAATTACTA	2.1	88	41065	41098
13	Chr5	20	AAAAGAAAATAGAAAGAAC	2.4	80	15398	15444
14	Chr6	16	TTGAAGCATTAAGTAT	1.9	93	285	315
15	Chr6	16	TTCTTATTCTTTCAAT	2.3	90	1510	1546
16	Chr6	28	GCCTTTTCTTATTTCAGAATCAACTAT T	2.8	68	23078	23157
17	Chr7	12	TACGAAAGCTGG	2.3	100	14797	14824
18	Chr7	25	CGACTGAAAGGAGAGGAGACAGGCT	2.6	74	19350	19412
19	Chr8	27	ATTAGAATTACCTCTGAACCATAGGA A	2.2	90	3902	3961
20	Chr9	16	AGAAAAATGAATATGA	2.9	81	10424	10471
21	Chr10	17	TGTAAACCCCAGAACAG	2	94	36	69
22	Chr10	13	TCTAGCGCCTTTA	1.9	100	9819	9843
23	Chr10	15	TATACGAAAGATCGT	2	93	18650	18679
24	Chr10	13	CATTAACTATTTA	2.1	100	25234	25260
25	Chr11	15	ACGATATTTCGTATA	2	93	10910	10939
26	Chr12	15	TATACGAAAGATCGT	2	93	13992	14021
27	Chr12	11	CCACTCTTCAC	2.3	100	15742	15766
28	Chr15	15	CTGTATAATTAGGAC	2.5	95	27123	27159
29	Chr17	15	GATACTGTGGTCTCT	3.5	78	9021	9072
30	Chr19	19	ATACATATCTATATCTTTC	2.2	90	10236	10276
31	Chr20	27	TCTAATTTCATATGGTTAAAAGGTAA T	2.2	90	28978	29037

NO.	Chr	Size	Repeat sequence	Copy	Percent Matchres	Start	End
32	Chr21	15	TATACGAAAGATCGT	3	96	22110	22154
33	Chr22	26	AGATTTTTCTATTTCATACTCTATTAA	1.9	84	16242	16292
34	Chr22	10	TATGGTCTAT	3.4	92	16307	16341
35	Chr22	2	TA	43.5	74	18140	18223
36	Chr22	22	TATATATATAATATAATATA	4.5	75	18122	18222

Table S5. Dispersed repeat sequences in the mitochondrial genome of *D. officinale.*

Chr	type	alignment	start1	end1	start2	end2	evalue
Chr3	D	51	27938	27988	28008	28058	3.52e-17
Chr3	D	50	27935	27984	28005	28054	1.33e-16
Chr3	D	52	27932	27983	28002	28053	9.34e-18
Chr3	D	44	27928	27971	27998	28041	3.67e-13
Chr3	D	50	9872	9921	15244	15293	1.33e-16
Chr3	D	50	9871	9920	15243	15292	1.33e-16
Chr3	D	49	9870	9918	15242	15290	4.98e-16
Chr3	D	49	9869	9917	15241	15289	4.98e-16
Chr4	D	45	21671	21715	35387	35431	1.71e-13
Chr4	D	41	21666	21706	35382	35422	3.29e-11
Chr4	D	46	21660	21705	35376	35421	4.58e-14
Chr4	D	34	21655	21688	35371	35404	3.03e-07
Chr4	P	35	30555	30589	33100	33134	8.28e-08
Chr4	P	35	30554	30588	33101	33135	8.28e-08
Chr4	P	34	30553	30586	33103	33136	3.03e-07
Chr5	D	34	6671	6704	14026	14059	8.73e-08
Chr5	D	34	6670	6703	14025	14058	8.73e-08
Chr5	D	33	6669	6701	14024	14056	3.18e-07
Chr5	D	34	6667	6700	14022	14055	8.73e-08
Chr6	D	41	12534	12574	21348	21388	1.05e-11
Chr6	D	40	12533	12572	21347	21386	3.89e-11
Chr6	D	41	12531	12571	21345	21385	1.05e-11
Chr6	D	41	12530	12570	21344	21384	1.05e-11
Chr6	P	79	12924	13002	23067	23145	1.03e-33
Chr6	P	59	12923	12981	23088	23146	4.65e-22
Chr6	P	57	12922	12978	23091	23147	6.70e-21
Chr6	P	58	12920	12977	23092	23149	1.77e-21
Chr7	D	39	12963	13001	17701	17739	1.8e-10
Chr7	D	39	12962	13000	17700	17738	1.8e-10
Chr7	D	40	12960	12999	17698	17737	4.86e-11
Chr7	D	34	12959	12992	17697	17730	1.21e-07
Chr7	D	44	1345	1388	26465	26508	2.55e-13
Chr7	D	47	1340	1386	26460	26506	4.87e-15

Chr	type	alignment	start1	end1	start2	end2	evalue
Chr7	D	45	1339	1383	26459	26503	6.82e-14
Chr7	D	36	1338	1373	26458	26493	8.99e-09
Chr7	P	36	8978	9013	17695	17730	8.99e-09
Chr7	P	41	8971	9011	17697	17737	1.31e-11
Chr7	P	42	8969	9010	17698	17739	3.53e-12
Chr7	P	40	8968	9007	17701	17740	4.86e-11
Chr7	P	43	8970	9012	12958	13000	9.49e-13
Chr7	P	44	8968	9011	12959	13002	2.55e-13
Chr7	P	45	8966	9010	12960	13004	6.82e-14
Chr7	P	44	8965	9008	12962	13005	2.55e-13
Chr8	D	45	17339	17383	29333	29377	8.18e-14
Chr8	D	36	17338	17373	29332	29367	1.08e-08
Chr8	D	36	4957	4992	10852	10887	1.08e-08
Chr8	D	36	17354	17389	29348	29383	1.08e-08
Chr8	D	32	17353	17384	29347	29378	1.92e-06
Chr8	D	33	3901	3933	3928	3960	5.27e-07
Chr8	P	39	10881	10919	29337	29375	2.16e-10
Chr8	P	37	10880	10916	29340	29376	2.93e-09
Chr8	P	35	10875	10909	29347	29381	3.95e-08
Chr8	P	35	10874	10908	29348	29382	3.95e-08
Chr8	P	40	10881	10920	17342	17381	5.83e-11
Chr8	P	40	10880	10919	17343	17382	5.83e-11
Chr8	P	38	10879	10916	17346	17383	7.96e-10
Chr8	P	33	10889	10921	17341	17373	5.27e-07
Chr8	P	31	9715	9745	16015	16045	6.95e-06
Chr8	P	31	9714	9744	16016	16046	6.95e-06
Chr9	D	30	3436	3465	3565	3594	1.97e-05
Chr9	D	31	3450	3480	3507	3537	5.45e-06
Chr10	D	34	8448	8481	11467	11500	9.92e-08
Chr10	D	33	8447	8479	11466	11498	3.62e-07
Chr10	D	34	8445	8478	11464	11497	9.92e-08
Chr10	D	33	8444	8476	11463	11495	3.62e-07
Chr13	D	51	13122	13172	13192	13242	1.42e-17
Chr13	D	50	13119	13168	13189	13238	5.34e-17

Chr	type	alignment	start1	end1	start2	end2	evalue
Chr13	D	52	13116	13167	13186	13237	3.77e-18
Chr13	D	44	13112	13155	13182	13225	1.48e-13
Chr14	D	32	3298	3329	7135	7166	8.13e-07
Chr14	D	33	3296	3328	7133	7165	2.23e-07
Chr14	D	33	3295	3327	7132	7164	2.23e-07
Chr14	D	34	3293	3326	7130	7163	6.13e-08
Chr15	D	52	5089	5140	5159	5210	8.38e-18
Chr15	D	50	5088	5137	5158	5207	1.19e-16
Chr15	D	51	5084	5134	5154	5204	3.16e-17
Chr15	D	44	5101	5144	5171	5214	3.29e-13
Chr15	D	32	485	516	11437	11468	2.07e-06
Chr15	D	32	484	515	11436	11467	2.07e-06
Chr15	P	47	23276	23322	31339	31385	6.3e-15
Chr15	P	48	23274	23321	31340	31387	1.68e-15
Chr15	P	48	23273	23320	31341	31388	1.68e-15
Chr15	P	50	23270	23319	31342	31391	1.19e-16
Chr15	P	30	14452	14481	18829	18858	2.71e-05
Chr16	D	31	2433	2463	15314	15344	5.33e-06
Chr16	D	31	2432	2462	15313	15343	5.33e-06
Chr16	D	31	2431	2461	15312	15342	5.33e-06
Chr17	D	51	7441	7491	31870	31920	4.92e-17
Chr17	D	39	8745	8783	33178	33216	3.62e-10
Chr17	D	72	6644	6715	31063	31134	3.2e-29
Chr17	D	52	9302	9353	33720	33771	1.3e-17
Chr17	D	50	9301	9350	33719	33768	1.85e-16
Chr17	D	46	9298	9343	33716	33761	3.67e-14
Chr17	D	44	9599	9642	34022	34065	5.12e-13
Chr17	D	34	8893	8926	33326	33359	2.43e-07
Chr17	D	36	8888	8923	33321	33356	1.81e-08
Chr17	D	32	8884	8915	33317	33348	3.22e-06
Chr17	D	31	8882	8912	33315	33345	1.17e-05
Chr17	D	51	5112	5162	29534	29584	4.92e-17
Chr17	D	55	5107	5161	29529	29583	2.42e-19
Chr17	D	55	5106	5160	29528	29582	2.42e-19

Chr	type	alignment	start1	end1	start2	end2	evalue
Chr17	D	32	8287	8318	32725	32756	3.22e-06
Chr17	D	31	8286	8316	32724	32754	1.17e-05
Chr17	D	86	7493	7578	31922	32007	2.05e-37
Chr17	D	57	7192	7248	31620	31676	1.69e-20
Chr17	D	63	7185	7247	31613	31675	5.59e-24
Chr17	D	58	8158	8215	32596	32653	4.45e-21
Chr17	D	59	8156	8214	32594	32652	1.17e-21
Chr17	D	72	8383	8454	32821	32892	3.2e-29
Chr17	D	40	4152	4191	28576	28615	9.78e-11
Chr17	D	329	2926	3254	27347	27675	5.89e-182
Chr17	D	35	10190	10224	34604	34638	6.64e-08
Chr17	D	281	5419	5699	29840	30120	2.9e-153
Chr17	D	74	5812	5885	30233	30306	2.17e-30
Chr17	D	63	6278	6340	30697	30759	5.59e-24
Chr17	D	59	6108	6166	30527	30585	1.17e-21
Chr17	D	67	6168	6234	30587	30653	2.63e-26
Chr17	D	367	12745	13111	37159	37525	1.08e-204
Chr17	D	51	7208	7258	31636	31686	4.92e-17
Chr17	D	110	5701	5810	30122	30231	1.53e-51
Chr17	D	56	9581	9636	34004	34059	6.39e-20
Chr17	D	59	9575	9633	33998	34056	1.17e-21
Chr17	D	34	9573	9606	33996	34029	2.43e-07
Chr17	D	62	9448	9509	33866	33927	2.13e-23
Chr17	D	95	7544	7638	31973	32067	1.05e-42
Chr17	D	1515	11409	12923	35823	37337	0
Chr17	D	1341	11403	12743	35817	37157	0
Chr17	D	47	11401	11447	35815	35861	9.8e-15
Chr17	D	48	11396	11443	35810	35857	2.61e-15
Chr17	D	100	6543	6642	30962	31061	1.2e-45
Chr17	D	87	6539	6625	30958	31044	5.3e-38
Chr17	D	89	6533	6621	30952	31040	3.55e-39
Chr17	D	91	9136	9226	33554	33644	2.36e-40
Chr17	D	74	9908	9981	34322	34395	2.17e-30
Chr17	D	74	9905	9978	34319	34392	2.17e-30

Chr	type	alignment	start1	end1	start2	end2	evalue
Chr17	D	57	7250	7306	31678	31734	1.69e-20
Chr17	D	31	7249	7279	31677	31707	1.17e-05
Chr17	D	345	0	344	24421	24765	1.57e-191
Chr17	D	98	7526	7623	31955	32052	1.81e-44
Chr17	D	87	6060	6146	30479	30565	5.3e-38
Chr17	D	62	7580	7641	32009	32070	2.13e-23
Chr17	D	80	5979	6058	30398	30477	6.73e-34
Chr17	D	53	5978	6030	30397	30449	3.46e-18
Chr17	D	52	5977	6028	30396	30447	1.3e-17
Chr17	D	44	5976	6019	30395	30438	5.12e-13
Chr17	D	83	9247	9329	33665	33747	1.18e-35
Chr17	D	424	5162	5585	29583	30006	8.04e-239
Chr17	D	378	5161	5538	29582	29959	2.82e-211
Chr17	D	258	5160	5417	29581	29838	1.58e-139
Chr17	D	139	5159	5297	29580	29718	1.08e-68
Chr17	D	151	3254	3404	27676	27826	8.26e-76
Chr17	D	109	3253	3361	27675	27783	5.96e-51
Chr17	D	42	3251	3292	27673	27714	7.1e-12
Chr17	D	40	3250	3289	27672	27711	9.78e-11
Chr17	D	104	7511	7614	31940	32043	5.29e-48
Chr17	D	38	7625	7662	32054	32091	1.34e-09
Chr17	D	1098	13170	14267	37580	38677	0
Chr17	D	62	7799	7860	32228	32289	2.13e-23
Chr17	D	30	7034	7063	31456	31485	4.21e-05
Chr17	D	30	7281	7310	31709	31738	4.21e-05
Chr17	D	31	10836	10866	35250	35280	1.17e-05
Chr17	D	31	10835	10865	35249	35279	1.17e-05
Chr17	D	34	7110	7143	31538	31571	2.43e-07
Chr17	D	34	10259	10292	34673	34706	2.43e-07
Chr17	D	36	10255	10290	34669	34704	1.81e-08
Chr17	D	33	8764	8796	33197	33229	8.85e-07
Chr17	D	45	6305	6349	30724	30768	1.37e-13
Chr17	D	188	12925	13112	37339	37526	8.47e-98
Chr17	D	125	8638	8762	33071	33195	2.1e-60

Chr	type	alignment	start1	end1	start2	end2	evalue
Chr17	D	94	7766	7859	32195	32288	4.09e-42
Chr17	D	30	10495	10524	34909	34938	4.21e-05
Chr17	D	71	7391	7461	31820	31890	1.23e-28
Chr17	D	74	7385	7458	31814	31887	2.17e-30
Chr17	D	58	7382	7439	31811	31868	4.45e-21
Chr17	D	32	7379	7410	31808	31839	3.22e-06
Chr17	D	83	8392	8474	32830	32912	1.18e-35
Chr17	D	41	7269	7309	31697	31737	2.64e-11
Chr17	D	33	5860	5892	30281	30313	8.85e-07
Chr17	D	30	10085	10114	34499	34528	4.21e-05
Chr17	D	49	7260	7308	31688	31736	6.96e-16
Chr17	D	74	5785	5858	30206	30279	2.17e-30
Chr17	D	197	5587	5783	30008	30204	3.72e-103
Chr17	D	46	8145	8190	32583	32628	3.67e-14
Chr17	D	109	9115	9223	33533	33641	5.96e-51
Chr17	D	395	4476	4870	28898	29292	1.86e-221
Chr17	D	390	4475	4864	28897	29286	1.85e-218
Chr17	D	57	7150	7206	31578	31634	1.69e-20
Chr17	D	45	7146	7190	31574	31618	1.37e-13
Chr17	D	39	7145	7183	31573	31611	3.62e-10
Chr17	D	499	13772	14270	38182	38680	9.2e-284
Chr17	D	37	7969	8005	32407	32443	4.92e-09
Chr17	D	34	8241	8274	32679	32712	2.43e-07
Chr17	D	48	8109	8156	32547	32594	2.61e-15
Chr17	D	52	8103	8154	32541	32592	1.3e-17
Chr17	D	64	11023	11086	35445	35508	1.46e-24
Chr17	D	57	11018	11074	35440	35496	1.69e-20
Chr17	D	50	11013	11062	35435	35484	1.85e-16
Chr17	D	36	11007	11042	35429	35464	1.81e-08
Chr17	D	35	6950	6984	31372	31406	6.64e-08
Chr17	D	37	6943	6979	31365	31401	4.92e-09
Chr17	D	202	3294	3495	27716	27917	3.92e-106
Chr17	D	204	3291	3494	27713	27916	2.52e-107
Chr17	D	58	8129	8186	32567	32624	4.45e-21

Chr	type	alignment	start1	end1	start2	end2	evalue
Chr17	D	238	3913	4150	28337	28574	1.36e-127
Chr17	D	340	2914	3253	27335	27674	1.54e-188
Chr17	D	329	2913	3241	27334	27662	5.89e-182
Chr17	D	325	2912	3236	27333	27657	1.45e-179
Chr17	D	2469	337	2805	24759	27227	0
Chr17	D	2470	335	2804	24757	27226	0
Chr17	D	2470	334	2803	24756	27225	0
Chr17	D	2470	333	2802	24755	27224	0
Chr17	D	64	9704	9767	34127	34190	1.46e-24
Chr17	D	45	7616	7660	32045	32089	1.37e-13
Chr17	D	75	5036	5110	29458	29532	5.66e-31
Chr17	D	1614	11449	13062	35863	37476	0
Chr17	D	1615	11445	13059	35859	37473	0
Chr17	D	234	4872	5105	29294	29527	3.31e-125
Chr17	D	239	4866	5104	29288	29526	3.45e-128
Chr17	D	49	11044	11092	35466	35514	6.96e-16
Chr17	D	341	5299	5639	29720	30060	3.91e-189
Chr17	D	33	11064	11096	35486	35518	8.85e-07
Chr17	D	65	5906	5970	30326	30390	3.84e-25
Chr17	D	63	5905	5967	30325	30387	5.59e-24
Chr17	D	52	7050	7101	31472	31523	1.3e-17
Chr17	D	50	7047	7096	31469	31518	1.85e-16
Chr17	D	36	7044	7079	31466	31501	1.81e-08
Chr17	D	30	8323	8352	32761	32790	4.21e-05
Chr17	D	32	8320	8351	32758	32789	3.22e-06
Chr17	D	30	8318	8347	32756	32785	4.21e-05
Chr17	D	69	9922	9990	34336	34404	1.80e-27
Chr17	D	39	9770	9808	34193	34231	3.62e-10
Chr17	D	34	9769	9802	34192	34225	2.43e-07
Chr17	D	35	9767	9801	34190	34224	6.64e-08
Chr17	D	99	9105	9203	33523	33621	4.66e-45
Chr17	D	64	9098	9161	33516	33579	1.46e-24
Chr17	D	38	9097	9134	33515	33552	1.34e-09
Chr17	D	31	10613	10643	35027	35057	1.17e-05

Chr	type	alignment	start1	end1	start2	end2	evalue
Chr17	D	32	10610	10641	35024	35055	3.22e-06
Chr17	D	32	2833	2864	27258	27289	3.22e-06
Chr17	D	33	2831	2863	27256	27288	8.85e-07
Chr17	D	74	7469	7542	31898	31971	2.17e-30
Chr17	D	62	7463	7524	31892	31953	2.13e-23
Chr17	D	50	7460	7509	31889	31938	1.85e-16
Chr17	D	74	9608	9681	34031	34104	2.17e-30
Chr17	D	32	8007	8038	32445	32476	3.22e-06
Chr17	D	32	2818	2849	27243	27274	3.22e-06
Chr17	D	74	7643	7716	32072	32145	2.17e-30
Chr17	D	62	7640	7701	32069	32130	2.13e-23
Chr17	D	64	7664	7727	32093	32156	1.46e-24
Chr17	D	63	7662	7724	32091	32153	5.59e-24
Chr17	D	56	7412	7467	31841	31896	6.39e-20
Chr17	D	1155	13112	14266	37522	38676	0
Chr17	D	1154	13111	14264	37521	38674	0
Chr17	D	661	13110	13770	37520	38180	0
Chr17	D	60	13109	13168	37519	37578	3.08e-22
Chr17	D	69	9232	9300	33650	33718	1.80e-27
Chr17	D	72	9228	9299	33646	33717	3.2e-29
Chr17	D	72	9225	9296	33643	33714	3.2e-29
Chr17	D	31	8456	8486	32894	32924	1.17e-05
Chr17	D	42	7990	8031	32428	32469	7.1e-12
Chr17	D	38	7985	8022	32423	32460	1.34e-09
Chr17	D	51	8057	8107	32495	32545	4.92e-17
Chr17	D	41	9205	9245	33623	33663	2.64e-11
Chr17	D	211	5540	5750	29961	30171	1.71e-111
Chr17	D	144	4045	4188	28469	28612	1.17e-71
Chr17	D	136	4039	4174	28463	28598	6.47e-67
Chr17	D	44	6717	6760	31136	31179	5.12e-13
Chr17	D	39	7347	7385	31769	31807	3.62e-10
Chr17	D	42	7341	7382	31763	31804	7.1e-12
Chr17	D	41	7335	7375	31757	31797	2.64e-11
Chr17	D	1019	14263	15281	38681	39699	0

Chr	type	alignment	start1	end1	start2	end2	evalue
Chr17	D	36	8754	8789	33187	33222	1.81e-08
Chr17	D	116	4075	4190	28499	28614	4.4e-55
Chr17	D	44	11340	11383	35754	35797	5.12e-13
Chr17	D	45	11336	11380	35750	35794	1.37e-13
Chr17	D	45	11332	11376	35746	35790	1.37e-13
Chr17	D	49	11326	11374	35740	35788	6.96e-16
Chr17	D	39	9983	10021	34397	34435	3.62e-10
Chr17	D	35	9980	10014	34394	34428	6.64e-08
Chr17	D	30	9973	10002	34387	34416	4.21e-05
Chr17	D	35	9992	10026	34406	34440	6.64e-08
Chr17	D	386	3652	4037	28076	28461	4.57e-216
Chr17	D	54	5836	5889	30257	30310	9.15e-19
Chr17	D	98	9644	9741	34067	34164	1.81e-44
Chr17	D	77	9638	9714	34061	34137	3.83e-32
Chr17	D	68	9635	9702	34058	34125	6.89e-27
Chr17	D	90	6668	6757	31087	31176	9.17e-40
Chr17	D	62	8066	8127	32504	32565	2.13e-23
Chr17	D	53	9716	9768	34139	34191	3.46e-18
Chr17	D	245	4389	4633	28811	29055	9.06e-132
Chr17	D	371	3541	3911	27965	28335	4.37e-207
Chr17	D	38	7065	7102	31487	31524	1.34e-09
Chr17	D	50	10711	10760	35125	35174	1.85e-16
Chr17	D	50	10710	10759	35124	35173	1.85e-16
Chr17	D	50	10706	10755	35120	35169	1.85e-16
Chr17	D	71	6084	6154	30503	30573	1.23e-28
Chr17	D	225	14262	14486	38680	38904	7.71e-120
Chr17	D	51	13064	13114	37478	37528	4.92e-17
Chr17	D	53	13061	13113	37475	37527	3.46e-18
Chr17	D	52	8515	8566	32948	32999	1.3e-17
Chr17	D	51	8514	8564	32947	32997	4.92e-17
Chr17	D	33	10303	10335	34717	34749	8.85e-07
Chr17	D	43	8439	8481	32877	32919	1.91e-12
Chr17	D	81	5803	5883	30224	30304	1.75e-34
Chr17	D	43	9743	9785	34166	34208	1.91e-12

Chr	type	alignment	start1	end1	start2	end2	evalue
Chr17	D	36	7998	8033	32436	32471	1.81e-08
Chr17	D	65	7865	7929	32303	32367	3.84e-25
Chr17	D	46	7860	7905	32298	32343	3.67e-14
Chr17	D	43	7858	7900	32296	32338	1.91e-12
Chr17	D	34	6759	6792	31187	31220	2.43e-07
Chr17	D	33	6758	6790	31186	31218	8.85e-07
Chr17	D	33	6757	6789	31185	31217	8.85e-07
Chr17	D	34	6755	6788	31183	31216	2.43e-07
Chr17	D	55	6462	6516	30881	30935	2.42e-19
Chr17	D	57	6458	6514	30877	30933	1.69e-20
Chr17	D	55	6457	6511	30876	30930	2.42e-19
Chr17	D	44	6456	6499	30875	30918	5.12e-13
Chr17	D	36	10210	10245	34624	34659	1.81e-08
Chr17	D	40	10204	10243	34618	34657	9.78e-11
Chr17	D	47	7002	7048	31424	31470	9.8e-15
Chr17	D	47	6999	7045	31421	31467	9.8e-15
Chr17	D	45	6998	7042	31420	31464	1.37e-13
Chr17	D	37	6996	7032	31418	31454	4.92e-09
Chr17	D	59	6183	6241	30602	30660	1.17e-21
Chr17	D	338	3736	4073	28160	28497	2.43e-187
Chr17	D	311	3733	4043	28157	28467	3.41e-171
Chr17	D	34	6421	6454	30840	30873	2.43e-07
Chr17	D	33	8301	8333	32739	32771	8.85e-07
Chr17	D	64	6390	6453	30809	30872	1.46e-24
Chr17	D	68	6384	6451	30803	30870	6.89e-27
Chr17	D	47	6381	6427	30800	30846	9.8e-15
Chr17	D	42	6378	6419	30797	30838	7.1e-12
Chr17	D	67	6875	6941	31297	31363	2.63e-26
Chr17	D	53	6874	6926	31296	31348	3.46e-18
Chr17	D	40	6868	6907	31290	31329	9.78e-11
Chr17	D	59	8085	8143	32523	32581	1.17e-21
Chr17	D	34	10978	11011	35400	35433	2.43e-07
Chr17	D	30	10976	11005	35398	35427	4.21e-05
Chr17	D	44	8837	8880	33270	33313	5.12e-13

Chr	type	alignment	start1	end1	start2	end2	evalue
Chr17	D	142	4333	4474	28755	28896	1.8e-70
Chr17	D	143	4331	4473	28753	28895	4.59e-71
Chr17	D	58	4330	4387	28752	28809	4.45e-21
Chr17	D	72	6788	6859	31210	31281	3.2e-29
Chr17	D	73	6786	6858	31208	31280	8.33e-30
Chr17	D	38	6785	6822	31207	31244	1.34e-09
Chr17	D	37	6784	6820	31206	31242	4.92e-09
Chr17	D	48	8217	8264	32655	32702	2.61e-15
Chr17	D	47	8216	8262	32654	32700	9.8e-15
Chr17	D	114	6032	6145	30451	30564	6.66e-54
Chr17	D	77	6030	6106	30449	30525	3.83e-32
Chr17	D	33	11211	11243	35633	35665	8.85e-07
Chr17	D	32	11208	11239	35630	35661	3.22e-06
Chr17	D	32	11205	11236	35627	35658	3.22e-06
Chr17	D	32	11199	11230	35621	35652	3.22e-06
Chr17	D	68	9163	9230	33581	33648	6.89e-27
Chr17	D	72	8366	8437	32804	32875	3.2e-29
Chr17	D	36	8365	8400	32803	32838	1.81e-08
Chr17	D	30	8361	8390	32799	32828	4.21e-05
Chr17	D	134	3363	3496	27785	27918	9.89e-66
Chr17	D	48	2867	2914	27293	27340	2.61e-15
Chr17	D	53	2861	2913	27287	27339	3.46e-18
Chr17	D	45	2860	2904	27286	27330	1.37e-13
Chr17	D	37	2859	2895	27285	27321	4.92e-09
Chr17	D	35	10487	10521	34901	34935	6.64e-08
Chr17	D	36	10485	10520	34899	34934	1.81e-08
Chr17	D	83	5752	5834	30173	30255	1.18e-35
Chr17	D	81	6249	6329	30668	30748	1.75e-34
Chr17	D	82	6243	6324	30662	30743	4.53e-35
Chr17	D	68	6236	6303	30655	30722	6.89e-27
Chr17	D	74	8402	8475	32840	32913	2.17e-30
Chr17	D	61	6156	6216	30575	30635	8.09e-23
Chr17	D	31	10423	10453	34837	34867	1.17e-05
Chr17	D	55	6909	6963	31331	31385	2.42e-19

Chr	type	alignment	start1	end1	start2	end2	evalue
Chr17	D	505	4530	5034	28952	29456	2.33e-287
Chr17	D	58	6891	6948	31313	31370	4.45e-21
Chr17	D	74	8488	8561	32921	32994	2.17e-30
Chr17	D	68	8487	8554	32920	32987	6.89e-27
Chr17	D	53	8827	8879	33260	33312	3.46e-18
Chr17	D	55	7886	7940	32324	32378	2.42e-19
Chr17	D	50	10697	10746	35111	35160	1.85e-16
Chr17	D	31	10914	10944	35336	35366	1.17e-05
Chr17	D	233	3499	3731	27923	28155	1.31e-124
Chr17	D	154	3497	3650	27921	28074	1.37e-77
Chr17	D	44	3496	3539	27920	27963	5.12e-13
Chr17	D	37	7931	7967	32369	32405	4.92e-09
Chr17	D	440	4635	5074	29057	29496	2.09e-248
Chr17	D	43	8806	8848	33239	33281	1.91e-12
Chr17	D	42	8805	8846	33238	33279	7.1e-12
Chr17	D	32	8804	8835	33237	33268	3.22e-06
Chr17	D	47	9361	9407	33779	33825	9.8e-15
Chr17	D	47	9355	9401	33773	33819	9.8e-15
Chr17	D	47	9352	9398	33770	33816	9.8e-15
Chr17	D	45	9345	9389	33763	33807	1.37e-13
Chr17	D	96	6525	6620	30944	31039	2.72e-43
Chr17	D	34	10967	11000	35389	35422	2.43e-07
Chr17	D	50	9469	9518	33887	33936	1.85e-16
Chr17	D	58	10055	10112	34469	34526	4.45e-21
Chr17	D	56	10052	10107	34466	34521	6.39e-20
Chr17	D	49	10051	10099	34465	34513	6.96e-16
Chr17	D	41	9040	9080	33458	33498	2.64e-11
Chr17	D	37	9039	9075	33457	33493	4.92e-09
Chr17	D	72	6687	6758	31106	31177	3.2e-29
Chr17	D	161	5641	5801	30062	30222	9.56e-82
Chr17	D	58	5076	5133	29498	29555	4.45e-21
Chr17	D	146	4190	4335	28613	28758	7.64e-73
Chr17	D	147	4188	4334	28611	28757	1.95e-73
Chr17	D	151	4183	4333	28606	28756	8.26e-76

Chr	type	alignment	start1	end1	start2	end2	evalue
Chr17	D	152	4181	4332	28604	28755	2.11e-76
Chr17	D	55	8047	8101	32485	32539	2.42e-19
Chr17	D	42	8042	8083	32480	32521	7.1e-12
Chr17	D	49	6824	6872	31246	31294	6.96e-16
Chr17	D	45	6822	6866	31244	31288	1.37e-13
Chr17	D	53	7907	7959	32345	32397	3.46e-18
Chr17	D	43	7902	7944	32340	32382	1.91e-12
Chr17	D	90	8691	8780	33124	33213	9.17e-40
Chr17	D	59	6218	6276	30637	30695	1.17e-21
Chr17	D	32	6216	6247	30635	30666	3.22e-06
Chr17	D	31	10773	10803	35187	35217	1.17e-05
Chr17	D	30	10766	10795	35180	35209	4.21e-05
Chr17	D	30	10762	10791	35176	35205	4.21e-05
Chr17	D	49	7088	7136	31516	31564	6.96e-16
Chr17	D	51	7085	7135	31513	31563	4.92e-17
Chr17	D	40	7082	7121	31510	31549	9.78e-11
Chr17	D	135	8618	8752	33051	33185	2.53e-66
Chr17	D	133	8611	8743	33044	33176	3.87e-65
Chr17	D	82	8608	8689	33041	33122	4.53e-35
Chr17	D	83	9683	9765	34106	34188	1.18e-35
Chr17	D	62	6021	6082	30440	30501	2.13e-23
Chr17	D	67	6148	6214	30567	30633	2.63e-26
Chr17	D	35	6147	6181	30566	30600	6.64e-08
Chr17	D	121	7733	7853	32162	32282	4.88e-58
Chr17	D	119	7730	7848	32159	32277	7.42e-57
Chr17	D	69	7729	7797	32158	32226	1.80e-27
Chr17	D	39	7726	7764	32155	32193	3.62e-10
Chr17	D	36	7961	7996	32399	32434	1.81e-08
Chr17	D	43	7946	7988	32384	32426	1.91e-12
Chr17	D	42	7942	7983	32380	32421	7.1e-12
Chr17	D	59	6627	6685	31046	31104	1.17e-21
Chr17	D	44	6623	6666	31042	31085	5.12e-13
Chr17	D	33	6622	6654	31041	31073	8.85e-07
Chr17	D	181	4348	4528	28770	28950	1.24e-93

Chr	type	alignment	start1	end1	start2	end2	evalue
Chr17	D	77	9895	9971	34309	34385	3.83e-32
Chr17	D	35	6342	6376	30761	30795	6.64e-08
Chr17	D	84	6656	6739	31075	31158	3.05e-36
Chr17	D	74	5891	5964	30311	30384	2.17e-30
Chr17	D	74	5888	5961	30308	30381	2.17e-30
Chr17	D	36	6331	6366	30750	30785	1.81e-08
Chr17	D	66	8571	8636	33004	33069	1.01e-25
Chr17	D	49	8568	8616	33001	33049	6.96e-16
Chr17	D	44	8566	8609	32999	33042	5.12e-13
Chr17	D	44	8563	8606	32996	33039	5.12e-13
Chr17	D	37	8850	8886	33283	33319	4.92e-09
Chr17	D	35	8848	8882	33281	33315	6.64e-08
Chr17	D	37	6928	6964	31350	31386	4.92e-09
Chr17	D	213	3522	3734	27946	28158	1.09e-112
Chr17	D	59	9439	9497	33857	33915	1.17e-21
Chr17	D	59	9436	9494	33854	33912	1.17e-21
Chr17	D	35	9433	9467	33851	33885	6.64e-08
Chr17	D	95	3406	3500	27828	27922	1.05e-42
Chr17	D	44	10040	10083	34454	34497	5.12e-13
Chr17	D	77	7161	7237	31589	31665	3.83e-32
Chr17	D	48	8192	8239	32630	32677	2.61e-15
Chr17	D	51	8188	8238	32626	32676	4.92e-17
Chr17	P	30	6255	6284	30674	30703	4.21e-05
Chr17	P	31	6254	6284	6255	6285	1.17e-05
Chr18	D	32	14725	14756	18315	18346	3.29e-06
Chr18	D	30	3444	3473	20731	20760	4.31e-05
Chr20	D	47	1708	1754	34208	34254	1.23e-14
Chr20	D	47	1707	1753	34207	34253	1.23e-14
Chr20	D	51	1702	1752	34202	34252	6.17e-17
Chr20	D	39	1718	1756	34218	34256	4.55e-10
Chr20	D	33	24324	24356	28755	28787	1.11e-06
Chr20	D	33	24321	24353	28752	28784	1.11e-06
Chr20	D	33	24320	24352	28751	28783	1.11e-06
Chr20	D	33	14943	14975	24324	24356	1.11e-06

Chr	type	alignment	start1	end1	start2	end2	evalue
Chr20	D	33	14940	14972	24321	24353	1.11e-06
Chr20	D	33	14939	14971	24320	24352	1.11e-06
Chr20	D	44	8182	8225	14136	14179	6.43e-13
Chr20	D	43	8181	8223	14135	14177	2.4e-12
Chr20	D	43	8180	8222	14134	14176	2.4e-12
Chr20	D	43	8178	8220	14132	14174	2.4e-12
Chr20	D	36	8210	8245	14105	14140	2.27e-08
Chr20	D	240	14780	15019	28592	28831	1.09e-128
Chr20	D	240	14779	15018	28591	28830	1.09e-128
Chr20	D	239	14778	15016	28590	28828	4.32e-128
Chr20	D	239	14776	15014	28588	28826	4.32e-128
Chr20	D	33	28977	29009	29004	29036	1.11e-06
Chr20	P	35	8189	8223	34203	34237	8.33e-08
Chr20	P	35	8188	8222	34204	34238	8.33e-08
Chr20	P	37	8181	8217	34209	34245	6.18e-09
Chr20	P	39	8178	8216	34210	34248	4.55e-10
Chr20	P	34	14143	14176	34204	34237	3.05e-07
Chr20	P	33	14142	14174	34206	34238	1.11e-06
Chr20	P	36	14136	14171	34209	34244	2.27e-08
Chr20	P	37	14134	14170	34210	34246	6.18e-09
Chr20	P	35	1710	1744	14136	14170	8.33e-08
Chr20	P	30	1709	1738	14142	14171	5.29e-05
Chr20	P	30	1708	1737	14143	14172	5.29e-05
Chr20	P	31	3046	3076	9352	9382	1.47e-05
Chr20	P	31	3045	3075	9353	9383	1.47e-05
Chr20	P	36	1710	1745	8181	8216	2.27e-08
Chr20	P	30	1709	1738	8188	8217	5.29e-05
Chr20	P	30	1708	1737	8189	8218	5.29e-05
Chr20	P	31	1718	1748	8178	8208	1.47e-05
Chr21	D	32	22109	22140	22124	22155	1.02e-06
Chr21	D	32	22108	22139	22123	22154	1.02e-06
Chr21	D	32	22107	22138	22122	22153	1.02e-06
Chr22	D	53	13153	13205	17970	18022	1.51e-18
Chr22	D	52	13147	13198	17964	18015	5.7e-18

Chr	type	alignment	start1	end1	start2	end2	evalue
Chr22	D	52	13146	13197	17963	18014	5.7e-18
Chr22	D	51	13145	13195	17962	18012	2.15e-17

Table S6. RNA editing sites of protein-coding genes in mitochondrial genomes of *D. officinale*.

Name	pos	left	edit	right	score
atp1	atp1.t01!670	CACAATTAGTTCAAATTCTT	C	CAGAAGCGAATGCTTTGGAA	1
atp1	atp1.t01!971	CCAGACAGGTGCAGGTAGCT	C	GACTGCGTTACCCGTCATTG	1
atp1	atp1.t01!1039	ATATCCCCACCAATGTGATC	C	CCATTACAGATGGACAAATC	1
atp1	atp1.t01!1178	GAAACAAGTCCGCGGTAGTT	C	AAAACTAGAATTGGCACAAT	1
atp1	atp1.t01!1064	TACAGATGGACAAATCTGTT	C	GGAAACAGAGCTCTTTTATC	0.999
atp1	atp1.t01!1490	TAACGAAAGAAAGATGGAAC	C	AGATGCTTCTTTAAAAGAAA	0.999
atp1	atp1.t01!1262	TGCTGCGACTCAGGCATTAC	C	CAATAGAGGTGCGAGGCTGA	0.998
atp1	atp1.t01!1499	AAAGATGGAACCAGATGCTT	C	TTTAAAAGAAAGCGCTTTGC	0.997
atp1	atp1.t01!1292	TGCGAGGCTGACAGAAGTGC	C	CAAACAACCACAATATTCAC	0.996
atp1	atp1.t01!1168	TGAGAGCTATGAAACAAGTC	C	GCGGTAGTTCAAAACTAGAA	0.994
atp1	atp1.t01!1415	GAAATATGAGAGAGCCATTC	C	AAGTAGTATAGATCCCGAAT	0.992
atp4	atp4.t01!56	GATGCTATTTGCTGCTATTC	C	ATCTATTTGTGCATCAAGTC	1
atp4	atp4.t01!248	CAATCCTAACGAAGTAGTTC	C	TTCGGAATCCAATGAACAAC	1
atp4	atp4.t01!89	ATCAAGTCCGAAGAAGATCT	C	AATATATAATGAAGAAATGA	0.999
atp4	atp4.t01!227	GGAAGAATCGCAGCAATTCC	C	CAATCCTAACGAAGTAGTTC	0.999
atp4	atp4.t01!251	TCCTAACGAAGTAGTTCCTT	C	GGAATCCAATGAACAACAAC	0.999
atp4	atp4.t01!76	CATCTATTTGTGCATCAAGT	C	CGAAGAAGATCTCAATATAT	0.998
atp4	atp4.t01!71	TATTCCATCTATTTGTGCAT	C	AAGTCCGAAGAAGATCTCAA	0.997
atp4	atp4.t01!215	CCAGTCTATTCAGGAAGAAT	C	GCAGCAATTCCCCAATCCTA	0.997
atp4	atp4.t01!59	GCTATTTGCTGCTATTCCAT	C	TATTTGTGCATCAAGTCCGA	0.993
atp4	atp4.t01!118	ATGAAGAAATGATAGTAGCT	C	GTTGTTTTATAGGCTTTCTC	0.984
atp6	atp6.t01!68	GAAAAAAGGGGGGGGAAAGT	C	AGTGCCAAATGCTTGGCAAT	1
atp6	atp6.t01!106	AATCCTTGGTAGAGCTTATT	C	ATGATTTCGTGCCGAACCTG	1
atp6	atp6.t01!353	GCTTCATTTTTTTAGCTTCT	C	ATTACCCGCAGGAGTCCCAC	1
atp6	atp6.t01!415	TACTCCTTGAGCTAATCCCT	C	ATTGTTTTCGTGCATTAAGC	1
atp6	atp6.t01!479	TATGATGGCCGGTCATAGTT	C	AGTAAAGATTATAAGTGGGT	1

Name	pos	left	edit	right	score
atp6	atp6.t01!587	AGTTCTCGCATTAACCGGTC	C	GGAATTAGGTGTAGCTATAT	1
atp6	atp6.t01!632	AGCTCATGTTTCTACGATCT	C	AATCTGTATTTACTTGAATG	1
atp6	atp6.t01!119	GCTTATTCATGATTTCGTGC	C	GAACCTGGTAAACGAACAAA	0.999
atp6	atp6.t01!214	CTTTTACTTTTTCGTTATTT	C	GTAATCCCCAGGGTATGATA	0.999
atp6	atp6.t01!412	TAGTACTCCTTGAGCTAATC	C	CTCATTGTTTTCGTGCATTA	0.999
atp6	atp6.t01!437	TTGTTTTCGTGCATTAAGCT	C	AGGGATACGTTTATTTGCTA	0.999
atp6	atp6.t01!551	TATCTATTTCATAGGAGATC	C	TGGTCCTTTATTTATAGTTC	0.999
atp6	atp6.t01!659	TATTTACTTGAATGATGCTA	C	AAATCTCCATCAANNNNNNN	0.999
atp6	atp6.t01!206	CTCGGTCACTTTTACTTTTT	C	GTTATTTCGTAATCCCCAGG	0.998
atp6	atp6.t01!608	GGAATTAGGTGTAGCTATAT	C	ACAAGCTCATGTTTCTACGA	0.998
atp6	atp6.t01!181	TGAAACAAAAGTTTTCCCCT	C	GCATCTCGGTCACTTTTACT	0.997
atp6	atp6.t01!623	TATATCACAAGCTCATGTTT	C	TACGATCTCAATCTGTATTT	0.997
atp6	atp6.t01!176	AAATGTGAAACAAAAGTTTT	C	CCCTCGCATCTCGGTCACTT	0.993
atp6	atp6.t01!616	GTGTAGCTATATCACAAGCT	C	ATGTTTCTACGATCTCAATC	0.99
atp6	atp6.t01!221	TTTTTCGTTATTTCGTAATC	C	CCAGGGTATGATACCCTTTA	0.985
atp6	atp6.t01!188	AAAGTTTTCCCCTCGCATCT	C	GGTCACTTTTACTTTTTCGT	0.983
atp6	atp6.t01!222	TTTTCGTTATTTCGTAATCC	C	CAGGGTATGATACCCTTTAG	0.973
atp6	atp6.t01!670	ATGATGCTACAAATCTCCAT	C	AANNNNNNNNNNNNNNNNNN	0.936
atp8	atp8.t01!200	TAACAGTTTGGAAGATATCT	C	GAGAAAAGGTTTAGCACCG	1
atp8	atp8.t01!443	AATAATGCTCATCCATGTTC	C	ACACGGCCAAGGAAGCATCT	0.996
atp8	atp8.t01!469	GCCAAGGAAGCATCTTTTTT	C	GAGGGGAGTAANNNNNNNNN	0.993
atp9	atp9.t01!20	NATGTTAGAAGGTGCTAAAT	C	AATGGGTGCCGGAGCAGCTA	1
atp9	atp9.t01!50	CGGAGCAGCTACAATTGCTT	C	AGCGGGAGCTGCTGTCGGTA	0.999
atp9	atp9.t01!134	TCCATCATTGGCTAAACAAT	C	ATTTGGTTATGCAATTTTGG	0.999
atp9	atp9.t01!182	TCTCACCGAAGCTATTGCAT	C	GTTTGCCCCAATGATGGCGT	0.999
atp9	atp9.t01!205	TTGCCCCAATGATGGCGTCT	C	TGATCTCATTCGTCTTCCGA	0.999
atp9	atp9.t01!212	AATGATGGCGTCTCTGATCT	C	ATTCGTCTTCCGANNNNNNN	0.998

Name	pos	left	edit	right	score
atp9	atp9.t01!191	AGCTATTGCATCGTTTGCCC	C	AATGATGGCGTCTCTGATCT	0.995
atp9	atp9.t01!203	GTTTGCCCCAATGATGGCGT	C	TCTGATCTCATTCGTCTTCC	0.929
ccmB	ccmB.t01!286	TTCTACAATTGGTAGGTCAC	C	GGGTTATTCAAATAAGTCGT	0.999
ccmB	ccmB.t01!313	TTCAAATAAGTCGTGTTTTC	C	GTGGTTTTCCCATGTTACAA	0.999
ccmB	ccmB.t01!566	TCTTTCATCGATTGGTTATT	C	CTCCCCGTTCGTATCTCCTT	0.999
ccmB	ccmB.t01!128	AGGTTTTGAAAAAGACTTTT	C	ATGTCATTCCCATTTAGGTC	0.998
ccmB	ccmB.t01!137	AAAAGACTTTTCATGTCATT	C	CCATTTAGGTCTGATTCGGA	0.998
ccmB	ccmB.t01!475	GTTGGAACAGCTCGCAAAAT	C	CAACCACTTCACCTACTTCA	0.997
ccmB	ccmB.t01!304	ACCGGGTTATTCAAATAAGT	C	GTGTTTTCCGTGGTTTTCCC	0.996
ccmB	ccmB.t01!428	GTGTGGTATTCATTCTCGTT	C	GGCTCTTGGAATCACATCCA	0.996
ccmB	ccmB.t01!476	TTGGAACAGCTCGCAAAATC	C	AACCACTTCACCTACTTCAT	0.996
ccmB	ccmB.t01!194	CTTTCCTCCCGAACCTTTTC	C	TCGAAATGAGAAAGAAGATG	0.995
ccmB	ccmB.t01!551	AGAATGGTTTCATGTTCTTT	C	ATCGATTGGTTATTCCTCCC	0.995
ccmB	ccmB.t01!424	TTCTGTGTGGTATTCATTCT	C	GTTCGGCTCTTGGAATCACA	0.994
ccmB	ccmB.t01!503	TTCACCTACTTCATTGCCCC	C	AACCGTTTCCCGTACCTCTA	0.994
ccmB	ccmB.t01!494	TCCAACCACTTCACCTACTT	C	ATTGCCCCCAACCGTTTCCC	0.992
ccmB	ccmB.t01!172	TTCGGATCCCTCTGTTGTTT	C	CCTTTCCTCCCGAACCTTTT	0.989
ccmB	ccmB.t01!572	ATCGATTGGTTATTCCTCCC	C	GTTCGTATCTCCTTATCCCA	0.989
ccmB	ccmB.t01!154	ATTCCCATTTAGGTCTGATT	C	GGATCCCTCTGTTGTTTCCC	0.988
ccmB	ccmB.t01!380	AATGGATCGGTTAAACATTC	C	ATTAGGGAGCCCAGTCTTGA	0.986
ccmB	ccmB.t01!485	CTCGCAAAATCCAACCACTT	C	ACCTACTTCATTGCCCCCAA	0.985
ccmB	ccmB.t01!367	TCGATCGATCCGGAATGGAT	C	GGTTAAACATTCCATTAGGG	0.981
ccmB	ccmB.t01!554	ATGGTTTCATGTTCTTTCAT	C	GATTGGTTATTCCTCCCCGT	0.98
ccmB	ccmB.t01!467	CAGCAGTGGTTGGAACAGCT	C	GCAAAATCCAACCACTTCAC	0.975
ccmB	ccmB.t01!80	GAGTTTTTTTTTATTCCTAT	C	GTATATCGTCGTCACGCCCT	0.973
ccmB	ccmB.t01!338	TTTTCCCATGTTACAACTTT	C	GTACCAATTCGATCGATCCG	0.971
ccmB	ccmB.t01!512	TTCATTGCCCCCAACCGTTT	C	CCGTACCTCTATTGAAACAG	0.97

Name	pos	left	edit	right	score
ccmB	ccmB.t01!379	GAATGGATCGGTTAAACATT	C	CATTAGGGAGCCCAGTCTTG	0.967
ccmB	ccmB.t01!584	TTCCTCCCCGTTCGTATCTC	C	TTATCCCATTTTGGTCTCTA	0.962
ccmB	ccmB.t01!392	AAACATTCCATTAGGGAGCC	C	AGTCTTGACTCTTCTGTGTG	0.952
ccmB	ccmB.t01!611	CATTTTGGTCTCTATTAGTT	C	ACAAGATTGANNNNNNNNNN	0.949
ccmB	ccmB.t01!181	CTCTGTTGTTTCCCTTTCCT	C	CCGAACCTTTTCCTCGAAAT	0.937
ccmB	ccmB.t01!569	TTCATCGATTGGTTATTCCT	C	CCCGTTCGTATCTCCTTATC	0.92
ccmB	ccmB.t01!514	CATTGCCCCCAACCGTTTCC	C	GTACCTCTATTGAAACAGAA	0.916
ccmC	ccmC.t01!107	AACAGCGATGGCTATTCATT	C	AAGTCTTCGGGTAGCACCAC	1
ccmC	ccmC.t01!161	AGGTGGAAATTCTCGTATTC	C	GTATGTACATGTTCCTGCGG	1
ccmC	ccmC.t01!497	AGATATACCAATAATCAAGT	C	TTCAGTCAACTGGTGGAATA	1
ccmC	ccmC.t01!76	CGCAAATTCTAATTGGATCT	C	GGTTGTTCTTAACAGCGATG	0.999
ccmC	ccmC.t01!451	AGCTTCCTGTCGAACCGGCT	C	CTATTTTCATCCGTGCTGGA	0.999
ccmC	ccmC.t01!521	AGTCAACTGGTGGAATACAT	C	GCATCAACCTGGGAGCATTA	0.999
ccmC	ccmC.t01!184	ATGTACATGTTCCTGCGGCT	C	GGATGAGTATAGTAGTTTAT	0.998
ccmC	ccmC.t01!358	GGCCTATGTGGGGTACCTTT	C	GGGTGTGGGATGCTCGTTTC	0.998
ccmC	ccmC.t01!446	TCAAAAGCTTCCTGTCGAAC	C	GGCTCCTATTTTCATCCGTG	0.997
ccmC	ccmC.t01!331	CCTTAGTGACTGGGGGGTTT	C	GGGGAAGGCCTATGTGGGGT	0.996
ccmC	ccmC.t01!400	CTTCTGTATTCATCTTGTTC	C	TTATTTACCTGGGTGCACTT	0.996
ccmC	ccmC.t01!436	CACTTCGTTTTCAAAAGCTT	C	CTGTCGAACCGGCTCCTATT	0.996
ccmC	ccmC.t01!568	CTGGTACATCCATACATGTT	C	CTATGCCCATTCCAATCTTG	0.996
ccmC	ccmC.t01!236	TATAAACAGTTTCTTGTTCC	C	ATTAACAAAACATCCCCTTT	0.995
ccmC	ccmC.t01!656	GGAAACACGTCTTCCTATTC	C	ATCTTTTCTCGAATCTCCTT	0.995
ccmC	ccmC.t01!179	TCCGTATGTACATGTTCCTG	C	GGCTCGGATGAGTATAGTAG	0.994
ccmC	ccmC.t01!575	ATCCATACATGTTCCTATGC	C	CATTCCAATCTTGTCCAACT	0.993
ccmC	ccmC.t01!614	CTTTGCTAACTTCCCCTTCT	C	AACCCCTATCTTATTCGTTC	0.991
ccmC	ccmC.t01!399	ACTTCTGTATTCATCTTGTT	C	CTTATTTACCTGGGTGCACT	0.99
ccmC	ccmC.t01!650	CGTTCTGGAAACACGTCTTC	C	TATTCCATCTTTTCTCGAAT	0.99

Name	pos	left	edit	right	score
ccmC	ccmC.t01!103	TCTTAACAGCGATGGCTATT	C	ATTCAAGTCTTCGGGTAGCA	0.987
ccmC	ccmC.t01!115	TGGCTATTCATTCAAGTCTT	C	GGGTAGCACCACCAGATCTT	0.987
ccmC	ccmC.t01!591	ATGCCCATTCCAATCTTGTC	C	AACTTTGCTAACTTCCCCTT	0.987
ccmC	ccmC.t01!673	TTCCATCTTTTCTCGAATCT	C	CTTTCACTGAAGAAATAGAA	0.984
ccmC	ccmC.t01!421	TTATTTACCTGGGTGCACTT	C	GTTTTCAAAAGCTTCCTGTC	0.981
ccmC	ccmC.t01!608	GTCCAACTTTGCTAACTTCC	C	CTTCTCAACCCCTATCTTAT	0.977
ccmC	ccmC.t01!133	TTCGGGTAGCACCACCAGAT	C	TTCAACAAGGTGGAAATTCT	0.957
ccmC	ccmC.t01!630	TTCTCAACCCCTATCTTATT	C	GTTCTGGAAACACGTCTTCC	0.956
ccmC	ccmC.t01!548	ACCTGGGAGCATTAGCCGAT	C	TGGTACATCCATACATGTTC	0.943
ccmC	ccmC.t01!619	CTAACTTCCCCTTCTCAACC	C	CTATCTTATTCGTTCTGGAA	0.929
ccmC	ccmC.t01!281	TCGCTCTTCCGGAACCGGTA	C	AGAAATTGGTGCTTTTTCTA	0.903
ccmFC	ccmFC.t01!301	CTCAACGATTGCCTTTGGGT	C	CCGAACTCCATATGGGGAAG	1
ccmFC	ccmFC.t01!658	TATTTACGGATCTATATGCT	C	CGATTGGAACTGGAAGTTCC	0.999
ccmFC	ccmFC.t01!659	ATTTACGGATCTATATGCTC	C	GATTGGAACTGGAAGTTCCA	0.998
ccmFC	ccmFC.t01!814	AGGATAAGTTGCATTGGAAT	C	GANNNNNNNNNNNNNNNNNN	0.997
ccmFC	ccmFC.t01!758	AGGATTTCTGTTGGCTTCAT	C	GGGAGGCTCGCGTAGTTTGT	0.986
ccmFC	ccmFC.t01!767	GTTGGCTTCATCGGGAGGCT	C	GCGTAGTTTGTTACGTCAGC	0.981
ccmFC	ccmFC.t01!733	TGCCTTTTCTTTTTAGTATT	C	GGATAGGATTTCTGTTGGCT	0.978
ccmFN	ccmFN.t01!98	ACCAGCGTTTGGTGCAGCAC	C	TGCATTTTTGTGCATTCTTC	1
ccmFN	ccmFN.t01!1291	TGCCAGGAAGTTGGTGGGCT	C	ATCATGAATTAGGTCGGGGT	1
ccmFN	ccmFN.t01!1454	GACTCTTCCATGCTGTGTCT	C	AGGAACCTTTTCAATACGGT	1
ccmFN	ccmFN.t01!1502	GCTAGCTCCCGTTCATAGTT	C	TGCTACAGATGATACACGAG	1
ccmFN	ccmFN.t01!263	TTTCTCATGGTGTTGGATCC	C	AAGTTTTTATGGATTCCTTC	0.999
ccmFN	ccmFN.t01!680	CATTGCTTTGTTTTTCTCTC	C	TTTCCTATCAGCGAGTTCCG	0.999
ccmFN	ccmFN.t01!761	TGCAGAATCAAATCCTGTTC	C	ACAAGATCAATTATCAGCTA	0.999
ccmFN	ccmFN.t01!793	TATCAGCTATACATCCTCCT	C	GCATTTATGCCGGAGACGTC	0.999
ccmFN	ccmFN.t01!1274	TTTCACCGTAGGCATCTTGC	C	AGGAAGTTGGTGGGCTCATC	0.999

Name	pos	left	edit	right	score
ccmFN	ccmFN.t01!1306	GGGCTCATCATGAATTAGGT	C	GGGGTGGCTGGTGGTTTCGG	0.999
ccmFN	ccmFN.t01!1324	GTCGGGGTGGCTGGTGGTTT	C	GGGATCCCGTAGAAAATGCG	0.999
ccmFN	ccmFN.t01!1489	TACGGTCCGGATTGCTAGCT	C	CCGTTCATAGTTCTGCTACA	0.999
ccmFN	ccmFN.t01!248	TCATGAGGGTAGTATTTTCT	C	ATGGTGTTGGATCCCAAGTT	0.998
ccmFN	ccmFN.t01!727	TTGTTCGAAATTTCTTCGTT	C	GTACCGAACCGCTTGCAGAA	0.998
ccmFN	ccmFN.t01!1357	AAAATGCGTCTTTTATGCCT	C	GGGTATTAGCCACAGCTCGT	0.998
ccmFN	ccmFN.t01!38	CTCTCATTATTCGTTATTTC	C	GGGTCTTTTCGTTGCATTCA	0.997
ccmFN	ccmFN.t01!1375	CTCGGGTATTAGCCACAGCT	C	GTATTCATTCAGTAATTCTA	0.996
ccmFN	ccmFN.t01!283	CAAGTTTTTATGGATTCCTT	C	TTTGTTACCGGGGTCGACCC	0.995
ccmFN	ccmFN.t01!925	AAAAGAATGGAACGCTGCTT	C	GCTCTGCTGGATGCGTCGGA	0.993
ccmFN	ccmFN.t01!689	GTTTTTCTCTCCTTTCCTAT	C	AGCGAGTTCCGATCCTTTTG	0.991
ccmFN	ccmFN.t01!1420	TTCTTCATTCTTGGACCTCG	C	TTCTTAATACTTTGACTCTT	0.986
ccmFN	ccmFN.t01!1246	GAATTTGGATCTTGACATGT	C	GGTTATTTTTCACCGTAGGC	0.983
ccmFN	ccmFN.t01!749	TACCGAACCGCTTGCAGAAT	C	AAATCCTGTTCCACAAGATC	0.981
ccmFN	ccmFN.t01!137	TCTTCCTTTCCTTGGTCTTT	C	GTTCCGTCATCTTCCTAAAA	0.973
ccmFN	ccmFN.t01!121	CATTTTTGTGCATTCTTCTT	C	CTTTCCTTGGTCTTTCGTTC	0.972
ccmFN	ccmFN.t01!432	CCCCAGTTGTACACTTCCTT	C	GGAACCCTTGTGGATTCTGA	0.959
ccmFN	ccmFN.t01!1442	TCTTAATACTTTGACTCTTC	C	ATGCTGTGTCTCAGGAACCT	0.959
ccmFN	ccmFN.t01!1418	CCTTCTTCATTCTTGGACCT	C	GCTTCTTAATACTTTGACTC	0.948
ccmFN	ccmFN.t01!1537	CACGAGGAAGATTTTTATGG	C	GGTTCTTCATTATAATGACA	0.946
ccmFN	ccmFN.t01!142	CTTTCCTTGGTCTTTCGTTC	C	GTCATCTTCCTAAAAACTTA	0.935
ccmFN	ccmFN.t01!349	GAGGCTATAGAGAAACTTTT	C	CTAGCGCCTTTGTCTCGAAC	0.93
cob	cob.t01!419	AGCTTTTATAGGATACGTAC	C	ACCTTGGGGTCAGATGAGCT	1
cob	cob.t01!680	AGAGATGGATAAAATTGCTT	C	TTACCCTTATTTTTATGTAA	1
cob	cob.t01!808	ATATACCTGCTAATCCGATG	C	CCACCCCGCCTCATATTGTG	1
cob	cob.t01!178	GCGTTTTTTTAGCTATGCAT	C	ACACACCTCATGTGGATCTA	0.999
cob	cob.t01!286	CTAATGGGGCAAGTATGTTT	C	TCATTGTGGTTCACCTTCAT	0.999

Name	pos	left	edit	right	score
cob	cob.t01!298	GTATGTTTCTCATTGTGGTT	C	ACCTTCATCTTTTTCGTGGT	0.999
cob	cob.t01!725	TCTAGTAGGTCGGGTAGCTT	C	TGCTATCTTTTTTTCCATTT	0.999
cob	cob.t01!914	AGCCGCAATAGCACCAGTTT	C	TATATCTCTGTTGGCTTTAC	0.999
cob	cob.t01!1015	TTTGGTTGCTTTTGGCGGAT	C	GCTTACTACTAGGTTGGATC	0.999
cob	cob.t01!118	GTTATTGGTGGGGGTTCGGT	C	CGTTAGCTGGTCTTTGTTTA	0.998
cob	cob.t01!358	GCAGTCCTAGGGAATTGGTT	C	GGTGTCTCGGAGTTGTAATA	0.998
cob	cob.t01!715	ATGTAAAGGATCTAGTAGGT	C	GGGTAGCTTCTGCTATCTTT	0.998
cob	cob.t01!853	AATGGTATTTCCTACCGATC	C	ATGCCATTCTTCGCAGTATA	0.998
cob	cob.t01!908	AGGTGTAGCCGCAATAGCAC	C	AGTTTCTATATCTCTGTTGG	0.993
cob	cob.t01!982	GTTCAAGTTTTCGACCGATT	C	ACCAAGGAATATTTTGGTTG	0.986
cob	cob.t01!568	ATCGTTTTTTTAGTCTTCAT	C	ATTTACTCCCCTTTCTTTTA	0.98
cox1	cox1.t01!197	TTTAATAACGGCTCACGCTT	C	TTTAATGATCTTTTTTATGG	1
cox1	cox1.t01!254	AGGTGGATCTGGTAATTGGT	C	TGTTCCGATTCTGATAGGTG	1
cox1	cox1.t01!515	CAATTTTATAACAACTATCT	C	CAACATGCGTGGACCTGGAA	1
cox1	cox1.t01!551	TGGAATGACTATGCATAGAT	C	ACCCCTTTTTGTGTGGTCCG	1
cox1	cox1.t01!1186	TTGCTTTATTTGCAGGATTT	C	ACTATTGGGTGGGTAAAATC	1
cox1	cox1.t01!1259	AATCCATTTTTGGATCACTT	C	TTTCGGGGTTAATCCGACCC	1
cox1	cox1.t01!1405	TATCCGTAGTTGGGATTCGT	C	GTTTCTTCGTGGTCGTCACA	1
cox1	cox1.t01!452	AGCAGTTGATTCAGCAATTT	C	TAGTCCTCATCTATCAGGTG	0.999
cox1	cox1.t01!668	AAACTTTAATACAACCTTTT	C	TGATCCTGCTGGAGGGGGGG	0.999
cox1	cox1.t01!1037	CATGTTATTTGCTGTAGGGT	C	CATCTTTTTGTTCACCATAG	0.999
cox1	cox1.t01!1499	TGAACAGAATACAACCACAC	C	GGAATGGATGGTACAAAGTC	0.999
cox1	cox1.t01!242	GCCGGCGATGATAGGTGGAT	C	TGGTAATTGGTCTGTTCCGA	0.998
cox1	cox1.t01!352	GTCTCTTGCTCCTATTAAGC	C	CAGCCTTAGTAGAAGTGGGT	0.998
cox1	cox1.t01!458	TGATTCAGCAATTTCTAGTC	C	TCATCTATCAGGTGTTTCAT	0.998
cox1	cox1.t01!715	TATTATACCAGCATCTCTTT	C	GGTTCTTCGGTCATCCAGAG	0.997
cox1	cox1.t01!860	CAGTATAGGTGTTCCTGGAT	C	TCTTGTTCGGGCTCATCATA	0.997

Name	pos	left	edit	right	score
cox1	cox1.t01!1402	ATATATCCGTAGTTGGGATT	C	GTCGTTTCTTCGTGGTCGTC	0.997
cox1	cox1.t01!1446	ATCACTTCAAGCAGTGGAAA	C	AACAAAAGATGTGCTCCAAG	0.997
cox1	cox1.t01!299	TGACATGGCATTTCCACGAT	C	AAATAATATATCATTCTGGT	0.995
cox1	cox1.t01!746	TCATCCAGAGGTGTATATTC	C	AATTCTGCCTGGATCCGGTA	0.994
cox1	cox1.t01!443	TTCTGGAGGAGCAGTTGATT	C	AGCAATTTCTAGTCCTCATC	0.993
cox1	cox1.t01!1433	CGTGGTCGTCACAATCACTT	C	AAGCAGTGGAAACAACAAAA	0.989
cox1	cox1.t01!868	GTGTTCCTGGATCTCTTGTT	C	GGGCTCATCATATGTTTACT	0.988
cox1	cox1.t01!15	NNNNNNATGACAAATCTGGT	C	CGATGGCTGTTCTCCACTAA	0.984
cox1	cox1.t01!1279	CTTTCGGGGTTAATCCGACC	C	TCTTTCCCATGCATTTCTTA	0.98
cox1	cox1.t01!1274	CACTTCTTTCGGGGTTAATC	C	GACCCTCTTTCCCATGCATT	0.979
cox1	cox1.t01!854	CATGATCAGTATAGGTGTTC	C	TGGATCTCTTGTTCGGGCTC	0.978
cox1	cox1.t01!761	TATTCCAATTCTGCCTGGAT	C	CGGTATTATTAGTCATATCG	0.977
cox2	cox2.t01!83	GGAACCATGGCAATTAGGAT	C	TCAAGACGCAGCAACACCTA	1
cox2	cox2.t01!593	TAGTTGGGCTGTACCTTCCT	C	AGGTGTCAAATGTGATGCTG	1
cox2	cox2.t01!391	CTATTGGACATCAATGGTAT	C	GGACTTATGAGTATTCGGAC	0.999
cox2	cox2.t01!455	ACTCACTTTTGACAGTTATA	C	GATTCCAGAAGATGATCTAG	0.999
cox2	cox2.t01!556	ATCTACGTATGATTGTAACA	C	CTGCTGATGTACCTCATAGT	0.999
cox2	cox2.t01!688	ATGGTCAGTGCAGTGAGATT	C	GTGGAACTAATCATGCCTTT	0.999
cox2	cox2.t01!734	CTATCGTCGTAGAAGCTGTT	C	CTTTGAAAGATTATGGTTCT	0.999
cox2	cox2.t01!173	TCTTATTTTGGTTTTCGTAT	C	ACGGATGTTGGTTCGCGCTT	0.998
cox2	cox2.t01!175	TTATTTTGGTTTTCGTATCA	C	GGATGTTGGTTCGCGCTTTA	0.998
cox2	cox2.t01!626	TGATGCTGTACCTGGTCGTT	C	AAATCAGACCTCCATTTCGG	0.997
cox2	cox2.t01!569	TGTAACACCTGCTGATGTAC	C	TCATAGTTGGGCTGTACCTT	0.996
cox2	cox2.t01!488	TGATCTAGAATTAGGTCAAT	C	ACGTTTATTAGAAGTAGACA	0.995
cox2	cox2.t01!755	CTTTGAAAGATTATGGTTCT	C	GGGTATCCAATCAATTCCTC	0.991
cox2	cox2.t01!644	TTCAAATCAGACCTCCATTT	C	GGTACAACGAGAAGGAGTTT	0.99
cox2	cox2.t01!265	GAACTACTATCGAGATTCTT	C	GGACCATCTTTCCAAGTCTC	0.943

Name	pos	left	edit	right	score
cox3	cox3.t01!112	GAGGTGTGATGTACATGCAC	C	CATTTCAAGGGGGTGCAACA	1
cox3	cox3.t01!512	CGCTTTAGTAGCTACCGTTT	C	ACTGGCTCTAGTATTCACTG	1
cox3	cox3.t01!566	GGAATATTACCAAGCACCCT	C	CACTATTTCGGATAGTATTT	1
cox3	cox3.t01!754	GGCATTTTGTAGACGTGGTT	C	GGTTATTCCCATTTGTCTCT	1
cox3	cox3.t01!764	AGACGTGGTTCGGTTATTCC	C	ATTTGTCTCTATCTATTGGT	1
cox3	cox3.t01!289	TAGTCTCGGAGGTTATGTTC	C	TTTTTGCTTTTTTTTGGGCT	0.999
cox3	cox3.t01!311	TTTTGCTTTTTTTTGGGCTT	C	TTCTCATTCTTCTTTGGCAC	0.999
cox3	cox3.t01!314	TGCTTTTTTTTGGGCTTCTT	C	TCATTCTTCTTTGGCACCTA	0.999
cox3	cox3.t01!388	GGATTGGGGTTTTAGATCCT	C	GGGAAATCCCTTTTCTTAAT	0.999
cox3	cox3.t01!245	AAAAGCTGTACAATTAGGAC	C	TCGATACGGTTCTATTCTGT	0.998
cox3	cox3.t01!257	ATTAGGACCTCGATACGGTT	C	TATTCTGTTCATAGTCTCGG	0.992
cox3	cox3.t01!413	AATCCCTTTTCTTAATACTC	C	TATTCTCCCTTCATCCGGAG	0.992
cox3	cox3.t01!422	TCTTAATACTCCTATTCTCC	C	TTCATCCGGAGCTGCCGTAA	0.983
matR	matR.t01!1726	CTCTGTCCTACTACAGGTGC	C	GCGACAACCTTTACCAAGTC	1
matR	matR.t01!1070	ACGGAGTACGGTAGAATTCC	C	CGGTACGGTCATTCGGGAAG	0.999
matR	matR.t01!1706	CGCGGGCATCGCAATAAGTC	C	TCTGTCCTACTACAGGTGCC	0.998
matR	matR.t01!26	AGAAGCGATCAGAATGGTAC	C	CGAATCCATTTACGATCCCG	0.996
matR	matR.t01!1740	AGGTGCCGCGACAACCTTTA	C	CAAGTCCGAACGATTGTCGA	0.996
matR	matR.t01!1840	GGAATATAATCCCAAAGTAC	C	CAAAAGACTCAAATAGAGTA	0.995
matR	matR.t01!1832	TTCGGCGCGGAATATAATCC	C	AAAGTACCCAAAAGACTCAA	0.987
matR	matR.t01!1685	CGGAGACATAGTAAATTGGT	C	CGCGGGCATCGCAATAAGTC	0.985
matR	matR.t01!382	TAGGGAGGATCCGACAGAAG	C	ACGAAATTACTCTTGTTCTG	0.984
matR	matR.t01!1762	AAGTCCGAACGATTGTCGAC	C	ACCAGATCCGCTGGTCTGCT	0.983
matR	matR.t01!326	TGTACTACTATCGGCCCTAC	C	AGGCAACATTTACCTACACA	0.979
matR	matR.t01!32	GATCAGAATGGTACCCGAAT	C	CATTTACGATCCCGAGTTTC	0.908
mttB	mttB.t01!236	GTATTTTGTTCGTACACAAT	C	AACGGAGGCCTCCCCGACAT	1
mttB	mttB.t01!248	TACACAATCAACGGAGGCCT	C	CCCGACATATGTTGCAACGT	1

Name	pos	left	edit	right	score
mttB	mttB.t01!755	GATAATAGAGTTGGCTATCT	C	TGTGGCATTGATTGTACAAG	1
mttB	mttB.t01!148	TTGGTTTGACATGGTTTACG	C	GTTACTGGTTCCCGGAAGAG	0.999
mttB	mttB.t01!160	GGTTTACGCGTTACTGGTTC	C	CGGAAGAGTCAATTTCTCCA	0.999
mttB	mttB.t01!250	CACAATCAACGGAGGCCTCC	C	CGACATATGTTGCAACGTCT	0.999
mttB	mttB.t01!271	CGACATATGTTGCAACGTCT	C	CAATAGCATGCTCGTACTTC	0.999
mttB	mttB.t01!394	TCCTCCATTTAAGTGGTTCT	C	GCTTCTCCTTGTTCCTGTTC	0.999
mttB	mttB.t01!455	TCCCAATGTTTGGCACTTTC	C	ATACTTCGTGGGTGCAACAT	0.999
mttB	mttB.t01!170	TTACTGGTTCCCGGAAGAGT	C	AATTTCTCCATTAGCTAAAC	0.998
mttB	mttB.t01!226	CTTTGGACTCGTATTTTGTT	C	GTACACAATCAACGGAGGCC	0.998
mttB	mttB.t01!422	CTTGTTCCTGTTCCTAACTC	C	TCCCCGGGTAGTTCCCAATG	0.998
mttB	mttB.t01!329	TCATCAAATTTGGTGCTTTT	C	GATCCCCAGTTGCTATGGGG	0.997
mttB	mttB.t01!112	AAGTTCGAATCCGTTTCGTT	C	GGATATTGATCGGTCTTGGT	0.995
mttB	mttB.t01!427	TCCTGTTCCTAACTCCTCCC	C	GGGTAGTTCCCAATGTTTGG	0.995
mttB	mttB.t01!553	CTGTTCGTATTTCGTTCATT	C	CATCGGTATGCTCCCAGGTA	0.995
mttB	mttB.t01!725	CCAAATCGTCGCCCGTTTCC	C	TATTTATTTGATAATAGAGT	0.995
mttB	mttB.t01!661	GTCGTTTTTTTATGGTTTTT	C	CGCTTATCACAGCAGCTCTT	0.994
mttB	mttB.t01!520	TACAACCTAAGATCTATGAC	C	ATATTATGTTCACTGTTCGT	0.992
mttB	mttB.t01!424	TGTTCCTGTTCCTAACTCCT	C	CCCGGGTAGTTCCCAATGTT	0.99
mttB	mttB.t01!310	TCGTCTTTCCCTTAATAAGT	C	ATCAAATTTGGTGCTTTTCG	0.989
mttB	mttB.t01!376	GGACGAAATACAATCGATTC	C	TCCATTTAAGTGGTTCTCGC	0.984
mttB	mttB.t01!379	CGAAATACAATCGATTCCTC	C	ATTTAAGTGGTTCTCGCTTC	0.983
mttB	mttB.t01!723	TGCCAAATCGTCGCCCGTTT	C	CCTATTTATTTGATAATAGA	0.983
mttB	mttB.t01!545	TATGTTCACTGTTCGTATTT	C	GTTCATTCCATCGGTATGCT	0.968
mttB	mttB.t01!421	CCTTGTTCCTGTTCCTAACT	C	CTCCCCGGGTAGTTCCCAAT	0.948
mttB	mttB.t01!392	ATTCCTCCATTTAAGTGGTT	C	TCGCTTCTCCTTGTTCCTGT	0.933
mttB	mttB.t01!718	TCTGGTGCCAAATCGTCGCC	C	GTTTCCCTATTTATTTGATA	0.933
mttB	mttB.t01!179	CCCGGAAGAGTCAATTTCTC	C	ATTAGCTAAACCCTTTCTTA	0.92

Name	pos	left	edit	right	score
nad1	nad1.t01!215	ACCAAGTAGTGCTAATTTCT	C	CCTTTTTAGAATGGCTCCAG	1
nad1	nad1.t01!308	TGGTATGGTATTGTCAGATC	C	GAACATAGGGGTACTTTATT	1
nad1	nad1.t02!105	CTGTACTAATATGTGTAGGT	C	CCTGTAATTCGAGTGAGATT	1
nad1	nad1.t02!115	ATGTGTAGGTCCCTGTAATT	C	GAGTGAGATTGTCATGGCGC	1
nad1	nad1.t02!195	TTGTTATGTTCTTTATTTCT	C	GTCTAGCAGAAACTAATCGA	1
nad1	nad1.t02!223	AGAAACTAATCGAGCTCCGT	C	TGATCTCCCAGAAGCGGAAG	1
nad1	nad1.t02!289	TGAATATTCTTCAATGGGGT	C	TGCTCTTTTTTTTTGGGGG	1
nad1	nad1.t03!21	CGGTCCATGCACATCGCTCT	C	TCCAGGAGGTTGGCCGCCTA	1
nad1	nad1.t03!174	TTATGATCAATTAATGGGAC	C	TGGCCGGAAAGTGTTCTTGC	1
nad1	nad1.t01!265	TTATGTTAAGTCTGGTCGCT	C	GGGCCGTTGTACCTTTTGAT	0.999
nad1	nad1.t03!24	TCCATGCACATCGCTCTCTC	C	AGGAGGTTGGCCGCCTATCC	0.999
nad1	nad1.t03!179	ATCAATTAATGGGACCTGGC	C	GGAAAGTGTTCTTGCCTCTA	0.999
nad1	nad1.t03!209	TCTTGCCTCTATCATTAGCT	C	GGGTAGTCCCCGTTTCTGGT	0.999
nad1	nad1.t01!307	ATGGTATGGTATTGTCAGAT	C	CGAACATAGGGGTACTTTAT	0.998
nad1	nad1.t02!51	GATCTGCAGCTCAAATGGTC	C	CTTATGAAGTCTCTATTGGT	0.998
nad1	nad1.t02!250	CCCAGAAGCGGAAGCTGAAT	C	AGTTGCGGGCTATAATGTTG	0.998
nad1	nad1.t03!105	GTTTAGTATCAAGGTGATTC	C	TTTTCTTTTCCTATATATAT	0.997
nad1	nad1.t03!234	AGTCCCCGTTTCTGGTGTTT	C	AGTCACCTTTCGATGGCTCC	0.997
nad1	nad1.t03!36	GCTCTCTCCAGGAGGTTGGC	C	GCCTATCCTAGATCTTCCCA	0.995
nad1	nad1.t03!218	TATCATTAGCTCGGGTAGTC	C	CCGTTTCTGGTGTTTCAGTC	0.993
nad1	nad1.t02!107	GTACTAATATGTGTAGGTCC	C	TGTAATTCGAGTGAGATTGT	0.987
nad1	nad1.t03!60	TATCCTAGATCTTCCCATTT	C	TAATAAGATCCCGGGCTCGA	0.97
nad1	nad1.t02!151	GGCGCAAAAGCAGATATGGT	C	CGGTATTCCCTTATTCCCCG	0.968
nad1	nad1.t03!6	NNNNNNNNNNNNNNNCGGTC	C	ATGCACATCGCTCTCTCCAG	0.967
nad1	nad1.t02!152	GCGCAAAAGCAGATATGGTC	C	GGTATTCCCTTATTCCCCGT	0.963
nad1	nad1.t03!115	AAGGTGATTCCTTTTCTTTT	C	CTATATATATGGGTCCGTGC	0.961
nad1	nad1.t02!170	TCCGGTATTCCCTTATTCCC	C	GTATTTGTTATGTTCTTTAT	0.959

Name	pos	left	edit	right	score
nad1	nad1.t03!15	NNNNNNCGGTCCATGCACAT	C	GCTCTCTCCAGGAGGTTGGC	0.959
nad1	nad1.t01!376	ATGGAATTATTATAGCGGGT	C	GGTCTAGTANNNNNNNNNNN	0.906
nad2	nad2.t01!266	ATATTTCTGCCAAATCCTTC	C	ATTATTAAGTACGGCTGGTA	0.999
nad2	nad2.t01!422	CATGTATTTAGCTATTGAGC	C	TCAAAGTTTATGTTTTTATG	0.999
nad2	nad2.t01!491	ATTTTCCACGGAAGCCGGCT	C	GAAATATTTGATCTTAGGTG	0.999
nad2	nad2.t01!329	TTCCGAACAATTTGATGCTT	C	TGAATCCATTGTATTAATTC	0.997
nad2	nad2.t01!355	CCATTGTATTAATTCCACTT	C	CGACTCGCAGTATGCTCTTA	0.997
nad2	nad2.t01!388	TGCTCTTAATGATCTCGGCT	C	ATGATTCAATTGCCATGTAT	0.997
nad2	nad2.t01!395	AATGATCTCGGCTCATGATT	C	AATTGCCATGTATTTAGCTA	0.992
nad2	nad2.t01!335	ACAATTTGATGCTTCTGAAT	C	CATTGTATTAATTCCACTTC	0.983
nad2	nad2.t01!26	CAATCTTTTTTTAGCTGTTT	C	CCCAGAGATCTTTCTCATTA	0.98
nad2	nad2.t01!361	TATTAATTCCACTTCCGACT	C	GCAGTATGCTCTTAATGATC	0.97
nad2	nad2.t01!350	TGAATCCATTGTATTAATTC	C	ACTTCCGACTCGCAGTATGC	0.935
nad2	nad2.t01!252	AAGGACAATTTGACATATTT	C	TGCCAAATCCTTCCATTATT	0.926
nad3	nad3.t01!137	GTCGGCCCACGAATGTGGTT	C	CGATCCCTCCGGTGATGCCA	1
nad3	nad3.t01!209	TTCTATTTTCTTTATTATCC	C	TGATCCGGAAGTCACCTTTT	1
nad3	nad3.t01!266	ACCTCCCAACAAGATTGATC	C	GTTTGGATCTTGGTCCATGA	1
nad3	nad3.t01!317	CTTGATTTTGACGATTGGAT	C	TCTCTATGAATGGAAAAGGG	1
nad3	nad3.t01!185	TTTCGATATACGATTTTATC	C	GGTTTCTATTTTCTTTATTA	0.999
nad3	nad3.t01!344	TGAATGGAAAAGGGGTGCTT	C	GGATCGGGAGTAANNNNNNN	0.999
nad3	nad3.t01!349	GGAAAAGGGGTGCTTCGGAT	C	GGGAGTAANNNNNNNNNNNN	0.999
nad3	nad3.t01!80	CCCGCTCGGTCTTCCTTTTC	C	ATTTGCTTCCAATAGTTCGA	0.998
nad3	nad3.t01!124	ATCCAGAAAAATTGTCGGCC	C	ACGAATGTGGTTCCGATCCC	0.998
nad3	nad3.t01!146	CGAATGTGGTTCCGATCCCT	C	CGGTGATGCCAGAAGTCGTT	0.998
nad3	nad3.t01!275	CAAGATTGATCCGTTTGGAT	C	TTGGTCCATGATGGCCTTTT	0.998
nad3	nad3.t01!230	TGATCCGGAAGTCACCTTTT	C	TTCTCCTTGGGCAGTACCTC	0.997
nad3	nad3.t01!233	TCCGGAAGTCACCTTTTCTT	C	TCCTTGGGCAGTACCTCCCA	0.997

Name	pos	left	edit	right	score
nad3	nad3.t01!251	TTCTCCTTGGGCAGTACCTC	C	CAACAAGATTGATCCGTTTG	0.996
nad3	nad3.t01!62	TCTGCTAGTTTCTTTGATCC	C	GCTCGGTCTTCCTTTTCCAT	0.991
nad3	nad3.t01!208	TTTCTATTTTCTTTATTATC	C	CTGATCCGGAAGTCACCTTT	0.991
nad3	nad3.t01!247	TTTCTTCTCCTTGGGCAGTA	C	CTCCCAACAAGATTGATCCG	0.991
nad3	nad3.t01!61	GTCTGCTAGTTTCTTTGATC	C	CGCTCGGTCTTCCTTTTCCA	0.939
nad3	nad3.t01!39	ATTTTGATCTATTTAGTGAT	C	AGTCTGCTAGTTTCTTTGAT	0.936
nad3	nad3.t01!215	TTTCTTTATTATCCCTGATC	C	GGAAGTCACCTTTTCTTCTC	0.928
nad4	nad4.t01!50	CGATCTAAGTGGTCTTATTC	C	GTGTCCCGTGCTAGGAAGCA	1
nad4	nad4.t01!166	TTTTGTATCCCCCTGTTCCT	C	GGATACAATTCGATCCTTCT	1
nad4	nad4.t01!401	ATTTCTAATGATCGCCGTGT	C	CTGCATGCTGGATCCTCTAC	1
nad4	nad4.t01!857	CACTCCTTTCATTTATACTC	C	AAGCGCGATTGCTATAATAT	1
nad4	nad4.t01!883	CGATTGCTATAATATATACT	C	CCTCGACCACTTCAAGACAG	1
nad4	nad4.t01!934	AGAAGATCATTGCCTACTCT	C	CAGTAGCCCATATGAATCTG	1
nad4	nad4.t01!971	TCTGGTGACTATTGGTATGT	C	TAGTCCGAACATACAGGGAA	1
nad4	nad4.t01!977	GACTATTGGTATGTCTAGTC	C	GAACATACAGGGAATTGGAG	1
nad4	nad4.t01!1010	AATTGGAGGTAGCATTCCAC	C	GATGTCAAGTCATGGACTGG	1
nad4	nad4.t01!1016	AGGTAGCATTCCACCGATGT	C	AAGTCATGGACTGGTTCCTC	1
nad4	nad4.t01!1109	TGTTAGATATTATGGAGGTT	C	AGTGAGCACCATGCCGAATC	1
nad4	nad4.t01!1172	CACTTTGGCCAATATGAGTT	C	ACCTGGTACTAGCAGCTTTA	1
nad4	nad4.t01!29	ACATTTCTTTGAATGCTATT	C	CGATCTAAGTGGTCTTATTC	0.999
nad4	nad4.t01!77	CGTGCTAGGAAGCATTACTC	C	TCTTTTCATTCCAAATTCAA	0.999
nad4	nad4.t01!107	TCCAAATTCAAGAATACGAC	C	GATACGATTGATTGGTCTGT	0.999
nad4	nad4.t01!154	CTCTTATTACTTTTTTGTAT	C	CCCCTGTTCCTCGGATACAA	0.999
nad4	nad4.t01!433	ATCCTCTACTATTCTATGTT	C	TTCCCGAAAGCGTGCCAATC	0.999
nad4	nad4.t01!436	CTCTACTATTCTATGTTCTT	C	CCGAAAGCGTGCCAATCCCT	0.999
nad4	nad4.t01!566	TTTTATGCTATTAGCTATTC	C	GTTGATTCTTCTCCAAACAG	0.999
nad4	nad4.t01!599	CCAAACAGGAACCACCGATT	C	ACAAATCTTATTAACCACAG	0.999

Name	pos	left	edit	right	score
nad4	nad4.t01!659	AATCTTTCTATGGATTGCTT	C	TTTCGCCTCTTTCGCCGTCA	0.999
nad4	nad4.t01!767	CGTCATCTTGGCAGGAATTC	C	TTTAAAATTGGGAACCTACG	0.999
nad4	nad4.t01!832	TGTTTCCCGAAGCGACACTT	C	GTTCCACTCCTTTCATTTAT	0.999
nad4	nad4.t01!1211	TATAGGGGAATTTCCAATCT	C	AGTAGGAGCTTTCCAAAGAA	0.999
nad4	nad4.t01!1405	CTTTTCTTGTTGGAGTTGTT	C	GGATGGGTGTTCACCCCAAA	0.999
nad4	nad4.t01!1417	GAGTTGTTCGGATGGGTGTT	C	ACCCCAAAGTGTTCCCGGAC	0.999
nad4	nad4.t01!197	CGATCCTTCTACGGCCAAAT	C	TCAATTTGTGGAAAGCCTTC	0.998
nad4	nad4.t01!317	TCTTATCCCTATTTGCATTT	C	AGTGGGTTGGTCTGGTATGA	0.998
nad4	nad4.t01!416	CGTGTCCTGCATGCTGGATC	C	TCTACTATTCTATGTTCTTC	0.998
nad4	nad4.t01!1132	TGAGCACCATGCCGAATCTC	C	CTACCATTTCCTTCTCTTCC	0.998
nad4	nad4.t01!1148	TCTCCCTACCATTTCCTTCT	C	TTCCACTTTGGCCAATATGA	0.998
nad4	nad4.t01!1373	TCCAAATGGCAGAGAAGTTT	C	CATATTTCTACCTTTTCTTG	0.998
nad4	nad4.t01!1433	TGTTCACCCCAAAGTGTTCC	C	GGACTGCATGCATACATCCG	0.998
nad4	nad4.t01!74	TCCCGTGCTAGGAAGCATTA	C	TCCTCTTTTCATTCCAAATT	0.997
nad4	nad4.t01!158	TATTACTTTTTTGTATCCCC	C	TGTTCCTCGGATACAATTCG	0.997
nad4	nad4.t01!896	ATATACTCCCTCGACCACTT	C	AAGACAGATCGATCTTAAGA	0.997
nad4	nad4.t01!1033	TGTCAAGTCATGGACTGGTT	C	CTCCAGCCCTCTTTCTATGT	0.997
nad4	nad4.t01!368	GAAAGAGTATATTACAGCAT	C	TCTAATTCGTGAATTTCTAA	0.996
nad4	nad4.t01!376	ATATTACAGCATCTCTAATT	C	GTGAATTTCTAATGATCGCC	0.995
nad4	nad4.t01!449	TGTTCTTCCCGAAAGCGTGC	C	AATCCCTATGTTCATTATTA	0.995
nad4	nad4.t01!887	TGCTATAATATATACTCCCT	C	GACCACTTCAAGACAGATCG	0.995
nad4	nad4.t01!1129	CAGTGAGCACCATGCCGAAT	C	TCCCTACCATTTCCTTCTCT	0.995
nad4	nad4.t01!1355	CCTCCATAAATTCTCCGATC	C	AAATGGCAGAGAAGTTTCCA	0.995
nad4	nad4.t01!164	TTTTTTGTATCCCCCTGTTC	C	TCGGATACAATTCGATCCTT	0.994
nad4	nad4.t01!362	TTTTGGGAAAGAGTATATTA	C	AGCATCTCTAATTCGTGAAT	0.994
nad4	nad4.t01!1036	CAAGTCATGGACTGGTTCCT	C	CAGCCCTCTTTCTATGTGTT	0.994
nad4	nad4.t01!1307	CCTTTGGCTATATAATCGTG	C	GGTTTCTGGGAATTTCAAAC	0.993

Name	pos	left	edit	right	score
nad4	nad4.t01!437	TCTACTATTCTATGTTCTTC	C	CGAAAGCGTGCCAATCCCTA	0.992
nad4	nad4.t01!836	TCCCGAAGCGACACTTCGTT	C	CACTCCTTTCATTTATACTC	0.992
nad4	nad4.t01!1007	GGGAATTGGAGGTAGCATTC	C	ACCGATGTCAAGTCATGGAC	0.992
nad4	nad4.t01!1151	CCCTACCATTTCCTTCTCTT	C	CACTTTGGCCAATATGAGTT	0.992
nad4	nad4.t01!1142	GCCGAATCTCCCTACCATTT	C	CTTCTCTTCCACTTTGGCCA	0.99
nad4	nad4.t01!533	TCAGTTTTTCCTATATACTT	C	ACTTGGATCTGTTTTTATGC	0.986
nad4	nad4.t01!1006	AGGGAATTGGAGGTAGCATT	C	CACCGATGTCAAGTCATGGA	0.986
nad4	nad4.t01!856	CCACTCCTTTCATTTATACT	C	CAAGCGCGATTGCTATAATA	0.979
nad4	nad4.t01!1205	CAGCTTTATAGGGGAATTTC	C	AATCTCAGTAGGAGCTTTCC	0.979
nad4	nad4.t01!1323	CGTGCGGTTTCTGGGAATTT	C	AAACCCGATTTCCTCCATAA	0.956
nad4	nad4.t01!30	CATTTCTTTGAATGCTATTC	C	GATCTAAGTGGTCTTATTCC	0.94
nad4L	nad4L.t01!41	CACATTTTCTATGATCATCT	C	TATTTTAGGTATTCGGGGAA	1
nad4L	nad4L.t01!95	ACGAAATATTCCTATTATGT	C	AATGTCAATTGAATCAATGT	1
nad4L	nad4L.t01!110	TATGTCAATGTCAATTGAAT	C	AATGTTATTAGCTGTCAATT	1
nad4L	nad4L.t01!131	AATGTTATTAGCTGTCAATT	C	GAACTTTTTGGTCTTTTCCG	1
nad4L	nad4L.t01!179	GGATGATATGATGGGTCAAT	C	ATTTGCTTCATTAGTTCCAA	1
nad4L	nad4L.t01!86	TCTTAATAGACGAAATATTC	C	TATTATGTCAATGTCAATTG	0.997
nad4L	nad4L.t01!188	GATGGGTCAATCATTTGCTT	C	ATTAGTTCCAACAGTGGCAG	0.997
nad4L	nad4L.t01!281	AGGGACTATTGCTGTGGAAT	C	TATAAATTGCATTCAAGGTT	0.997
nad4L	nad4L.t01!197	ATCATTTGCTTCATTAGTTC	C	AACAGTGGCAGCTGCGGAAT	0.996
nad4L	nad4L.t01!55	TCATCTCTATTTTAGGTATT	C	GGGGAATCCTTCTTAATAGA	0.994
nad4L	nad4L.t01!230	TGCGGAATCTGCTATTGGAT	C	AGCCATTTTCGTTCTTACTT	0.99
nad5	nad5.t01!22	CAATTTTTGGGCCAATTCCC	C	CTTCGTACTACCAAAAAATG	0.999
nad5	nad5.t01!448	AACTCTATTTGTGACCTTTT	C	TCGTATGTGGGACTCTATAT	0.999
nad5	nad5.t01!112	ACCTATTCTTTTTAGTACTT	C	AGGTGCTTCTATAGCGTATA	0.996
nad5	nad5.t01!427	CTTTGCAATCTTACTTGGTT	C	AACTCTATTTGTGACCTTTT	0.995
nad5	nad5.t01!450	CTCTATTTGTGACCTTTTCT	C	GTATGTGGGACTCTATATCT	0.995

Name	pos	left	edit	right	score
nad5	nad5.t01!142	TATAGCGTATAATGTCAATC	C	CGTAGCGGATCAATTCCAAC	0.968
nad5	nad5.t01!121	TTTTAGTACTTCAGGTGCTT	C	TATAGCGTATAATGTCAATC	0.938
nad5	nad5.t01!82	GTTTGCTGCTCCAACCATTA	C	AAAACTCATACCTATTCTTT	0.921
nad6	nad6.t01!191	TATAGGAGCTATTGCCGTTT	C	ATTCCTATTCGTGGTTATGA	1
nad6	nad6.t01!379	CCTCTCTGAGATATACGGTT	C	ATGCCGGAAAGGTACGAAGT	1
nad6	nad6.t01!26	ACTTTCTGTTTCGTCGAGCC	C	TGCTTTGGTCTCTGGTTTGA	0.999
nad6	nad6.t01!83	AAATCCGGTACATTCCGTTT	C	GTTTCCCATCCCAGTCTTTC	0.998
nad6	nad6.t01!158	CGACTTCTCCGCTATGATCT	C	CCCAGTAGTTCATATAGGAG	0.998
nad6	nad6.t01!169	CTATGATCTCCCCAGTAGTT	C	ATATAGGAGCTATTGCCGTT	0.998
nad6	nad6.t01!569	CCGACGAAATGCGTTGGATT	C	TAGGAGGACTATAATGAGGA	0.998
nad6	nad6.t01!446	TTTACTGTATACCTACTATT	C	TGTCTGGTTTTTGGTTCCTA	0.997
nad6	nad6.t01!89	GGTACATTCCGTTTCGTTTC	C	CATCCCAGTCTTTCGCGACA	0.996
nad6	nad6.t01!161	CTTCTCCGCTATGATCTCCC	C	AGTAGTTCATATAGGAGCTA	0.996
nad6	nad6.t01!463	ATTCTGTCTGGTTTTTGGTT	C	CTAGTCTGATTTTATTAGTA	0.996
nad6	nad6.t01!17	NNNNATGATACTTTCTGTTT	C	GTCGAGCCCTGCTTTGGTCT	0.995
nad6	nad6.t01!103	CGTTTCCCATCCCAGTCTTT	C	GCGACACTGCTGGTTTACTT	0.994
nad6	nad6.t01!95	TTCCGTTTCGTTTCCCATCC	C	AGTCTTTCGCGACACTGCTG	0.991
nad6	nad6.t01!146	TTTCTTAGGTCTCGACTTCT	C	CGCTATGATCTCCCCAGTAG	0.991
nad6	nad6.t01!88	CGGTACATTCCGTTTCGTTT	C	CCATCCCAGTCTTTCGCGAC	0.981
nad7	nad7.t01!44	CAAGAATTTCACTTTGAATT	C	CGGACCTCAACATCCTGCTG	1
nad7	nad7.t01!224	TCGTTCAGACTATGTTTCTA	C	GATGGCCCAAGAACACGCTC	1
nad7	nad7.t01!383	TGCTATGGATGTGGGAGCAT	C	AACTCCGTTCCTTTGGGCTT	1
nad7	nad7.t01!445	TGGAATTCTATGAAAGAGTC	C	CGGGAGCCAGGATGCATGCC	1
nad7	nad7.t01!698	TCCTGGGGTATGCTGGGATT	C	GCGAAGAGCAGCACCTTACG	1
nad7	nad7.t01!739	ATGTTTATGACCAATTGGAT	C	CTGACGTACCAGTAGGTACC	1
nad7	nad7.t01!769	CAGTAGGTACCAGAGGAGAT	C	GCTATGATCGTTACTGTATC	1
nad7	nad7.t01!1103	CCATTCACAAGGACTCGATT	C	TATGTCTAAACATCACATGC	1

Name	pos	left	edit	right	score
nad7	nad7.t01!83	TGCTCATGGTGTTTCACGAT	C	AGTATTGGAAATGAATGGAG	0.999
nad7	nad7.t01!244	CGATGGCCCAAGAACACGCT	C	ATTCTTCAGCCGTAGAGAGA	0.999
nad7	nad7.t01!344	AACTCGAATTTCAAATCATT	C	ACTTGCTTCAACTACTCATG	0.999
nad7	nad7.t01!353	TTCAAATCATTCACTTGCTT	C	AACTACTCATGCTATGGATG	0.999
nad7	nad7.t01!740	TGTTTATGACCAATTGGATC	C	TGACGTACCAGTAGGTACCA	0.999
nad7	nad7.t01!836	TCGGATCATTGTGCAATGTC	C	TAATCAAATGCCTAGTGGCA	0.999
nad7	nad7.t01!926	GAAACTATCCATGGAATCCT	C	AATTCACCATTTCGAACTTT	0.999
nad7	nad7.t01!973	AAGGTTTTTCCGTACCAGCT	C	CTTCTACCTATACCGCAGTT	0.999
nad7	nad7.t01!1079	TAAAATAAGAGCACCTGGCT	C	TGCCCATTCACAAGGACTCG	0.999
nad7	nad7.t01!335	CCGTGAAATAACTCGAATTT	C	AAATCATTCACTTGCTTCAA	0.998
nad7	nad7.t01!533	ATGTCGAGATATTGATTCCT	C	CACACAACAATTTGCTTCTC	0.998
nad7	nad7.t01!1057	GAAGCAATCGTCCCTACCGT	C	GTAAAATAAGAGCACCTGGC	0.998
nad7	nad7.t01!1124	TATGTCTAAACATCACATGC	C	AGCAGATGTAGTCACCATCA	0.998
nad7	nad7.t01!316	AATATATACGAGTGTTATTC	C	GTGAAATAACTCGAATTTCA	0.997
nad7	nad7.t01!578	CGACGAATTAGAAGAGATGC	C	AACTGGCAACCGTATCTGGA	0.997
nad7	nad7.t01!679	GTGGTGTAATGTTAAGAGGT	C	CTGGGGTATGCTGGGATTCG	0.997
nad7	nad7.t01!1088	AGCACCTGGCTCTGCCCATT	C	ACAAGGACTCGATTCTATGT	0.996
nad7	nad7.t01!200	TCTTCAAGCTTTACCTTATT	C	TGATCGTTCAGACTATGTTT	0.995
nad7	nad7.t01!45	AAGAATTTCACTTTGAATTC	C	GGACCTCAACATCCTGCTGC	0.993
nad7	nad7.t01!251	CCAAGAACACGCTCATTCTT	C	AGCCGTAGAGAGACTTTTGA	0.993
nad7	nad7.t01!77	TCCTGCTGCTCATGGTGTTT	C	ACGATCAGTATTGGAAATGA	0.991
nad7	nad7.t01!209	TTTACCTTATTCTGATCGTT	C	AGACTATGTTTCTACGATGG	0.989
nad7	nad7.t01!1050	AGTAATGGAAGCAATCGTCC	C	TACCGTCGTAAAATAAGAGC	0.966
nad7	nad7.t01!789	CGCTATGATCGTTACTGTAT	C	CGTATCGAAGAGATGCGACA	0.922
nad9	nad9.t01!190	TCGATATTTGCGGAGTTGAT	C	ATCCCTCTCGAAAACGAAGA	1
nad9	nad9.t01!311	AATATCTCCGGTAGTCAGTC	C	ATTTCCATCAGCCGGCCGGT	1
nad9	nad9.t01!539	AGAATTTCGCTATTTCGATT	C	TGCTAGTCCTTGGGAACAGC	1

Name	pos	left	edit	right	score
nad9	nad9.t01!406	TACGCCGTATATCAACAGAT	C	ATGGTTTCGAAGGTCATCCA	0.999
nad9	nad9.t01!92	ATCGGAACATGGGAATAGAT	C	TGATACCAATACTGACTACC	0.998
nad9	nad9.t01!328	GTCCATTTCCATCAGCCGGC	C	GGTGGGAGCGAGAAGTATGG	0.998
nad9	nad9.t01!368	GGATATGTCTGGTGTTTCTT	C	CATCAATCATCCGGATTTAC	0.998
nad9	nad9.t01!14	NNNNNNNATGGATAACCAAT	C	CATTTTCAAATATAGTTGGG	0.996
nad9	nad9.t01!298	ACGAAGTCACACGAATATCT	C	CGGTAGTCAGTCCATTTCCA	0.996
nad9	nad9.t01!167	CTATACACGGGTTCAAGTTT	C	GATCGATATTTGCGGAGTTG	0.992
nad9	nad9.t01!356	GCGAGAAGTATGGGATATGT	C	TGGTGTTTCTTCCATCAATC	0.99
nad9	nad9.t01!113	TGATACCAATACTGACTACC	C	ATTTCCATTGTTGTGCTTTC	0.989
nad9	nad9.t01!398	TCCGGATTTACGCCGTATAT	C	AACAGATCATGGTTTCGAAG	0.967
nad9	nad9.t01!223	AACGAAGATTTTCAGTTGTC	C	ATAATTTACTGAGTACTCGG	0.965
rpl5	rpl5.t01!47	AGATGTATCACGTCAGGATC	C	GTTGCTCAAACCGAATCACG	0.998
rpl5	rpl5.t01!59	TCAGGATCCGTTGCTCAAAC	C	GAATCACGCCAACGTTATGG	0.998
rpl5	rpl5.t01!92	CGTTATGGAAGTTCCTGGAT	C	GTGTGAAATCAGATTAGTAC	0.998
rpl5	rpl5.t01!370	CGGTCGAAATAAGGGAAAAC	C	CCATTTTTGTTTCGATGGAA	0.993
rpl5	rpl5.t01!506	CACAAAAGATGAGACTTTAC	C	ACCGTGGAGCGGCTTTTTGC	0.992
rpl5	rpl5.t01!35	TTTTCATTACGAAGATGTAT	C	ACGTCAGGATCCGTTGCTCA	0.99
rpl5	rpl5.t01!509	AAAAGATGAGACTTTACCAC	C	GTGGAGCGGCTTTTTGCTAA	0.97
rpl5	rpl5.t01!126	TTAGTACCAAACTCTGATTT	C	AGAATCCTATTTGGAAAATT	0.957
rpl5	rpl5.t01!64	ATCCGTTGCTCAAACCGAAT	C	ACGCCAACGTTATGGAAGTT	0.94
rps1	rps1.t01!99	TCAGTTTTACGCTTACGCTT	C	AGGGAAGAAATGTTCTTGGT	0.982
rps1	rps1.t01!215	GTTTGAGAATAAGGTGGGAT	C	CTTGAATGTAGTGGCTGGTG	0.974
rps1	rps1.t01!131	GTTCTTGGTGGATGCAGGAC	C	TGGTACCCCAAAAATTTGTA	0.967
rps1	rps1.t01!164	AATTTGTATGCAAGATGAGC	C	TACAGGAGTGCCAATCCACC	0.955
rps1	rps1.t01!71	ATTATGTAGTGGAAACGCCT	C	ACAATCTTCAGTTTTACGCT	0.933
rps1	rps1.t01!23	GAGCATCTATTGGAGTCGAT	C	ATTTCCAAGATCTAATTCCA	0.927
rps1	rps1.t01!593	GAGCATTAACCCCAAAAGGA	C	GAATATTGTGGTGTTCTAAN	0.912

Name	pos	left	edit	right	score
rps10	rps10.t01!232	AATTGCGCAAGAAGTTCTTT	C	GGTTAAAACGCCAGCGTATA	1
rps10	rps10.t01!296	TCTTTCTTGCAAGACCCGTT	C	GGATAAGGGCAAACTCCAGA	0.991
rps11	rps11.t01!161	TAACTTCGTACAGAGCATCT	C	GGAGGAAGATGAGCAATGTT	0.939
rps11	rps11.t01!552	CATAATGGATGCCGACTCCC	C	AAAAAACGTCGTGTTCAANN	0.929
rps12	rps12.t01!104	CCAGAAGCAAGGAGTATGCC	C	GCGTGTTTCGACGAGAACAC	1
rps12	rps12.t01!232	GTCATAATTCGCAGGAACAT	C	CTATAGTCTTAGTCAGAGGA	1
rps12	rps12.t01!196	ATCGACATGATATATTTGCT	C	ACATTCCAGGCGAAGGTCAT	0.999
rps12	rps12.t01!221	TCCAGGCGAAGGTCATAATT	C	GCAGGAACATCCTATAGTCT	0.998
rps12	rps12.t01!284	AGATTCGCCAGGTGTGAAAT	C	CCATTGTATTCGGGGAGTCA	0.998
rps12	rps12.t01!269	AGGAGGTAGAGTGAAAGATT	C	GCCAGGTGTGAAATCCCATT	0.997
rps13	rps13.t01!256	AACGATTAATTTCTATTTCT	C	GTTATCGTGGAATTCGTCAT	0.998
rps13	rps13.t01!26	ATATATCTCAGGAGCTAGAT	C	AGTTCCCGATGAACAAGTCA	0.995
rps13	rps13.t01!100	CTAAAAAAGCCATTCAGGTT	C	GTTATCGATTAGGTATCAGT	0.993
rps13	rps13.t01!287	AATTCGTCATCAAGATGGAT	C	GCCCTTACGCGGCCAACGAA	0.993
rps13	rps13.t01!56	TGAACAAGTCAGAATTGCCT	C	AACAAAAATGGATGGAATTG	0.989
rps2	rps2.t01!547	TACAGTTCGTATATCTATTT	C	GTCATTCGATCACGAAAACA	0.976
rps2	rps2.t01!182	TTTTCATTTTATAGGATCTC	C	CATTCGTCAAAAAGGCCGTT	0.958
rps2	rps2.t01!523	ATCCCATTCCAGCGAATGCT	C	CTATACAGTTCGTATATCTA	0.956
rps2	rps2.t01!452	TCGATCACAAATACCTATTG	C	ATCCTTAGTGGATTCTACGA	0.94
rps4	rps4.t01!184	CACAAACTACACGAAAGTTG	C	CCCTTTCTTATGGGGATTTA	1
rps4	rps4.t01!941	AACACTAAAAGCTGTGGTAT	C	TTATGGACCTCACATAGGTC	0.999
rps4	rps4.t01!338	TCTTCCTCAAGCAAGGCAGC	C	GATAAGTCATCGAAGGGTTT	0.997
rps4	rps4.t01!1001	ATTGAAAGATCCAAACCTTC	C	TCTTCGGAGCGGAAACGGAC	0.996
rps4	rps4.t01!191	TACACGAAAGTTGCCCCTTT	C	TTATGGGGATTTACCCATAA	0.995
rps4	rps4.t01!281	ACTCAATCAAGAAACAAGAT	C	GGACGTGATTCCGGTTCGTC	0.994
rps4	rps4.t01!49	GTCTACTTCCAGGAAATGTT	C	GGAACAGAGAACTTTCTCTA	0.993
rps4	rps4.t01!1006	AAGATCCAAACCTTCCTCTT	C	GGAGCGGAAACGGACGTGAC	0.992

Name	pos	left	edit	right	score
rps4	rps4.t01!38	TCAAACGTGTCGTCTACTTC	C	AGGAAATGTTCGGAACAGAG	0.986
rps4	rps4.t01!905	AGAACTACCTACTCATTATT	C	GGAGGTGAATCATAAAACAC	0.974
rps4	rps4.t01!992	CGACATCAGATTGAAAGATC	C	AAACCTTCCTCTTCGGAGCG	0.95
rps4	rps4.t01!485	ATCTTTCTATATCGACATAT	C	AGTTTCAAAAAGAATAGGAA	0.922
rps4	rps4.t01!164	TCTAAACAGTAACATCAAAT	C	ACAAACTACACGAAAGTTGC	0.915
rps4	rps4.t01!837	TCTTTATATAGTAATTCGAC	C	TATTGCTCCGGATCCCCTTT	0.901
rps7	rps7.t01!332	GATAAGCTTAGAGAAATGTT	C	ATTTGCTGAGATACTGGATG	0.996
rps7	rps7.t01!116	TTATCAAACTTTTCATCGCC	C	AGCTCGAACTGAACGCGATG	0.992
sdh4	sdh4.t01!29	GGCATTTTGTAGACGTGGTT	C	GGTTATTCCCATTTGTCTCT	1
sdh4	sdh4.t01!39	AGACGTGGTTCGGTTATTCC	C	ATTTGTCTCTATCTATTGGT	1

Table S7. The identification MTPTs in *D. officinale* mitogenomes.

Number	Identity(%)	Alignment Length(bp)	Chloroplast Genome Start	Chloroplast Genome End	Mitochondrial Genome Start	Mitochondrial Genome End	MTPT Annotation
MTPT1	96.494	4735	81551	86265	1	4684	complete ycf3; partial psaA; partial trnS
MTPT2	99.929	4220	63978	68197	4667	448	complete rpoB; complete rpoC1
MTPT3	93.102	3233	97412	100619	8102	4957	complete accD; complete psaI; partial ycf4
MTPT4	94.942	3203	146145	149331	25338	22240	partial rps19; partial ycf2_Ycf2
MTPT5	94.942	3203	17079	20265	22240	25338	partial trnI_tRNA-Ile; partial ycf2_Ycf2
MTPT6	98.944	3126	35739	38849	15635	12518	complete rrn4.5S; partial rrn23S
MTPT7	98.944	3126	127561	130671	12518	15635	complete rrn4.5S; partial rrn23S
MTPT8	99.928	2769	36081	38849	39700	36932	complete rrn4.5S; partial rrn23S
MTPT9	99.928	2769	127561	130329	36932	39700	complete rrn4.5S; partial rrn23S
MTPT10	96.947	2653	59660	62298	14982	12356	partial rpoC1; partial rpoC2
MTPT11	95.355	2562	7904	10444	21053	18545	complete ndhD; complete ndhE; complete psaC
MTPT12	93.823	2477	113392	115820	43317	40855	complete psbB; complete psbT
MTPT13	96.084	2298	92217	94502	12731	10480	complete atpB; complete atpE; partial trnM
MTPT14	93.401	2061	101736	103787	15879	13911	complete petA; partial cemA
MTPT15	91.429	1925	111510	113408	4617	2761	complete clpP
MTPT16	96.421	1900	94951	96849	10512	8629	complete rbcL
MTPT17	95.517	1829	5962	7790	10995	12786	complete ndhA
MTPT18	95.645	1814	5977	7790	8083	6307	complete ndhA
MTPT19	95.678	1712	134080	135789	20947	19289	partial rrn16S; partial trnV
MTPT20	95.678	1712	30621	32330	19289	20947	partial rrn16S; partial trnI_tRNA-Ile
MTPT21	89.116	1709	42576	44240	1	1650	partial matK; partial trnK
MTPT22	97.469	1699	136424	138122	18891	17225	complete rps12; partial trnV
MTPT23	97.469	1699	28288	29986	17225	18891	complete rps12; partial rps7; partial trnI_tRNA-Ile
MTPT24	97.613	1634	138285	139913	17238	15628	complete ndhB
MTPT25	97.613	1634	26497	28125	15628	17238	partial trnI_tRNA-Ile

Number	Identity(%)	Alignment Length(bp)	Chloroplast Genome Start	Chloroplast Genome End	Mitochondrial Genome Start	Mitochondrial Genome End	MTPT Annotation
MTPT26	92.427	1545	50986	52517	11794	10324	partial atpA; partial trnR
MTPT27	93.693	1316	107883	109191	14312	13024	complete rpl33; complete rps18
MTPT28	97.814	1281	144842	146122	1276	1	partial rps19; partial ycf2_Ycf2
MTPT29	97.814	1281	20288	21568	1	1276	partial trnI_tRNA-Ile; partial ycf2_Ycf2
MTPT30	96.452	1240	86256	87495	333	1557	complete rps4; partial trnS
MTPT31	93.053	1166	71607	72727	17271	18427	complete trnD; complete trnE; complete trnY
MTPT32	95.652	1012	62985	63987	5743	4747	partial rpoC1
MTPT33	95.644	1010	44407	45408	30013	29013	partial trnK
MTPT34	95.846	987	75492	76470	19818	18840	partial psbC
MTPT35	96.104	924	1444	2367	30125	31040	IGS(rps19-rps15)
MTPT36	93.348	887	110538	111407	5475	4605	complete clpP; partial rps12
MTPT37	74.183	887	32050	32913	18264	17406	partial rrn16S; partial trnI_tRNA-Ile
MTPT38	74.183	887	133497	134360	17406	18264	partial rrn16S
MTPT39	98.746	877	132963	133839	21815	20947	partial rrn16S
MTPT40	98.746	877	32571	33447	20947	21815	partial rrn16S; partial trnI_tRNA-Ile
MTPT41	86.851	867	74496	75357	20601	19810	partial psbC; partial psbD; partial trnT
MTPT42	94.356	815	69697	70503	15869	16668	complete petN; partial trnC
MTPT43	91.276	768	88482	89249	24762	24036	complete trnL_tRNA-Leu
MTPT44	92.689	766	11	764	11836	12570	IGS(rps19-rps15)
MTPT45	94.645	747	87673	88419	25477	24749	complete trnL_tRNA-Leu
MTPT46	95.528	738	76743	77480	25519	26237	complete psbZ; complete trnG
MTPT47	91.245	731	13463	14193	13798	13101	IGS(rpl32-rps19)
MTPT48	94.528	731	58553	59266	15706	14978	partial rpoC2
MTPT49	87.169	717	16420	17110	15415	14739	complete rpl23; partial trnI_tRNA-Ile
MTPT50	87.169	717	149300	149990	14739	15415	complete rps19; partial rpl2
MTPT51	88.764	712	46879	47566	28983	28305	IGS(rps16-trnQ)

Number	Identity(%)	Alignment Length(bp)	Chloroplast Genome Start	Chloroplast Genome End	Mitochondrial Genome Start	Mitochondrial Genome End	MTPT Annotation
MTPT52	94.895	666	41067	41726	25573	26221	partial psbA; partial trnN
MTPT53	96.6	647	123022	123668	16251	16893	partial rpl16
MTPT54	92.857	644	41932	42575	26222	26845	partial matK; partial trnK
MTPT55	96.044	632	70543	71174	16657	17270	complete psbM
MTPT56	94.558	588	124045	124625	16907	17488	IGS(rpl16-rps3)
MTPT57	98.273	579	4333	4911	23802	23224	IGS(rps19-rps15)
MTPT58	90.138	578	122108	122675	15710	16259	partial rpl14; partial rps8
MTPT59	87.197	578	11881	12456	17193	16649	complete trnL_tRNA-Leu
MTPT60	98.043	562	11269	11830	17754	17193	partial ccsA
MTPT61	98.37	552	106350	106901	15154	14603	complete petG; complete petL
MTPT62	90	550	96863	97411	8117	8626	IGS(rbcL-accD)
MTPT63	91.075	493	71144	71631	2187	2659	IGS(psbM-trnD)
MTPT64	81.78	472	106915	107381	27110	26686	complete trnW; partial trnP
MTPT65	81.602	462	74045	74496	9327	9731	partial psbD; partial trnT
MTPT66	95.964	446	135815	136258	19323	18882	partial trnV
MTPT67	95.964	446	30152	30595	18882	19323	partial trnI_tRNA-Ile
MTPT68	90.255	431	12823	13245	21315	21734	partial rpl32; partial trnI_tRNA-Ile
MTPT69	97.418	426	73388	73812	18429	18854	partial trnT
MTPT70	83.491	424	47851	48264	17749	18153	partial psbK; partial trnQ
MTPT71	91.232	422	45387	45790	10370	9957	complete rps16
MTPT72	98.792	414	10760	11173	18160	17747	partial ccsA
MTPT73	93.237	414	46476	46886	9735	9335	IGS(rps16-trnQ)
MTPT74	100	413	124780	125192	17478	17890	partial rps3
MTPT75	91.111	405	3560	3963	31030	31416	IGS(rps19-rps15)
MTPT76	94.937	395	50590	50984	12280	11900	complete trnG
MTPT77	92.837	363	131852	132209	4038	3693	IGS(trnA-rrn16S)

Number	Identity(%)	Alignment Length(bp)	Chloroplast Genome Start	Chloroplast Genome End	Mitochondrial Genome Start	Mitochondrial Genome End	MTPT Annotation
MTPT78	92.837	363	34201	34558	3693	4038	partial trnI_tRNA-Ile
MTPT79	98.588	354	4900	5253	21764	21411	IGS(rps19-rps15)
MTPT80	87.755	343	100941	101277	25214	24892	IGS(ycf4-cemA)
MTPT81	98.171	328	125796	126123	11828	11501	IGS(rpl22-rrn5S)
MTPT82	98.171	328	40287	40614	11501	11828	partial trnN
MTPT83	94.785	326	107317	107641	14606	14289	partial trnP
MTPT84	91.25	320	47528	47834	9333	9015	partial trnQ
MTPT85	95.899	317	5631	5947	21390	21075	partial rps15
MTPT86	97.377	305	101370	101674	24893	24589	partial cemA
MTPT87	88.435	294	117430	117720	4343	4616	partial petB
MTPT88	94.097	288	115993	116279	1896	1621	partial psbH; partial psbN
MTPT89	96.864	287	116365	116651	342	56	IGS(psbH-petB)
MTPT90	87.857	280	73098	73376	9059	9318	partial trnT
MTPT91	95.29	276	109359	109634	13020	12751	partial rpl20
MTPT92	91.76	267	16164	16430	24318	24062	partial trnI_tRNA-Ile
MTPT93	91.76	267	149980	150246	24062	24318	partial rpl2
MTPT94	90.114	263	48400	48662	28318	28073	IGS(trnQ-psbI)
MTPT95	90.234	256	48663	48918	13778	13541	complete psbI
MTPT96	89.52	229	45951	46169	9964	9745	IGS(rps16-trnQ)
MTPT97	90.826	218	68381	68585	25384	25172	IGS(rpoB-trnC)
MTPT98	95.939	197	104508	104703	21783	21979	complete psbJ
MTPT99	98.352	182	105185	105366	7631	7812	partial psbE
MTPT100	88.953	172	49653	49816	27852	27681	IGS(psbI-trnG)
MTPT101	93.567	171	14349	14519	13115	12952	IGS(rpl32-rps19)
MTPT102	99.398	166	34545	34710	3696	3531	partial trnI_tRNA-Ile
MTPT103	99.398	166	131700	131865	3531	3696	IGS(trnA-rrn16S)

Number	Identity(%)	Alignment Length(bp)	Chloroplast Genome Start	Chloroplast Genome End	Mitochondrial Genome Start	Mitochondrial Genome End	MTPT Annotation
MTPT104	86.667	150	39066	39215	18909	18765	IGS(rrn4.5S-trnN)
MTPT105	86.667	150	127195	127344	18765	18909	IGS(rpl22-rrn4.5S)
MTPT106	95.89	146	125194	125339	2	147	partial rps3
MTPT107	93.056	144	89328	89467	26407	26264	IGS(trnL_tRNA-Leu-ndhJ)
MTPT108	99.138	116	68190	68305	161	46	IGS(rpoB-trnC)
MTPT109	97.03	101	151360	151460	13149	13049	complete trnH_tRNA-His
MTPT110	97.03	101	14950	15050	13049	13149	complete trnH_tRNA-His
MTPT111	85.567	97	130164	130260	15691	15595	partial rrn23S
MTPT112	85.567	97	36150	36246	15595	15691	partial rrn23S
MTPT113	98.81	84	34108	34191	4112	4029	partial trnI_tRNA-Ile
MTPT114	98.81	84	132219	132302	4029	4112	IGS(trnA-rrn16S)
MTPT115	96.386	83	126419	126501	9870	9788	IGS(rpl22-rrn5S)
MTPT116	96.386	83	39909	39991	9788	9870	partial trnN
MTPT117	98.667	75	89328	89402	23970	23896	IGS(trnL_tRNA-Leu-ndhJ)
MTPT118	96.667	60	104790	104849	7577	7636	partial psbL
MTPT119	100	59	45833	45891	29008	28950	IGS(rps16-trnQ)
MTPT120	97.619	42	68305	68346	58	17	IGS(rpoB-trnC)
MTPT121	100	39	18830	18868	23985	23947	IGS(rpl23-trnI_tRNA-Ile)
MTPT122	100	39	147542	147580	23947	23985	IGS(rps19-rps19)
MTPT123	100	39	49250	49288	13544	13506	IGS(psbI-trnG)
MTPT124	100	36	1116	1151	12562	12597	IGS(rps19-rps15)
MTPT125	97.059	34	90591	90624	4918	4886	partial trnV

Table S8. Results of collinearity analysis between *D. officinale* and seven other Orchidaceae species.

genome1	genome2	total block	genome1 > 1000	genome1 > 2000	genome1 > 3000	genome2 > 1000	genome2 > 2000	genome2 > 3000	genome1_ ident_len	genome1 _cov(%)	genome2_ ident_len	genome2 _cov(%)
D.officinale	*A.fujianica*	32	20	6	2	21	6	2	44452	6.95	44940	7.83
D.officinale	*D.hancockii*	115	90	60	47	90	60	48	353598	55.26	354990	67.75
D.officinale	*D.henanense*	130	114	79	53	114	79	54	439833	68.73	439936	54.48
D.officinale	*D.huoshanense*	126	113	77	53	113	77	53	454844	71.08	455354	69.95
D.officinale	*D.loddigesii*	128	105	61	37	106	61	39	352994	55.16	354811	69.12
D.officinale	*D.nobile*	127	114	84	61	113	85	62	517506	80.87	520516	67.38
D.officinale	*D.wilsonii*	120	104	74	54	104	75	56	425708	66.52	426529	55.9

Table S9. Median Ka/Ks values and selection category for 31 shared mitochondrial genes.

Gene	Median_Ka/Ks	Selection_Category
ccmc	0	Strong Purifying Selection
cox1	0	Strong Purifying Selection
nad3	0	Strong Purifying Selection
rps1	0	Strong Purifying Selection
nad4l	0.0773	Strong Purifying Selection
atp6	0.0865	Strong Purifying Selection
cox3	0.1329	Strong Purifying Selection
atp9	0.1633	Strong Purifying Selection
nad5	0.2524	Strong Purifying Selection
atp1	0.2563	Strong Purifying Selection
rps11	0.2785	Strong Purifying Selection
nad9	0.2859	Strong Purifying Selection
cob	0.3091	Strong Purifying Selection
rps12	0.3273	Strong Purifying Selection
ccmfn	0.3589	Strong Purifying Selection
ccmfc	0.4085	Strong Purifying Selection
rps4	0.425	Strong Purifying Selection
atp8	0.493	Strong Purifying Selection
rps2	0.4967	Strong Purifying Selection
rps10	0.5091	Neutral/Weak Purifying
nad7	0.5211	Neutral/Weak Purifying
rps14	0.5291	Neutral/Weak Purifying
atp4	0.5316	Neutral/Weak Purifying
rps13	0.5396	Neutral/Weak Purifying
rps7	0.555	Neutral/Weak Purifying
rpl5	0.6263	Neutral/Weak Purifying
matr	0.64	Neutral/Weak Purifying
cox2	0.6895	Neutral/Weak Purifying
nad4	0.7082	Neutral/Weak Purifying
mttb	0.7152	Neutral/Weak Purifying
ccmb	0.9604	Neutral/Weak Purifying
